# The global burden of motor neuron disease: a systematic and additional analysis of global burden disease study 2021

**DOI:** 10.1186/s13023-025-04084-6

**Published:** 2025-11-10

**Authors:** Yi-Qi Chen, Tao Yu, Zheng-Qi Song, Qi-Chen Lu, Morgan Jones, Majid Nisar, Jiang-Tao Luo, Yong Xiao, Jia-Liang Lin, Qiu Heng, Jia-Jie Lu, Xiang-Xiang Pan, Hai-Ming Jin, Xue-Qin Bai

**Affiliations:** 1 Department of Radiology, The First Affiliated Hospital of Wenzhou Medical University, Wenzhou, Zhejiang 325000 China; 2 Department of Orthopaedics, The Second Affiliated Hospital and Yuying Children’s Hospital of Wenzhou Medical University, Wenzhou, Zhejiang 325000 China; 3 Spine Unit, The Royal Orthopaedic Hospital, Northfield, Bristol Road South, Birmingham, B31 2AP UK; 4 Orthopedic Department, Swat Medical College and Swat Medical Complex Teaching Hospital, Faiz Abad Road, Saidu Sharif, Swat, Khyber Pakhtunkhwa 19200 Pakistan; 5 Department of Orthopedics, Peking University Third Hospital, Beijing, 100191 China; 6 Medical School, The University of Western Australia, Perth, 6009 Australia; 7 Department of Chemistry, The University of Hong Kong, Hong Kong, 999077 China; 8 Department of Orthopaedic Surgery, Zhongshan Hospital, Fudan University, Shanghai, 200032 China

**Keywords:** Motor neuron disease, Global burden of diseases, Epidemiology trends, Cross-country inequality, Health policy interventions

## Abstract

**Background:**

The last 30 years witnessed significant changes in motor neuron disease (MND) epidemiology. Our study aims to explore the trends and inequality of MND, and predict future changes to 2046.

**Methods:**

We used Global Burden of Disease (GBD) 2021 data and methodologies (including trends, decomposition, inequality, frontier, and prediction) to describe the disease burden of MND of incidence, prevalence, deaths, and disability-adjusted life-years (DALYs).

**Results:**

The case number of incidence, prevalence, deaths and DALYs illustrated an upward trend, however, the age-standardized rate (ASR) for these metrics remained stable. There is substantial variability in MND burden across regions and countries, with higher ASR observed in high sociodemographic index (SDI) regions, particularly the United States, Finland, and the United Kingdom. Gender and age differences also contribute to the disease burden, with males and older populations exhibiting higher incidence rates. The age-period-cohort analysis revealed distinct temporal trends of DALYs across age, period, and birth cohort. Decomposition analysis revealed population aging and growth are critical drivers of the increasing burden. Additionally, the cross-country inequality analysis indicates widening disparities in MND burden, particularly in high SDI regions, from 1990 to 2021. Forecasts for 2046 suggest that while the number of deaths and DALYs may decrease, the incidence and prevalence of MND are expected to continue rising. The frontier analysis further reveals significant differences in performance across countries, with certain high-SDI countries like Sweden and the United Kingdom underperform, indicating that socioeconomic advancements may not always translate into lower disease burden.

**Conclusions:**

The findings from this study underscore the need for more targeted interventions, better diagnostic practices, and international collaboration to address the growing global burden of MND. These insights offer a significant contribution to understanding the trends and regional disparities associated with MND, aiding in the development of global health strategies and public health policies to mitigate this burden.

**Supplementary Information:**

The online version contains supplementary material available at 10.1186/s13023-025-04084-6.

## Introduction

Motor neuron disease (MND), encompassing a rare assortment of neurodegenerative disorders, are marked by the progressive degeneration of both upper and lower motor neurons. This heterogeneous group comprises amyotrophic lateral sclerosis (ALS), spinal muscular atrophy, hereditary spastic paraplegia, primary lateral sclerosis, progressive muscular atrophy, and pseudobulbar palsy, among others [1]. ALS, the most prevalent form of MND, is characterized by relentless muscle weakness and atrophy, ultimately culminating in respiratory failure and mortality within a median span of 3 to 5 years [2].

While prior epidemiological investigations in the United States and Europe have illuminated the incidence, prevalence, and mortality patterns of MND, the findings may not be fully representative of the global population. This is due to the studies’ limited geographical scope and the rarity of the disease, which make comprehensive, large-scale studies challenging to conduct [3–6]. Notably, the incidence of MND varies significantly across age groups, sexes, and geographical regions, peaking in individuals aged 60 to 70 years before declining precipitously [7]. Globally, the standardized incidence rate stands at merely 1.68 (1.50–1.85) per 100,000 person-years [6], highlighting the need for a more comprehensive understanding.

Furthermore, the Global Burden of Disease (GBD) 2016 study and subsequent analyses have underscored the concentration of MND prevalence and mortality in high-income regions, specifically North America, Western Europe, and Australasia [8]. Despite these insights, prior research efforts have either relied on outdated data [9], lacked nuanced analyses of temporal trends [10], or focused on a restricted set of countries and territories [9, 11], thereby limiting their broader applicability.

In this study, leveraging the latest iteration of the GBD 2021, we present an updated and comprehensive assessment of the burden, trends, and disparities associated with MND. Our analysis spans the period from 1990 to 2021, examining key indicators such as incidence, prevalence, deaths, and DALYs, stratified by age, sex, year, and geographical location. This endeavor aims to enhance the global understanding of MND epidemiology and inform targeted interventions and policy formulation.

## Methods

### Data source

The GBD 2021 employed the most up-to-date epidemiological data, complemented by refined and standardized methodologies, to systematically and comprehensively quantify health losses across 369 diseases and injuries, as well as 87 risk factors, stratified by age, sex, and geographical location, encompassing 204 countries and territories. The GBD team is committed to annual updates to ensure the accuracy and relevance of their estimates [12]. The intricacies of the methodologies applied within GBD 2021 have been thoroughly documented in prior publications [13].

To address data gaps and ensure smoothness across age, time, and location, the collected data underwent modeling via spatiotemporal Gaussian process regression. This approach facilitated interpolation in regions with incomplete datasets. Furthermore, to correct for biases stemming from diverse case definitions and study methodologies across regions, a meta-regression framework incorporating Bayesian priors, regularization, and trimming techniques was employed.

From GBD 2021, we extracted estimates and their corresponding 95% uncertainty intervals (UIs) for incidence, deaths, prevalence, and DALYs attributed to MND. All rates reported herein are standardized to per 10,000 population. Additionally, the sociodemographic index (SDI), a composite indicator reflecting income, education, and fertility levels, serving as a proxy for sociodemographic development, was utilized to categorize the 204 countries and territories into five distinct groups: high, high-middle, middle, low-middle, and low, as defined by their SDI values [14].

### Descriptive analysis

To gain a holistic understanding of the burden of MND, we conducted descriptive analyses at the global, regional, and national levels. Specifically, we visually presented the global trends in the number of cases, crude rate, and age-standardized rate (ASR) for incidence, deaths, prevalence, and DALYs related to MND, disaggregated by sex (both sexes, males, and females) and spanning the period from 1990 to 2021. Furthermore, we compared the number of cases and ASR for the aforementioned indicators across global, regional (comprising 54 GBD geographic regions), and national (encompassing 204 countries and territories) levels, as well as within the five SDI groups.

### Trend analysis

In the Trend Analysis section, we initially employed the Estimated Annual Percentage Change (EAPC) to quantify the overarching trend in the burden of MND. Given the importance of standardization when comparing diverse groups with varying age structures or a single group experiencing temporal changes in its age profile, the EAPC-measured trend of the ASR emerges as a more robust metric for monitoring shifts in disease patterns [15]. To derive this metric, we constructed a linear regression model where the natural logarithm of the ASR (ln(ASR)) served as the dependent variable (y), and the calendar year acted as the independent variable (x). Subsequently, the EAPC was calculated using the formula (exp(β)-1) * 100%, with its 95% confidence interval (CI) also being extracted from the model [16]. In interpreting the EAPC estimates, if both the EAPC value and the lower bound of its 95% CI are greater than 0, the ASR is deemed to be in an increasing trend. Conversely, if both the EAPC value and the upper bound of its 95% CI are less than 0, the ASR is considered to be in a decreasing trend. In all other cases, the ASR is classified as stable. This approach ensures a rigorous and standardized methodology for assessing temporal trends in the ASR of MND.

Furthermore, we used age-period-cohort (APC) model to explore the underlying trends in DALYs stratified by age, period, and birth cohort. Typically, the APC model fits a log-linear Poisson model on the Lexis diagram of observed rates and quantifies the additional effects of age, period, and birth cohort. The methodological details of APC model are described in previous literature [17]. The multicollinearity between age, period, and birth cohort inevitably leads to identification issues, making it difficult to estimate the unique effects of each age, period, and birth cohort. To address this issue, the intrinsic estimator (IE) algorithm was used to estimate the coefficients of the APC model. This study employed the IE to solve the APC model, where coefficients greater than 0 indicate increased risk, and those less than 0 indicate decreased risk. The effect coefficients were transformed into natural logarithms to calculate the relative risk (RR), enabling the observation of the effects of age, period, and cohort on MND DALYs trends. The DALYs for MND and population data of each country or region were served as data input for APC model. The data was re-coded into consecutive six 5-year periods (1990–1994, 1995–1999, … , 2015–2019), consecutive 5-year age groups (0–4, 5–9, … , 90–94, 95 plus), consecutive 5-year birth cohorts (1895–1899, 1900–1904, … , 2015–2019) to estimate the overall temporal trend in incidence, prevalence, deaths, and DALYs.

### Cross-country inequality analysis

To ensure evidence-based health planning, we conducted a comprehensive cross-country inequality analysis aimed at monitoring health disparities. Specifically, we employed the Slope Index of Inequality (SII) as a key metric, which was derived from regressing the country-level prevalence of the disease across all age groups against a relative position scale tied to sociodemographic development. To account for heteroscedasticity, a robust linear regression model was applied. This method utilizes iteratively reweighted least squares, giving smaller weights to observations with larger residuals, thus minimizing the influence of outliers and ensuring more stable and reliable trend estimates [18]. This approach allowed us to quantify inequalities in MND at global level and across 21 GBD regions.

Furthermore, we calculated the Health Inequality Concentration Index by numerically integrating the area beneath the Lorenz Concentration Curve. This curve was meticulously fitted using the cumulative relative distribution of populations, ordered by their SDI, and the corresponding incidence, prevalence, deaths, and DALYs attributable to the disease [19, 20]. This methodology provided a robust assessment of the concentration of health burden across nations, enabling us to identify disparities and inform targeted interventions. A negative SII/concentration index indicates that as SDI increases, ASDR decreases, and vice versa. The greater the absolute value of the SII/concentration index, the greater the degree of inequality. Their inequality value and implications are presented in Table 1.

**Table 1:** The changing pattern of inequalities from 1990 to 2021 and their implications [21]

Pattern	Inequality values			Implication
	1990	2021	Trend	
Worsening inequality among lower SDI countries	Negative	Negative	Relative increasing	The inequality index was consistently negative, and its absolute value increased over time. It means the disease burden was consistently higher among countries with lower SDI, and this inequality has widened over time
Improving inequality among lower SDI countries	Negative	Negative	Relative decreasing	The inequality index was consistently negative, but its absolute value decreased over time. It means the disease burden was consistently higher among countries with lower SDI, but this inequality has narrowed over time.
Worsening inequality among higher SDI countries	Positive	Positive	Relative increasing	The inequality index was consistently positive, and its absolute value increased over time. It means the disease burden was higher among countries with higher SDI, and this inequality has widened over time.
Improving inequality among higher SDI countries	Positive	Positive	Relative decreasing	The inequality index was consistently positive, but its absolute value decreased over time. It means the disease burden was higher among countries with higher SDI, but this inequality has narrowed over time.
Shift to higher burden among higher SDI countries	Negative	Positive	/	The inequality index was negative in 1990 but shifted to positive in 2021. It means the disease burden was higher among countries with lower SDI initially, but shifted to being higher among countries with higher SDI by 2021.
Shift to higher burden among lower SDI countries	Positive	Negative	/	The inequality index was positive in 1990 but shifted to negative in 2021. It means the disease burden was higher among countries with higher SDI initially, but shifted to being higher among countries with lower SDI by 2021.

Abbreviations: SDI, sociodemographic index

### Decomposition analysis

To gain a profound understanding of the explanatory factors underpinning the variations in MND incidence, prevalence, deaths, and DALYs from 1990 to 2021, we performed a comprehensive decomposition analysis. This analysis dissected the contributions of population size, age structure, and epidemiological changes to the observed trends [22, 23]. By disentangling these components, we aimed to quantify the specific impact of each factor on the evolution of MND burden over time.

The decomposition methodology enabled us to estimate the number of incidence cases, prevalent cases, deaths, and DALYs attributable to each factor at every location under consideration. The calculation of these metrics for each component was carried out as follows:

${X_{ay,py,ey}} = \sum\nolimits_{i = 1}^{20} {\left( {{a_{i,y}} * {p_y} * {e_{i,y}}} \right)}$(*X* = incidence, prevalence, deaths and DALYs)

Where the ${X_{ay,py,ey}}$ represented *X* based on the factors of age structure, population, and specific year $y$; ${a_{i,y}}$ represented the proportion of population for the age category $i$ of the 20 age categories in year $y$; ${p_{y}}$ represented the total population in year $y$ and ${e_{i,y}}$ represented *X* rate for the age category $i$ in year $y$.

The contribution of each factor to the change in incidence, prevalence, deaths and DALYs from 1990 to 2021 was defined by the effect of one factor changing while the other factors were held constant.

### Predictive analysis

To inform the formulation of effective public health policies and the optimal allocation of healthcare resources, we conducted a predictive analysis of the MND burden in the coming decades. For this purpose, we employed the Bayesian age-period-cohort (BAPC) model, augmented with the integrated nested Laplace approximation (INLA) technique. This advanced approach, which has been shown to outperform the conventional annual percentage change model in terms of coverage and precision, was utilized to forecast the global MND burden until 2046.

The adoption of INLA within the BAPC framework offers several advantages. By approximating marginal posterior distributions, it mitigates the mixing and convergence issues that are often encountered with the Markov Chain Monte Carlo sampling techniques traditionally applied in Bayesian methods [24]. This enhancement ensures more reliable and accurate predictions of the future MND burden, thereby supporting evidence-based decision-making in public health planning.

### Frontier analysis

To assess the interplay between the burden of MND and sociodemographic development, we employed a frontier analysis approach. This methodology aimed to delineate the lowest potentially attainable ASR of incidence, prevalence, deaths, and DALYs for each country or territory, contingent upon its SDI. The frontier serves as a benchmark, indicating the minimal achievable level given a country’s or territory’s development status. The deviation from this frontier, termed the effective difference, highlights potential untapped opportunities for improvement or gains, commensurate with the country’s or territory’s position on the development spectrum.

To accommodate non-linear relationships, we conducted a data envelope analysis utilizing the free disposal hull method. This analysis generated an age-adjusted frontier by SDI, utilizing data spanning from 1990 to 2021 [25]. To account for uncertainty, we implemented a bootstrapping procedure, drawing 1,000 samples with replacement from the entire dataset encompassing all countries and territories across all years. From these bootstrapped samples, we computed the mean incidence, prevalence, deaths, and DALYs at each SDI value.

Subsequently, we employed LOESS (Locally Estimated Scatterplot Smoothing) regression with a local polynomial degree of 1 and a span of 0.2 to produce a smooth frontier line [25]. This approach ensured a robust and visually interpretable representation of the frontier, while mitigating the influence of outliers. To further refine the analysis, super-efficient countries, which may distort the frontier due to exceptional performance, were excluded from the frontier generation process.

## Results

### Incidence, prevalence, deaths and DALYs of MND

Table 2 and Table S1-3 present a comprehensive overview of the case number and ASR of MND incidence, prevalence, deaths, and DALYs for the years 1990 and 2021. Notably, while the absolute case number of incidence, prevalence, deaths, and DALYs exhibit pronounced upward trends. However, the ASR for these metrics were not uniform. While the ASR for incidence and prevalence remained relatively stable, showing only marginal changes, the ASR for deaths and DALYs demonstrated small but consistent increases over the study period.

**Table 2:** The case number and ASR of incidence of MND in 1990 and 2021 for both sexes by SDI regions and by GBD regions, with EAPC from 1990 to 2021

	1990		2021			EAPC (95% CI)	
	Number (95% UI)	ASR (95% UI)	Number (95% UI)		ASR (95% UI)		
Global	36769 (33068 to 41,300)	0.81 (0.72 to 0.90)	64178 (58506 to 70,270)		0.77 (0.70 to 0.84)		−0.10 (−0.15 to −0.05)
**SDI regions**							
High-middle SDI	7479 (6613 to 8484)	0.74 (0.66 to 0.84)	11619 (10486 to 12,802)		0.71 (0.64 to 0.79)		−0.13 (−0.22 to −0.04)
High SDI	15272 (14364 to 16,247)	1.50 (1.40 to 1.59)	30184 (28711 to 31,579)		1.66 (1.58 to 1.75)		0.41 (0.38 to 0.44)
Low-middle SDI	4265 (3534 to 5126)	0.43 (0.35 to 0.53)	7026 (5882 to 8363)		0.40 (0.34 to 0.48)		−0.17 (−0.23 to −0.10)
Low SDI	1835 (1522 to 2212)	0.47 (0.38 to 0.58)	3686 (3054 to 4434)		0.42 (0.35 to 0.52)		−0.35 (−0.43 to −0.27)
Middle SDI	7887 (6740 to 9260)	0.52 (0.44 to 0.62)	11610 (9809 to 13,644)		0.46 (0.39 to 0.54)		−0.43 (−0.51 to −0.35)
**GBD regions**							
Advanced Health System	19185 (17878 to 20,523)	1.27 (1.18 to 1.37)	37053 (35141 to 38,872)		1.52 (1.44 to 1.60)		0.65 (0.61 to 0.69)
Africa	2280 (1896 to 2729)	0.46 (0.38 to 0.58)	4505 (3724 to 5447)		0.41 (0.34 to 0.51)		−0.41 (−0.49 to −0.34)
African Region	1748 (1450 to 2105)	0.46 (0.37 to 0.57)	3583 (2952 to 4358)		0.41 (0.33 to 0.51)		−0.42 (−0.50 to −0.34)
America	8089 (7504 to 8719)	1.26 (1.18 to 1.36)	17781 (16870 to 18,685)		1.40 (1.33 to 1.48)		0.44 (0.40 to 0.48)
Andean Latin America	128 (108 to 149)	0.43 (0.36 to 0.50)	305 (265 to 343)		0.49 (0.43 to 0.55)		0.61 (0.56 to 0.66)
Asia	15463 (13357 to 18,059)	0.56 (0.48 to 0.65)	22712 (19411 to 26,490)		0.48 (0.41 to 0.55)		−0.55 (−0.63 to −0.47)
Australasia	478 (453 to 502)	2.11 (2.00 to 2.22)	1274 (1225 to 1329)		2.60 (2.49 to 2.72)		0.75 (0.68 to 0.81)
Basic Health System	11533 (9955 to 13,406)	0.56 (0.48 to 0.66)	16235 (13835 to 18,905)		0.48 (0.41 to 0.56)		−0.55 (−0.64 to −0.47)
Caribbean	182 (155 to 207)	0.59 (0.50 to 0.67)	377 (340 to 412)		0.75 (0.68 to 0.82)		0.91 (0.86 to 0.96)
Central Africa	210 (173 to 252)	0.43 (0.35 to 0.54)	493 (404 to 595)		0.41 (0.33 to 0.50)		−0.22 (−0.29 to −0.14)
Central Asia	299 (248 to 357)	0.48 (0.39 to 0.59)	411 (339 to 503)		0.44 (0.36 to 0.53)		−0.31 (−0.36 to −0.26)
Central Europe	854 (736 to 984)	0.67 (0.58 to 0.76)	1164 (1046 to 1281)		0.75 (0.67 to 0.82)		0.41 (0.38 to 0.44)
Central Latin America	697 (597 to 807)	0.51 (0.44 to 0.59)	1575 (1393 to 1746)		0.63 (0.56 to 0.70)		0.86 (0.80 to 0.91)
Central Sub-Saharan Africa	175 (143 to 212)	0.45 (0.36 to 0.56)	413 (337 to 500)		0.42 (0.34 to 0.52)		−0.24 (−0.32 to −0.16)
Commonwealth High Income	2810 (2666 to 2969)	2.02 (1.91 to 2.14)	6058 (5765 to 6349)		2.48 (2.36 to 2.61)		0.75 (0.69 to 0.80)
Commonwealth Low Income	679 (560 to 823)	0.43 (0.34 to 0.53)	1210 (989 to 1469)		0.37 (0.30 to 0.46)		−0.47 (−0.54 to −0.40)
Commonwealth Middle Income	4017 (3302 to 4921)	0.40 (0.33 to 0.49)	6978 (5726 to 8418)		0.36 (0.30 to 0.44)		−0.27 (−0.35 to −0.20)
East Asia	7089 (6127 to 8224)	0.65 (0.57 to 0.75)	7700 (6320 to 9120)		0.47 (0.40 to 0.54)		−1.29 (−1.47 to −1.12)
East Asia & Pacific - WB	10714 (9407 to 12,276)	0.66 (0.58 to 0.75)	14591 (12654 to 16,691)		0.53 (0.46 to 0.60)		−0.80 (−0.90 to −0.69)
Eastern Africa	609 (500 to 738)	0.48 (0.38 to 0.61)	1207 (985 to 1487)		0.42 (0.34 to 0.54)		−0.47 (−0.56 to −0.38)
Eastern Europe	1319 (1097 to 1578)	0.56 (0.47 to 0.67)	1727 (1521 to 1937)		0.66 (0.57 to 0.73)		0.73 (0.65 to 0.80)
Eastern Mediterranean Region	1603 (1340 to 1894)	0.49 (0.40 to 0.59)	2979 (2458 to 3555)		0.44 (0.37 to 0.53)		−0.33 (−0.40 to −0.27)
Eastern Sub-Saharan Africa	660 (540 to 805)	0.50 (0.40 to 0.63)	1324 (1079 to 1640)		0.44 (0.35 to 0.56)		−0.49 (−0.58 to −0.40)
Europe	10882 (10069 to 11,723)	1.17 (1.08 to 1.26)	19078 (17992 to 20,112)		1.43 (1.35 to 1.53)		0.76 (0.71 to 0.81)
Europe & Central Asia - WB	11087 (10239 to 11,975)	1.14 (1.05 to 1.24)	19375 (18258 to 20,470)		1.39 (1.31 to 1.48)		0.74 (0.68 to 0.79)
European Region	11162 (10307 to 12,055)	1.14 (1.05 to 1.23)	19542 (18414 to 20,652)		1.38 (1.30 to 1.48)		0.73 (0.68 to 0.78)
High-income Asia Pacific	1645 (1470 to 1808)	0.88 (0.79 to 0.96)	3268 (3042 to 3491)		0.91 (0.84 to 0.99)		0.18 (0.15 to 0.20)
High-income North America	5926 (5606 to 6288)	1.82 (1.71 to 1.93)	12657 (12144 to 13,195)		2.10 (2.02 to 2.19)		0.57 (0.53 to 0.62)
Latin America & Caribbean - WB	2185 (1880 to 2491)	0.60 (0.52 to 0.69)	5172 (4668 to 5650)		0.76 (0.69 to 0.83)		0.89 (0.84 to 0.93)
Limited Health System	5488 (4537 to 6666)	0.41 (0.34 to 0.51)	9635 (7976 to 11,624)		0.38 (0.31 to 0.45)		−0.31 (−0.38 to −0.23)
Middle East & North Africa - WB	1182 (999 to 1384)	0.54 (0.45 to 0.64)	2140 (1778 to 2552)		0.49 (0.42 to 0.58)		−0.31 (−0.37 to −0.25)
Minimal Health System	531 (445 to 633)	0.53 (0.43 to 0.65)	1201 (1009 to 1430)		0.50 (0.41 to 0.61)		−0.20 (−0.26 to −0.14)
North Africa and Middle East	1687 (1447 to 1940)	0.57 (0.48 to 0.67)	3111 (2645 to 3632)		0.55 (0.47 to 0.63)		−0.11 (−0.16 to −0.06)
North America	5926 (5606 to 6287)	1.82 (1.71 to 1.93)	12657 (12144 to 13,195)		2.10 (2.02 to 2.19)		0.57 (0.53 to 0.62)
Northern Africa	525 (439 to 621)	0.49 (0.41 to 0.60)	889 (736 to 1057)		0.45 (0.37 to 0.53)		−0.33 (−0.40 to −0.27)
Oceania	23 (19 to 27)	0.44 (0.37 to 0.52)	47 (39 to 55)		0.39 (0.32 to 0.46)		−0.49 (−0.56 to −0.42)
Region of the Americas	8089 (7504 to 8719)	1.26 (1.18 to 1.36)	17781 (16870 to 18,685)		1.40 (1.33 to 1.48)		0.44 (0.40 to 0.48)
South-East Asia Region	4465 (3681 to 5462)	0.40 (0.33 to 0.49)	7157 (5860 to 8706)		0.36 (0.30 to 0.44)		−0.28 (−0.36 to −0.21)
South Asia	3754 (3086 to 4607)	0.40 (0.32 to 0.49)	6311 (5188 to 7623)		0.37 (0.31 to 0.44)		−0.21 (−0.28 to −0.14)
South Asia - WB	3871 (3185 to 4745)	0.40 (0.33 to 0.49)	6552 (5395 to 7891)		0.37 (0.31 to 0.44)		−0.20 (−0.27 to −0.13)
Southeast Asia	1532 (1262 to 1840)	0.40 (0.33 to 0.48)	2381 (1949 to 2905)		0.35 (0.29 to 0.42)		−0.45 (−0.53 to −0.37)
Southern Africa	346 (286 to 423)	0.49 (0.39 to 0.62)	627 (510 to 769)		0.43 (0.35 to 0.53)		−0.45 (−0.54 to −0.36)
Southern Latin America	381 (331 to 431)	0.80 (0.69 to 0.90)	815 (754 to 881)		1.05 (0.96 to 1.13)		0.92 (0.86 to 0.98)
Southern Sub-Saharan Africa	189 (155 to 231)	0.45 (0.37 to 0.57)	290 (233 to 359)		0.40 (0.33 to 0.50)		−0.42 (−0.52 to −0.32)
Sub-Saharan Africa - WB	1761 (1460 to 2120)	0.46 (0.37 to 0.58)	3626 (2991 to 4408)		0.41 (0.33 to 0.51)		−0.41 (−0.49 to −0.33)
Tropical Latin America	804 (692 to 916)	0.65 (0.56 to 0.74)	2113 (1921 to 2294)		0.86 (0.79 to 0.93)		1.14 (1.07 to 1.21)
Western Africa	590 (492 to 707)	0.44 (0.36 to 0.54)	1290 (1076 to 1548)		0.39 (0.33 to 0.47)		−0.38 (−0.45 to −0.3)
Western Europe	8299 (7796 to 8820)	1.64 (1.54 to 1.75)	15472 (14697 to 16,254)		2 (1.9 to 2.12)		0.7 (0.66 to 0.75)
Western Pacific Region	9555 (8421 to 10,891)	0.71 (0.62 to 0.8)	12836 (11198 to 14,543)		0.56 (0.49 to 0.63)		−0.86 (−0.98 to −0.74)
Western Sub-Saharan Africa	650 (541 to 779)	0.44 (0.36 to 0.54)	1444 (1204 to 1732)		0.39 (0.32 to 0.47)		−0.37 (−0.45 to −0.3)
World Bank High Income	17411 (16347 to 18,548)	1.44 (1.35 to 1.54)	34568 (32861 to 36,206)		1.68 (1.6 to 1.76)		0.56 (0.53 to 0.6)
World Bank Low Income	1253 (1050 to 1486)	0.53 (0.43 to 0.65)	2462 (2044 to 2953)		0.48 (0.39 to 0.58)		−0.37 (−0.45 to −0.29)
World Bank Lower Middle Income	7090 (5858 to 8573)	0.41 (0.34 to 0.51)	11823 (9777 to 14,267)		0.37 (0.31 to 0.45)		−0.32 (−0.39 to −0.24)
World Bank Upper Middle Income	10984 (9507 to 12,740)	0.61 (0.53 to 0.71)	15271 (13175 to 17,519)		0.54 (0.46 to 0.61)		−0.46 (−0.54 to −0.37)

Abbreviations: ASR, age-standardized rate; MND, motor neuron diseases; EAPC, estimated annual percentage change; SDI, sociodemographic index; GBD, Global Burden of Diseases, Injuries, and Risk Factors Study; UI, uncertainty interval; CI, confidence interval

**Fig. 1:**
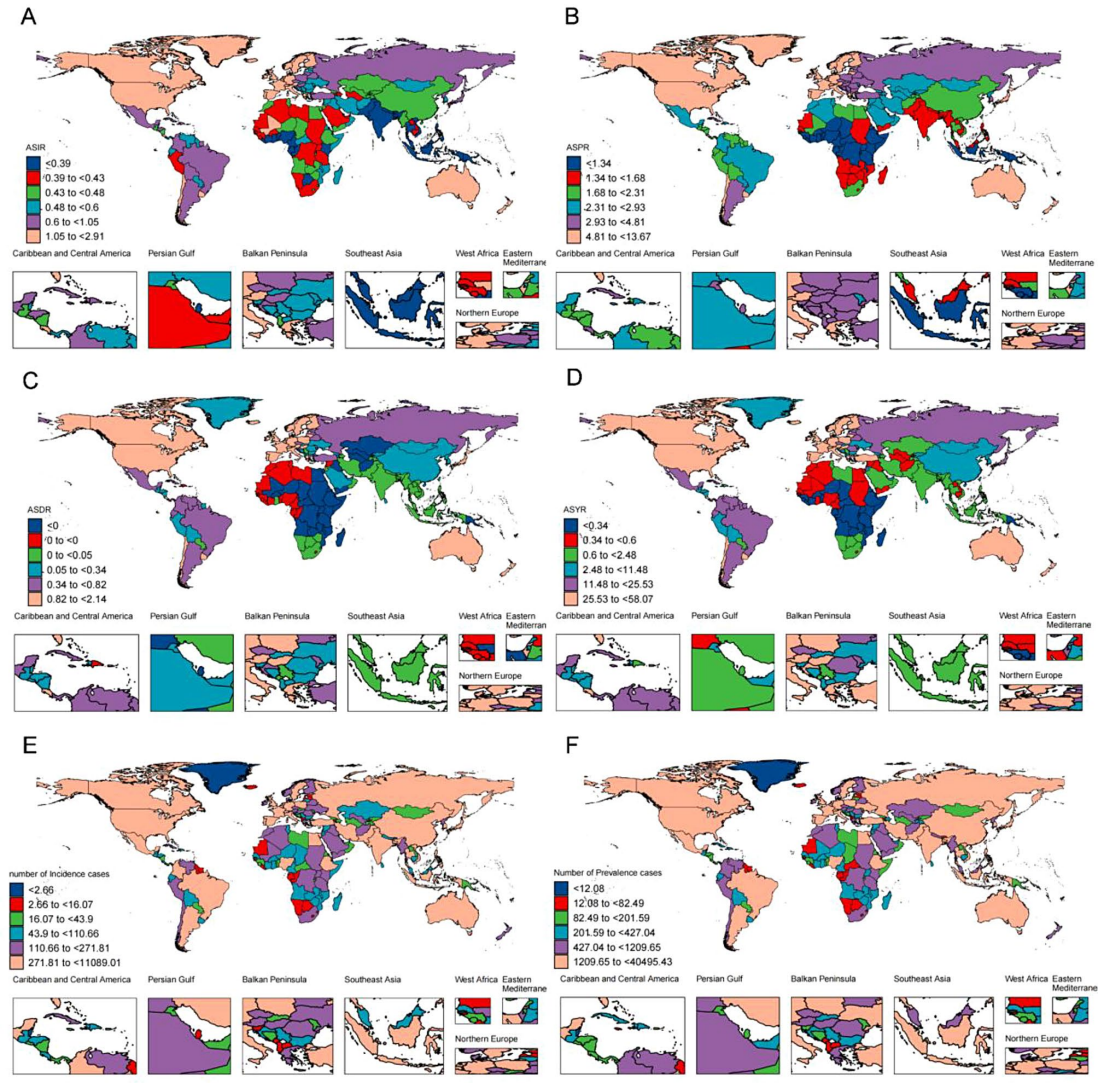
(**A**) The ASR of incidence (**A**), prevalence (**B**), deaths (**C**) and DALYs (**D**) in 2021; (**F**) the case number of incidence (**E**) and prevalence (**F**) in 2021. Abbreviations: ASR, age-standardized rate; DALYs, disability-adjusted life-years

Substantial variations in the incidence, prevalence, deaths, and DALYs of MND are evident at both regional and national scales, as detailed in Table 2 and Table S1-3, along with supplementary Tables S4 to S7, and visually illustrated in Fig. 1 and Fig. S1. Specifically, the United States stands out with the highest case number across all metrics, whereas Finland and the United Kingdom have the highest ASR for deaths and DALYs, and incidence, respectively. Additionally, Canada holds the highest ASR for prevalence. When analyzed by SDI regions, high SDI regions consistently display the greatest number of cases and ASR for incidence, prevalence, deaths, and DALYs (Table 1; Table S1-3; Fig. S6).

Furthermore, our analysis reveals a gender disparity, with males consistently reporting higher number of cases and ASR for incidence, prevalence, deaths, and DALYs in both 1990 and 2021 (Tables S4-7; Fig. S5). Age-wise, the 70–79 age group recorded the highest ASR in 2021, whereas the 65–74 age group contributed the largest case number (Tables S4-7; Fig. S4). These findings underscore the complexity and multifaceted nature of MND burden, necessitating targeted interventions and policies to address these disparities.

### Overall trends in MND burden using broad estimation analysis

Globally, the APC in the ASR of MND incidence, prevalence, deaths, and DALYs from 1990 to 2021 varied substantially, with average increases of −0.1% (95% CI: −0.15% to 0.05%), 0.11% (95% CI: 0.04% to 0.19%), 0.69% (95% CI: 0.57% to 0.80%), and 0.30% (95% CI: 0.23% to 0.37%) per year, respectively (Table 1; Table S1-3; Fig. S4). Regionally, notable increases in ASR were predominantly observed in Tropical Latin America, North America, and Andean Latin America for incidence, prevalence, deaths, and DALYs, whereas East Asia, Oceania, and Northern Africa exhibited the most significant declines (Table 1; Table S1-3; Fig. S10).

At the national level, disease burden trends varied considerably across 204 countries and territories (Fig. S3). Costa Rica, Greece, and Ecuador recorded the highest EAPC for incidence, prevalence, and deaths. Additionally, Ecuador also had the highest EAPC for DALYs. Conversely, Guam demonstrated the lowest EAPC for incidence, prevalence, and DALYs, while Egypt had the lowest EAPC for deaths (Table S4–S7).

From an SDI region perspective, the ASR of incidence, prevalence, and DALYs generally increased across all five SDI regions from 1990 to 2019, with the most pronounced gains observed in high SDI, high SDI, low SDI, and low-middle SDI regions (Table 1; Table S1-3; Fig. S11). Additionally, within the age structure, the ASR of incidence, prevalence, deaths, and DALYs consistently rose among individuals aged over 60 years (Table S4–S7; Fig. S6). Notably, despite males having higher case number and ASR for these metrics, both genders exhibited similar trends from 1990 to 2021 (Fig. S12).

### Cross-country inequality analysis

**Fig. 2:**
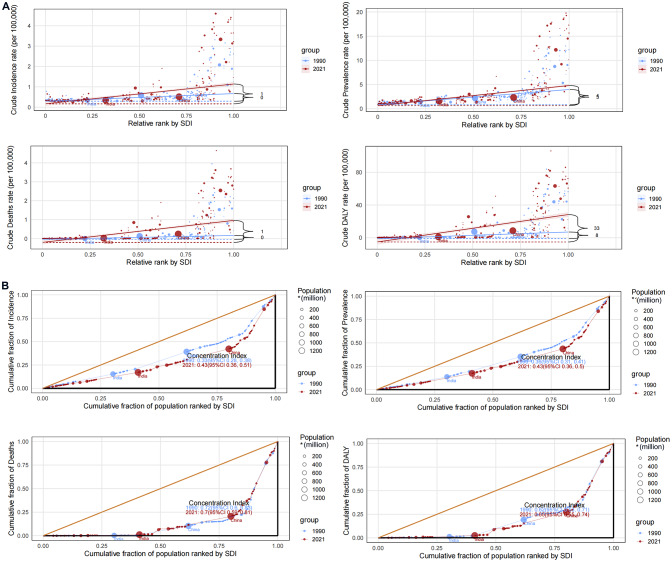
SDI-related health inequality regression (**A**) and concentration (**B**) curves for the incidence, prevalence, deaths and DALYs of MND worldwide from 1990 to 2021. Abbreviations: SDI, sociodemographic index; MND, motor neuron disease; DALYs, disability-adjusted life-years

Substantial absolute and relative inequalities in the burden of MND, linked to SDI, were evident, marked by pronounced temporal increments in the respective indicators (Fig. 2). In 1990, the slope index of inequality revealed a disparity of 0 (incidence), 3 (prevalence), 0 (deaths), and 8 (DALYs) per 100,000 population between nations at the extremities of the SDI spectrum. Notably, this disparity widened in 2021, reaching 1 (incidence), 4 (prevalence), 1 (deaths), and 33 (DALYs) per 100,000 population. Additionally, the concentration index underscored an upward trend in incidence, prevalence, and DALYs from 1990 to 2021, while an opposing trend was observed for deaths, with the index declining over this period.

**Fig. 3:**
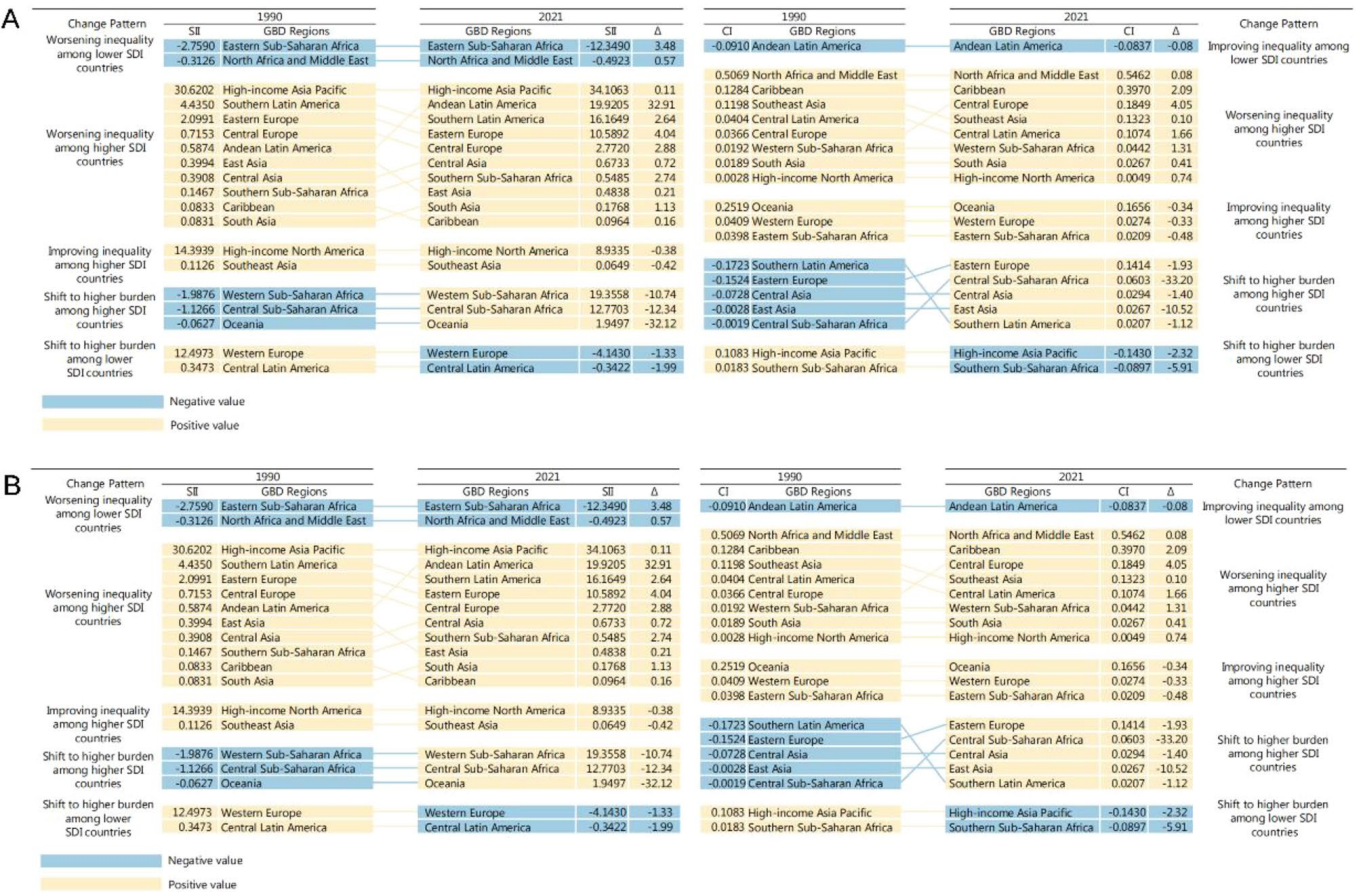
Ranking graphs and change patterns for SII and CI of DALYs (**A**) and deaths (**B**). Abbreviations: SII, slope index of inequality; GBD, global burden of disease; δ, the percentage change of inequality from 1990 to 2021; CI, concentration index; SDI, sociodemographic index; DALYs, disability-adjusted life-years

Regarding DALYs, from 1990 to 2021, inequality in SII and concentration index among high-SDI countries worsened in most regions, with the Andean Latin America suffering the most severe worsening inequality in SII and East Asia showing the most significant deterioration inequality in concentration index. Oceania and Central Sub-Saharan Africa are the higher SDI regions with the most notable shifts toward higher burdens in terms of SII and concentration index, respectively. In 1990 and 2021, High-Income Asia Pacific reported the highest SII, while North Africa and Middle East showed the highest concentration index (Fig. 3A). The change patterns in deaths across GBD regions are similar to those in incidence. Among higher SDI countries, Caribbean experienced the most severe worsening inequality in SII, while East Asia in concentration index. High-income North America reported the largest worsening inequality among both higher and lower SDI countries, and North Africa and Middle East showed the highest concentration index. Central Asia and Eastern Europe exhibit a shift to higher burden among higher SDI countries in SII and concentration index (Fig. 3B). For the incidence, inequality in SII has improved in many regions in both higher SDI and lower SDI countries, with Central Asia demonstrating the most significant improvement among lower SDI countries, while High-income Asia Pacific exhibiting the greatest reduction in equality among higher SDI countries. Concentration index inequality showed worsening trends in most regions from 1990 to 2021. Central Europe reported the most pronounced worsening inequality among higher countries (Fig. S15A). Regionally, the changing patterns of SII and concentration index for prevalence were similar to incidence and prevalence, with Western Sub-Saharan Africa presenting the largest worsening inequality among higher SDI countries in SII and Eastern Europe showing the largest worsening inequality among higher SDI in concentration index. Among higher SDI countries, Southern Latin America exhibited a shift to higher burden in both SII and concentration index (Fig. S15B).

### Age-period-cohort analysis

**Fig. 4:**
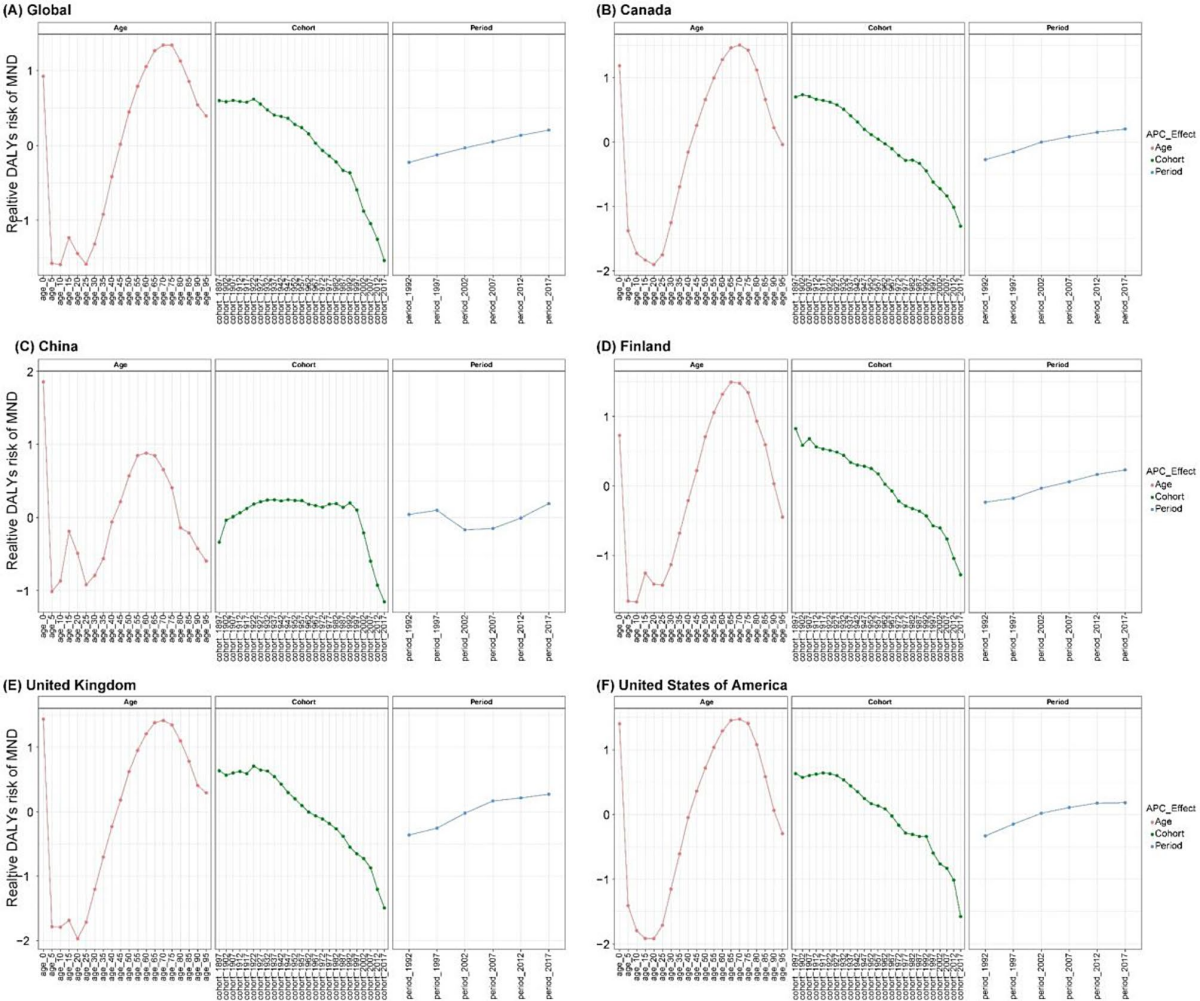
(**A**) the effect of age, period, and cohort on the relative risk of MND DALYs; (**B-F**) age-period-cohort effects on the relative risk of MND DALYs in five exemplar countries. Abbreviations: MND, motor neuron disease; DALYs, disability-adjusted life-years

The results of APC model on the DALYs of MND globally were illustrated in Fig. 4A. After controlling for period and cohort effects, the age effect significantly influenced the risk of MND DALYs. The relative risk of DALYs first increased and then decreased, with the highest risk observed in the 70–74 and 75–79 age groups. Notably, the relative risk in the 0–4 age group is significantly higher than that of individuals under 30 years old. Controlling for age and period effects, cohort effects significantly influenced DALYs. Earlier birth cohorts exhibited higher incidence and prevalence than later cohorts, with relative risks markedly decreasing from the 1895–1899 cohort to the 2015–2019 cohort. Period effects, after adjusting for age and cohort, showed a slight upward trend in DALYs risk, peaking in 2015–2019.

To avoid interference in the analysis caused by countries with missing data, we selected five countries with representative burdens for the APC model analysis, rather than choosing typical countries from the five SDI quintiles. Fig. 4B-F presents the results of APC effects for five typical countries. Among them, the age, period, and birth cohort effects on MND DALYs in the United States, Canada, Finland, and the United Kingdom are similar to the global findings. The relative risk of DALYs rises markedly during middle age (under 60 years) and decreases with advancing age. In contrast to global trends and the other four typical countries, individuals aged 15–19 show a relatively higher risk among young populations. Controlling for age and period effects, little change was observed in relative risk for cohorts born before 1995, while a substantial decrease in DALYs risk was noted for cohorts born afterward. This pattern likely indicates temporal improvements in environmental, medical, or socio-cultural factors. Period-related impacts on DALYs relative risk exhibit fluctuations, which may correspond to economic shifts or targeted public health measures.

### Decomposition analysis

Fig. S14 presents a striking augmentation in global incidence, prevalence, deaths, and DALYs, with the most pronounced surge observed in the high SDI region. Analysis reveals that aging, population dynamics, and epidemiological shifts contribute distinctly to these increases, accounting for 38.64%, 64.44%, and −2.08% of the global rise in incidence; 23.24%, 80.51%, and −3.75% for prevalence; 34.97%, 42.40%, and 22.62% for deaths; and 31.78%, 47.56%, and 20.67% for DALYs, respectively (Table S8). Notably, the magnitudes of these contributions vary significantly across incidence, prevalence, deaths, and DALYs, highlighting the complex interplay between aging, population growth, and epidemiological changes in shaping the global burden of disease, particularly within varying SDI contexts.

### Predictive analysis on MND burden to 2046

Fig. S13 showcases the projected trends in case number and ASR of incidence, prevalence, deaths, and DALYs for MND up to the year 2046. Globally, the forecast indicates an annual increase in both case number and ASR for incidence and prevalence, whereas a decrease is anticipated for case number and ASR of deaths and DALYs over the same period. The specific numerical projections are detailed in Table S9, providing a comprehensive overview of the expected future burden of MND.

### Frontier analysis

**Fig. 5:**
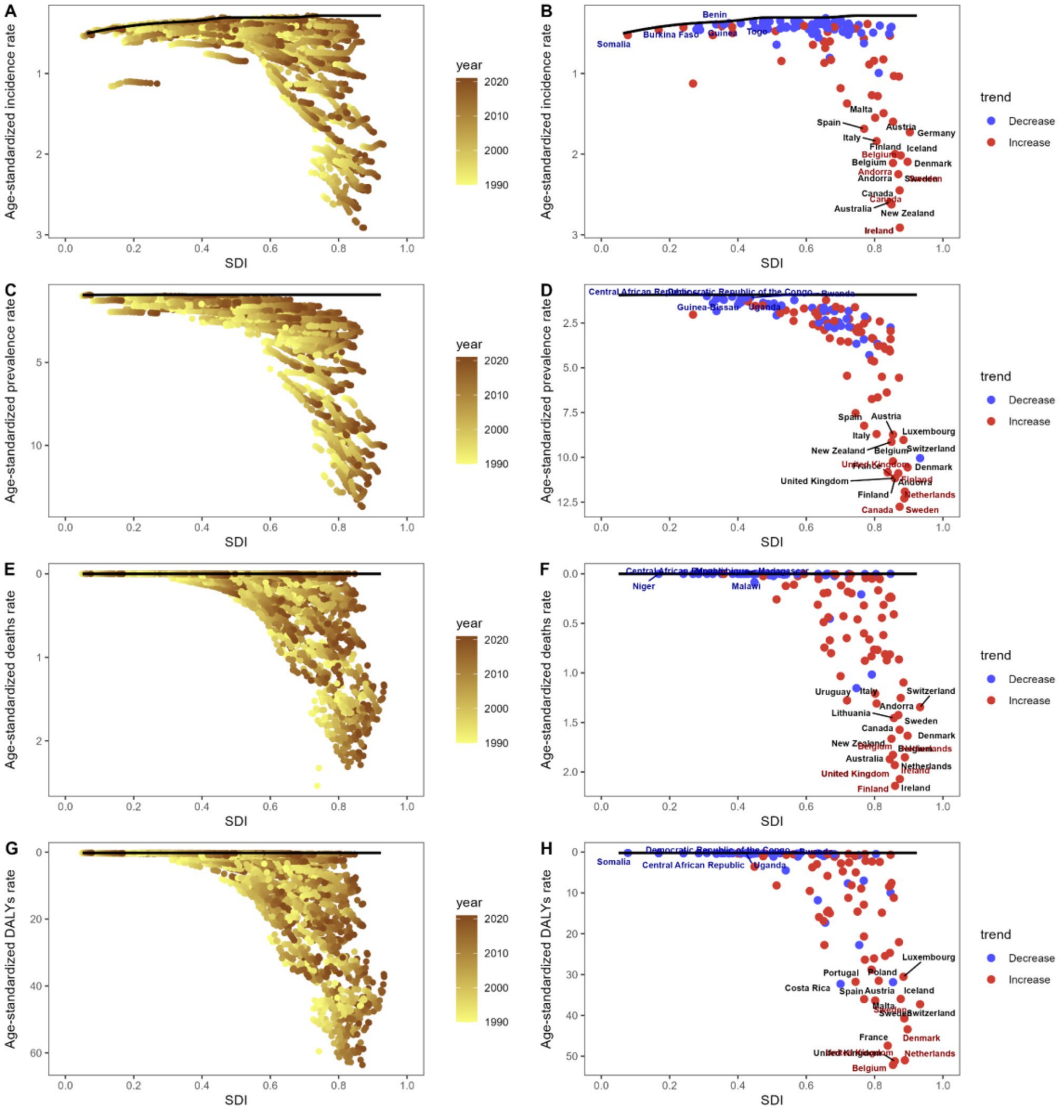
Frontier analysis based on SDI and age-standardized incidence (**A**), prevalence (**C**), deaths (**E**) and DALYs (**G**) rate from 1990 to 2021; frontier analysis based on SDI and age-standardized incidence (**B**), prevalence (**D**), deaths (**F**) and DALYs (**H**) rate in 2021. The frontier is delineated in black color; countries and territories are represented as dots. The top 15 countries with the largest effective difference are labeled in black; examples of frontier countries with low SDI and low effective difference are labeled in blue, and examples of countries with high SDI and relatively high effective difference are labeled in red. Red dots indicate an increase in ASR from 1990 to 2016; blue dot indicate a decrease in ASR from 1990 to 2016. Abbreviations: ASR, age-standardized rate; SDI, sociodemographic index; DALYs, disability-adjusted life-years

To gain a deeper comprehension of the achievable potential for improvement in the ASR of incidence, prevalence, deaths, and DALYs associated with MND, we conducted a frontier analysis utilizing data spanning from 1990 to 2021 (Fig. 5A, C, E, G). This analysis quantifies the “effective difference,” or the gap from the optimal frontier, for each country and territory based on their 2021 data (Fig. 5B, D, F, H). In general, we observed that the effective difference for a given SDI tended to narrow and exhibit less variation as SDI increased. Countries with distinct combinations of low SDI and minimal effective difference, high SDI and relatively large effective difference, or the largest effective differences are outlined in Table S10, providing a nuanced understanding of the diverse performance across nations.

## Discussion

Our study offers an up-to-date comprehensive analysis of the global, regional, and national trends in the incidence, prevalence, deaths, and DALYs of MND from 1990 to 2021. Employing a multifaceted approach, including trend, decomposition, inequality, frontier, and predictive analyses, we uncover nuanced insights into the evolving landscape of MND worldwide. Despite inter-country variations, our findings underscore a consistent upward trajectory in the global burden of MND over the past three decades. Our decomposition analysis powerfully illustrates that population growth, and particularly the aging of the global population, is the primary driver of the increasing total case numbers and deaths from MND. This emphasizes that while we may see improvements in ASR due to therapeutic advancements, the absolute burden of the disease will continue to rise globally as the number of individuals in high-risk age groups increases. This finding demands a forward-looking approach to health policy, focusing on preparing healthcare systems for a larger population of older adults living with neurodegenerative diseases.

Cross-country disparities are evident, with high SDI countries shouldering a disproportionately heavy burden of MND. Alarmingly, these inequalities have widened over time, highlighting the urgent need for tailored health policies to address the unique challenges faced by diverse nations and regions. Although projections indicate a slight annual decrease in the case number and ASR for incidence and prevalence from 2022 to 2046, the corresponding figures for deaths and DALYs portend a persistent and formidable challenge in managing MND globally.

The frontier analysis provides a stark contrast, revealing that while low-SDI countries exhibit low effective differences, certain high-SDI nations perform surprisingly poorly. In 2021, MND accounted for substantial global health outcomes, with the United States topping the list in case number and Europe and North America leading in ASR. These findings align with previous epidemiological studies, reinforcing the consistent pattern of higher MND burden in high-SDI regions.

The striking regional variations in MND burden, with high-SDI regions consistently exhibiting a higher incidence and prevalence, likely reflect a complex interplay of genetic, environmental, and diagnostic factors. The lower relative risk of DALYs and incidence in East and South Asian populations, as noted in prior studies, aligns with our findings and suggests a potential genetic susceptibility that is less common in these populations. The observed differences could be partly explained by distinct genetic risk factor profiles between populations. For example, while the C9orf72 repeat expansion is the most frequent mutation in European ALS patients, the SOD1 mutation is the most common in East Asian patients [26]. Beyond genetics, environmental influences may also play a role. Variations in exposure to environmental toxins, lifestyle, and even climatic factors could contribute to the geographic disparities. While the ASR of deaths and DALYs appears to be increasing globally, particularly in high-SDI regions, the opposite trend is observed for incidence and prevalence at the global level. However, within SDI regions, the ASR of incidence and prevalence in high-SDI regions has significantly risen, underscoring the need for targeted interventions. The paradoxical finding of lower disease burden despite better healthcare access in high-SDI countries may be attributed to factors such as early or incorrect diagnosis, population aging, and the application of varying diagnostic criteria over time.

As shown in decomposition analysis, population growth, a ubiquitous driver across all SDI regions, exerts a more pronounced effect in regions with both lower and higher SDI, due to factors like aging. Age, period, and birth cohort effects represent three ways individuals and their societies change over time [27]. Thus, exploring the temporal trends of MND burden, with a particular focus on its association with these effects, can enhance our understanding of disease epidemiology. The observed age-specific DALYs trend, which peaks in the 70–79 age group, is a crucial finding that reflects the neurobiological basis of MND. This pattern aligns with the “multiple-hit hypothesis”, which posits that disease onset requires the accumulation of multiple insults—genetic, environmental, and age-related—over a lifetime. As individuals age, cellular mechanisms for maintaining proteostasis and repairing DNA become less efficient, increasing vulnerability to the protein aggregation and neurodegeneration characteristic of MND. The decline in DALYs after age 80, while potentially influenced by diagnostic challenges in the very elderly, may also be a reflection of survivorship bias, where individuals who develop MND later in life may have a more slowly progressing form of the disease. This observation is consistent with our trend analysis and earlier results, showing that DALYs associated with ASR and case number are highest in the 70–74 age group [28]. The rapid decline in DALYs after the age of 80 should be interpreted with caution, possibly due to increased diagnostic challenges in elderly patients and the higher prevalence of fatal complications. The high prevalence and poorer prognosis in elderly patients may also contribute to the decline in DALYs. Another minor peak in DALYs appears during the post-neonatal period ( < 5 years). The high relative risk of DALYs in early childhood is thought to result from the inclusion of MND other than ALS, which primarily occur in childhood, such as spinal muscular atrophy and hereditary spastic paraplegia, in the analysis. Moreover, the updates to epidemiological data within the GBD framework contribute to the observed rise in relative risk. In terms of the cohort effect, the relative DALYs risk declines as cohorts progress, suggesting that earlier-born individuals have a higher DALYs risk than later-born ones. This could be attributed to later-born individuals having access to better healthcare and more comprehensive health education than earlier generations. Interestingly, in the APC analysis of the five exemplar countries, the birth cohort effect observed in China was significantly different from that of the other four countries and the global trend. While the lower relative risk of DALYs in China may suggest a potential link to ethnicity, it’s crucial to consider that these differences are likely multifactorial, encompassing genetic, environmental, and lifestyle influences. Several epidemiological studies have revealed differences in mortality, incidence, and various disease subtypes among different ethnic groups. A 2020 study indicated that the risk of ALS is higher in White populations compared to Black and Asian populations [29]. Another study found that the incidence of ALS is higher in populations of European descent (e.g., the US and Europe) compared to East and South Asian populations [6]. Furthermore, ALS subtypes associated with higher disability and mortality rates are more common in regions with populations of European descent [30, 31].

Our study quantified the cross-country inequalities in MND burden and their temporal trends from 1990 to 2021. The SII and concentration index of incidence, prevalence and DALYs revealed that inequalities are worsening globally and in most higher SDI countries and regions. In contrast, inequality in deaths primarily worsened in lower SDI regions. This pattern aligns with the findings from our trend analysis. Cross-country inequality analysis underscores the critical role of identifying and addressing disparities to inform policymaking and foster international collaboration.

In conclusion, our study underscores the pressing need for a nuanced understanding of the evolving MND burden and its underlying determinants. Flexible health policies tailored to the unique circumstances of different nations and regions are imperative to tackle the diverse challenges posed by MND. Furthermore, continuous efforts to improve diagnostic accuracy, enhance access to healthcare, and mitigate the impact of population aging are crucial to stem the tide of this growing global health threat.

In our discussion, despite the anticipated decline in case number and ASR of deaths and DALYs for both sexes until 2046, the projected rise in incidence and prevalence underscores the formidable task of managing MND and the substantial disease burden they impose. This augmentation in incidence and prevalence, particularly from 2021 to 2046, despite advancements in the diagnosis and prognosis of ALS, underscores the incurable nature of ALS and its intimate link to aging [2]. The projected decline in deaths and DALYs by 2046 may stem from therapeutic advancements, including the widespread adoption of tracheostomy [32] and gastrostomy [33], as well as the introduction of novel medications like riluzole [34] and edaravone [35].

Our frontier analysis offers a juxtaposition to the daunting trends and inequality analyses, provideing a stark measure of a nation’s effectiveness in managing MND relative to its socioeconomic development. Notably, several SDI countries exhibit substantial deviations from the frontier in terms of incidence, prevalence, deaths, and DALYs. While low-SDI countries perform relatively close to their potential frontier, demonstrating smaller effective gaps despite resource constraints. In contrast, the underperformance of some high-SDI nations like Sweden and the United Kingdom is a critical finding. This suggests that high income and strong health infrastructure do not automatically translate to optimal MND care. Potential explanations include delays in diagnosis due to the rarity of the disease, fragmented specialist care, or unequal access to novel, expensive therapies like riluzole and edaravone within these seemingly advanced systems. This underscores the need for high-SDI nations to re-evaluate their public health policies and ensure that even rare diseases receive the specialized, integrated care they require. Uncovering the success factors and impediments to progress is paramount for guiding efforts aimed at alleviating the MND burden.

## Restrictions

Recognizing the limitations of our study is essential. Firstly, the GBD estimates rely on available data sources, and their accuracy hinges on the quality of these data across nations. The potential inadequacy of reporting and predictability of MND data across 204 countries could introduce inaccuracies. Secondly, the GBD data sources do not encompass all populations or regions, providing a generalized view of selected regions. To enhance the reliability of disease burden estimates and precision of studies derived from them, increased international cooperation, universal adoption of diagnostic standards in underdeveloped countries, and more efficient health data collection from diverse sources are warranted. Nevertheless, our analyses on trends, decomposition, inequality, frontier, and prediction offer invaluable insights into the epidemiology of MND.

## Conclusions

Our analysis offers a comprehensive and timely overview of the global burden imposed by MND, with a keen focus on salient regional disparities and the pivotal influence of sociodemographic factors in shaping this burden. Despite notable variations in incidence, prevalence, mortality rates, and DALYs across the globe, our findings underscore a discernible upward trend in the overall global burden of MND. As the global population ages inexorably, it is anticipated that the burden of MND will escalate further, emphasizing the imperative for strengthened public health strategies, optimized diagnostic, therapeutic, and prognostic modalities, and a concerted effort to mitigate health inequities.

Future research endeavors should prioritize bridging data gaps in underrepresented regions and delve deeper into the genetic and environmental underpinnings of MND. Such endeavors will not only enrich our understanding of these complex disorders but also inform the development of more targeted and effective global health interventions aimed at alleviating the burden of MND worldwide.

## Electronic supplementary material

Below is the link to the electronic supplementary material.

Supplementary Material 1

## Data Availability

The complete data of this study can be found in the supplementary materials. Data will be made available on request.
