# Supplementary material for: The global burden of motor neuron disease: a systematic and additional analysis of global burden disease study 2021
Source: Orphanet J Rare Dis. 2025 Nov 10;20:568. doi: 10.1186/s13023-025-04084-6 (PMC12604214; doi:10.1186/s13023-025-04084-6)
Supplement: Supplementary file 1 — Supplementary Material 1 [file 13023_2025_4084_MOESM1_ESM.docx]

**Legend**

**Figure S1.** The case number of deaths (A) and DALYs (B) in 2021. abbreviations: DALYs, disability-adjusted life-years.

**Figure S2.** The trend in case number of incidence (A), prevalence (B), deaths (C) and DALYs (D) from 1990 to 2021. Abbreviations: DALYs, disability-adjusted life-years.

**Figure S3.** (A) The trend in ASR of incidence (EAPC) from 1990 to 2021; (B) The trend in ASR of prevalence (EAPC) from 1990 to 2021; (C) The trend in ASR of deaths (EAPC) from 1990 to 2021; (D) The trend in ASR of DALYs (EAPC) from 1990 to 2021. Abbreviations: ASR, age-standardized rate; EAPC, estimated annual percentage change; DALYs, disability-adjusted life-years.

**Figure S4.** The global trend of case number and ASR of incidence (A), prevalence (B), deaths (C) and DALYs (D) of MND from 1990 to 2021. Abbreviations: ASR, age-standardized rate; DALYs, disability-adjusted life-years; MND, motor neuron disease.

**Figure S5.** The global trend of ASR (A) and case number (B) of incidence, prevalence, deaths, and DALYs by age from 1990 to 2021. Abbreviations: ASR, age-standardized rate; DALYs, disability-adjusted life-years.

**Figure S6.** The ASR (A) and case number (B) of incidence, prevalence, deaths, and DALYs of MND by age in 2021. Abbreviations: ASR, age-standardized rate; MND, motor neuron disease; DALYs, disability-adjusted life-years.

**Figure S7.** The ASR (A) and case number (B) of incidence, prevalence, deaths, and DALYs of MND by sex in 2021. Abbreviations：ASR, age-standardized rate; MND, motor neuron disease; DALYs, disability-adjusted life-years.

**Figure S8.** The ASR (A) and case number (B) of incidence, prevalence, deaths, and DALYs of MND by GBD regions in 2021. Abbreviations: ASR, age-standardized rate; MND, motor neuron disease; DALYs, disability-adjusted life-years; GBD, Global Burden of Diseases, Injuries, and Risk Factors Study.

**Figure S9.** The ASR (A) and case number (B) of incidence, prevalence, deaths, and DALYs of MND by SDI region in 2021. Abbreviations: ASR, age-standardized rate; MND, motor neuron disease; DALYs, disability-adjusted life-years; SDI, sociodemographic index.

**Figure S10.** The clustering of the trend from 1990 to 2021 by GBD regions. Abbreviations: GBD, Global Burden of Diseases, Injuries, and Risk Factors Study.

**Figure S11.** The trend of ASR (A) and case number (B) of incidence, prevalence, deaths and DALYs of MND by SDI region from 1990 to 2021. Abbreviations: ASR, age-standardized rate; DALYs, disability-adjusted life-years; MND, motor neuron disease; SDI, sociodemographic index.

**Figure S12.** The trend of ASR (A) and case number (B) of incidence, prevalence, deaths and DALYs of MND by sex from 1990 to 2021. Abbreviations: ASR, age-standardized rate; MND, motor neuron disease; DALYs, disability-adjusted life-years.

**Figure S13.** (A) The predicted case number and ASR of incidence to 2046; (B) The predicted case number and ASR of prevalence to 2046; (C) The predicted case number and ASR of deaths to 2046; (D) The predicted case number and ASR of incidence to 2046. Abbreviation: ASR, age-standardized rate; DALYs, disability-adjusted life-years.

**Figure S14.** Changes in incidence, prevalence, deaths and DALYs according to aging, population and epidemiological change from 1990 to 2021 at global level by SDI regions. The black dot denotes the overall value of the change resulting from all three components. For each component, the magnitude of a positive value suggests a corresponding increase attributed to the component; the magnitude of a negative value suggests a corresponding decrease attributed to the component. Abbreviations: DALYs, disability-adjusted life-years; SDI, sociodemographic index.

**Figure S15.** Ranking graphs and change patterns for SII and CI of incidence (A) and prevalence (B). Abbreviations: SII, slope index of inequality; GBD, global burden of disease; Δ, the percentage change of inequality from 1990 to 2021; CI, concentration index; SDI, sociodemographic index.

**Table S1.** The case number and ASR of prevalence of MND in 1990 and 2021 for both sex by SDI regions and by GBD regions, with EAPC from 1990 to 2021.

**Table S2.** The case number and ASR of deaths of MND in 1990 and 2021 for both sex by SDI regions and by GBD regions, with EAPC from 1990 to 2021.

**Table S3.** The case number and ASR of DALYs of MND in 1990 and 2021 for both sex by SDI regions and by GBD regions, with EAPC from 1990 to 2021.

**Table S4.** The case number and ASR of incidence of MND in 1990 and 2021, with EAPC from 1990 to 2021.

**Table S5.** The case number and ASR of prevalence of MND in 1990 and 2021, with EAPC from 1990 to 2021.

**Table S6.** The case number and ASR of deaths of MND in 1990 and 2021, with EAPC from 1990 to 2021.

**Table S7.** The case number and ASR of DALYs of MND in 1990 and 2021, with EAPC from 1990 to 2021.

**Table S8.** Changes in incidence of MND according to aging, population and epidemiological change from 1990 to 2021 at global level by SDI regions.

**Table S9.** The predicted case number and ASR of incidence, prevalence, deaths, and DALYs of MND from 2022 to 2035.

**Table S10.** Frontier analysis according to the ASR of incidence, prevalence, deaths, and DALYs and SDI in 2021.


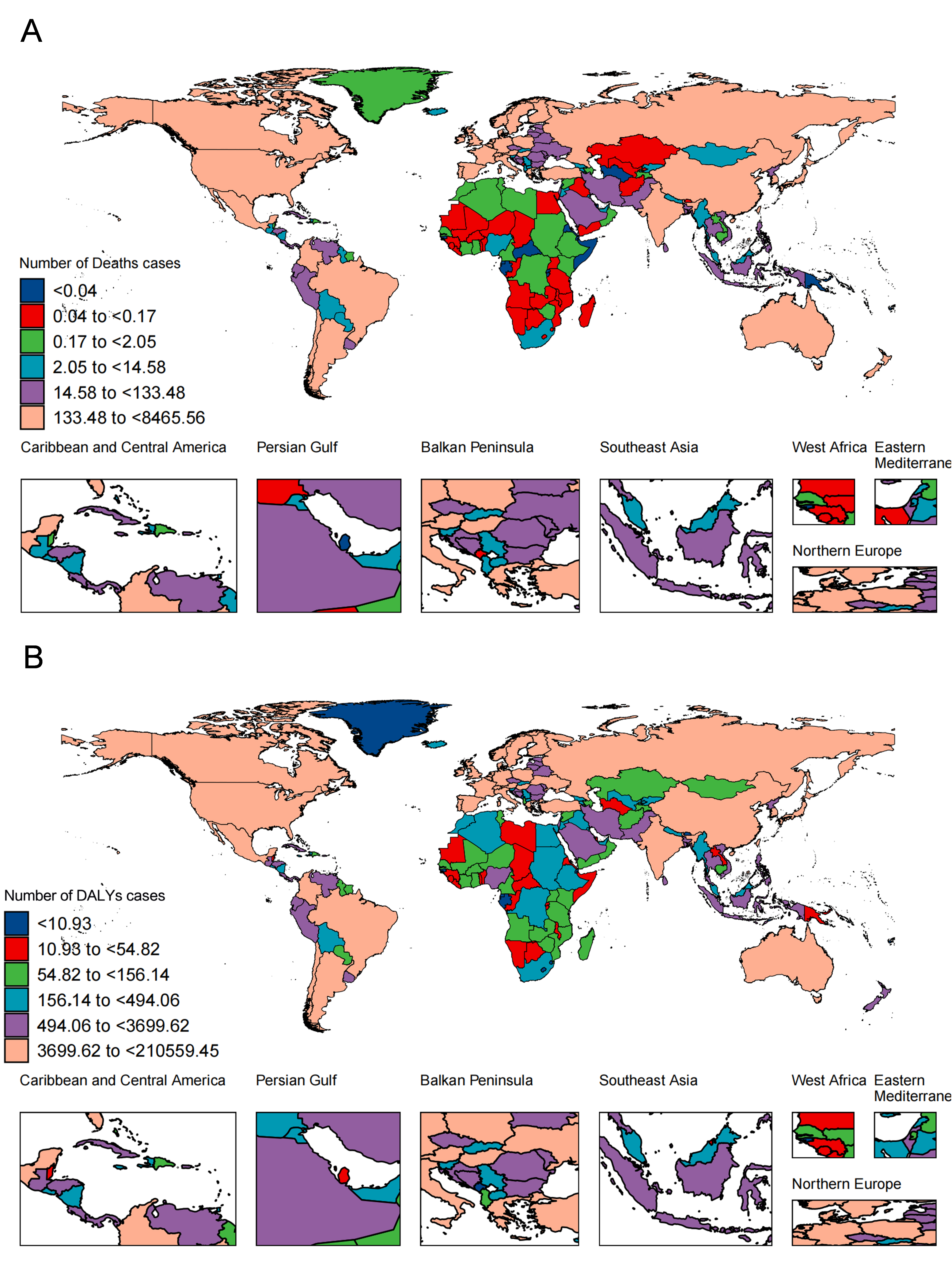


**Figure S1.** The case number of deaths (A) and DALYs (B) in 2021. abbreviations: DALYs, disability-adjusted life-years.


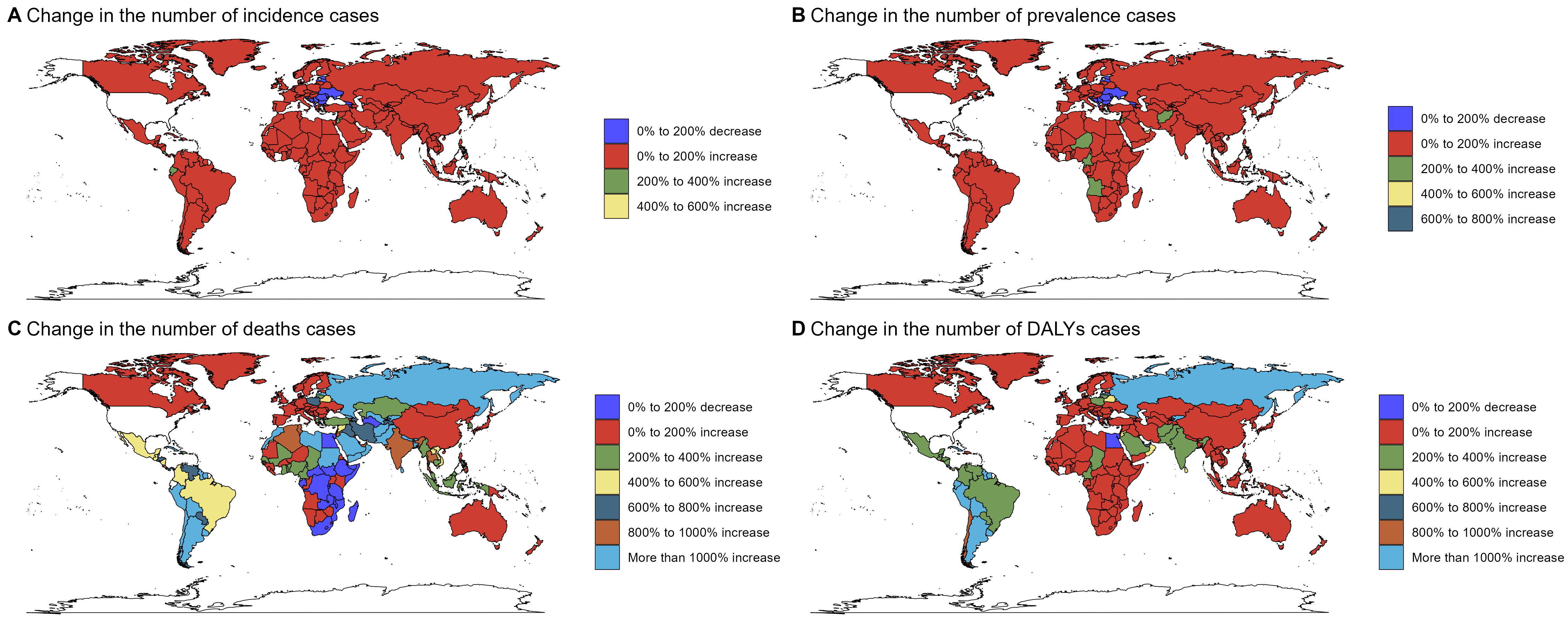


**Figure S2.** The trend in case number of incidence (A), prevalence (B), deaths (C) and DALYs (D) from 1990 to 2021. Abbreviations: DALYs, disability-adjusted life-years.


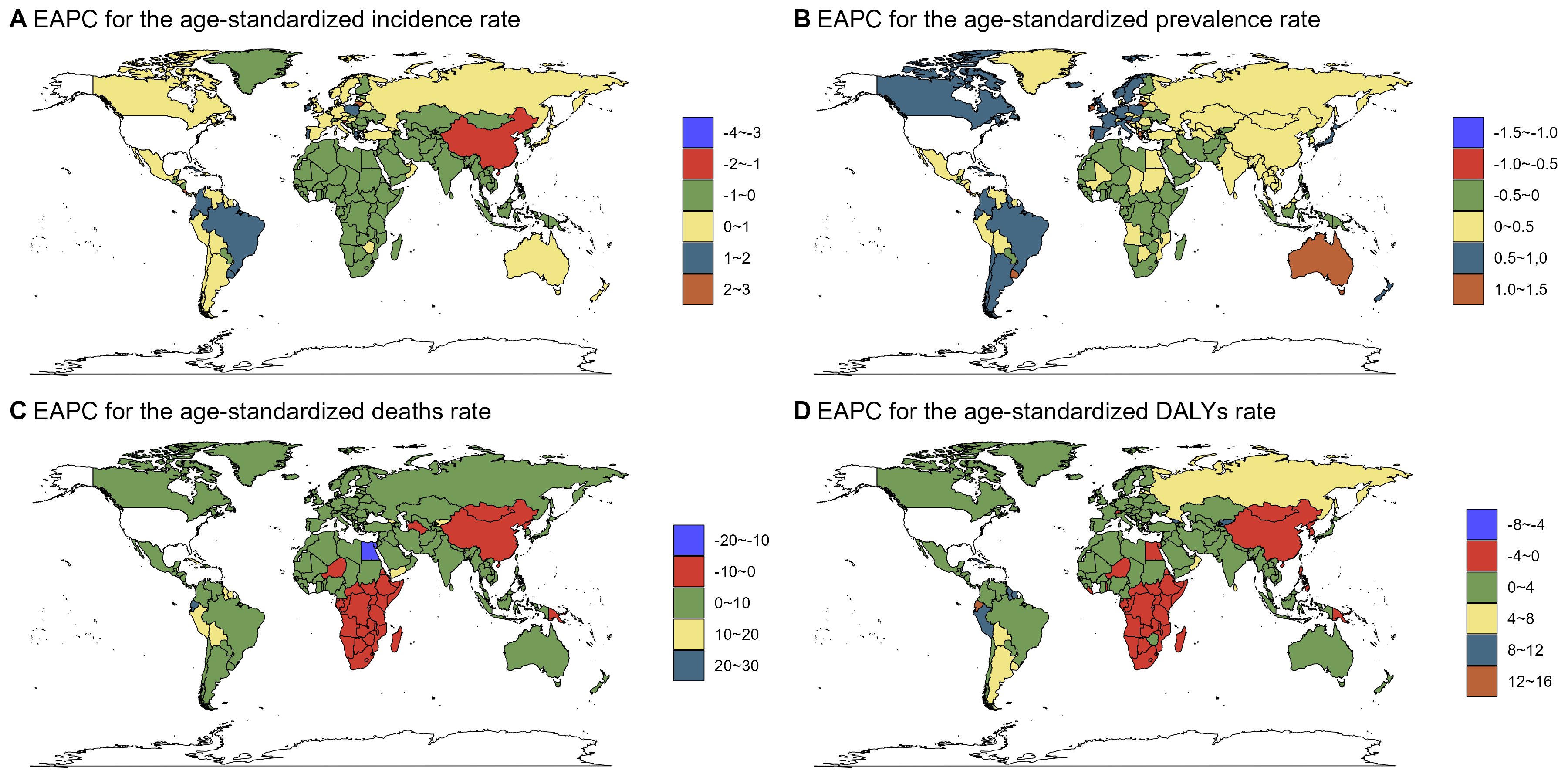


**Figure S3.** (A) The trend in ASR of incidence (EAPC) from 1990 to 2021; (B) The trend in ASR of prevalence (EAPC) from 1990 to 2021; (C) The trend in ASR of deaths (EAPC) from 1990 to 2021; (D) The trend in ASR of DALYs (EAPC) from 1990 to 2021. Abbreviations: ASR, age-standardized rate; EAPC, estimated annual percentage change; DALYs, disability-adjusted life-years.


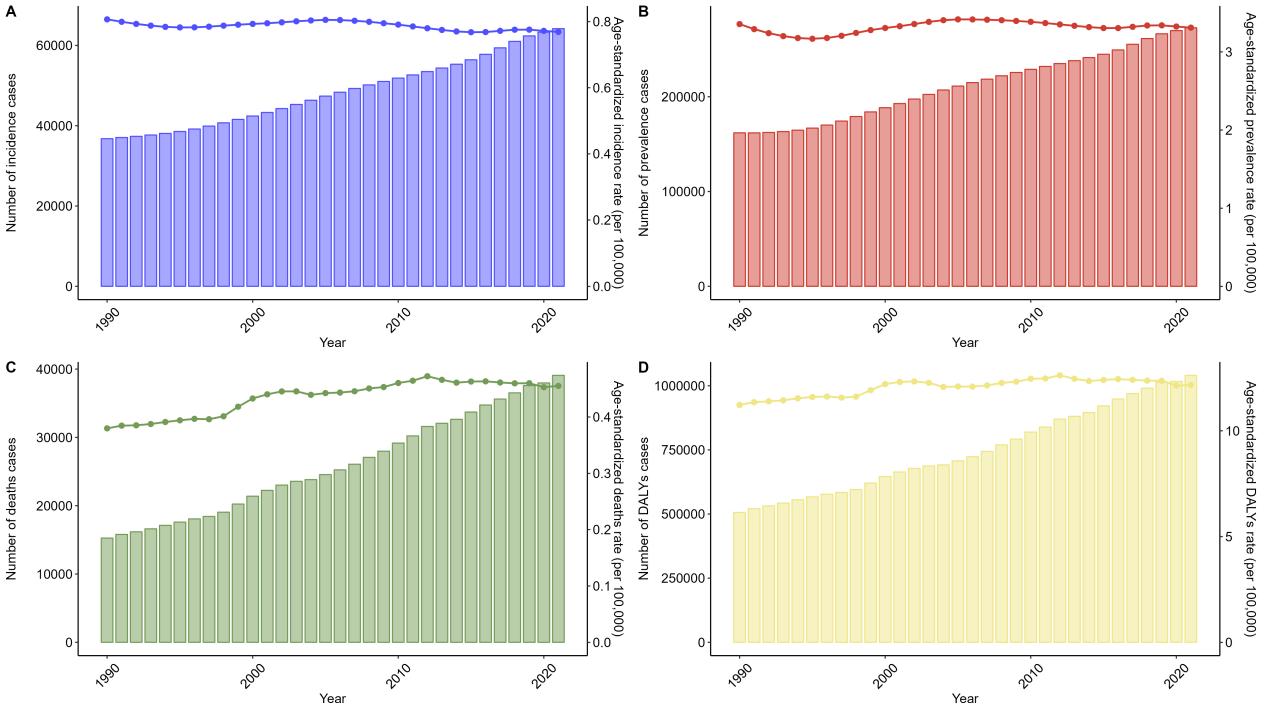


**Figure S4.** The global trend of case number and ASR of incidence (A), prevalence (B), deaths (C) and DALYs (D) of MND from 1990 to 2021. Abbreviations: ASR, age-standardized rate; DALYs, disability-adjusted life-years; MND, motor neuron disease.


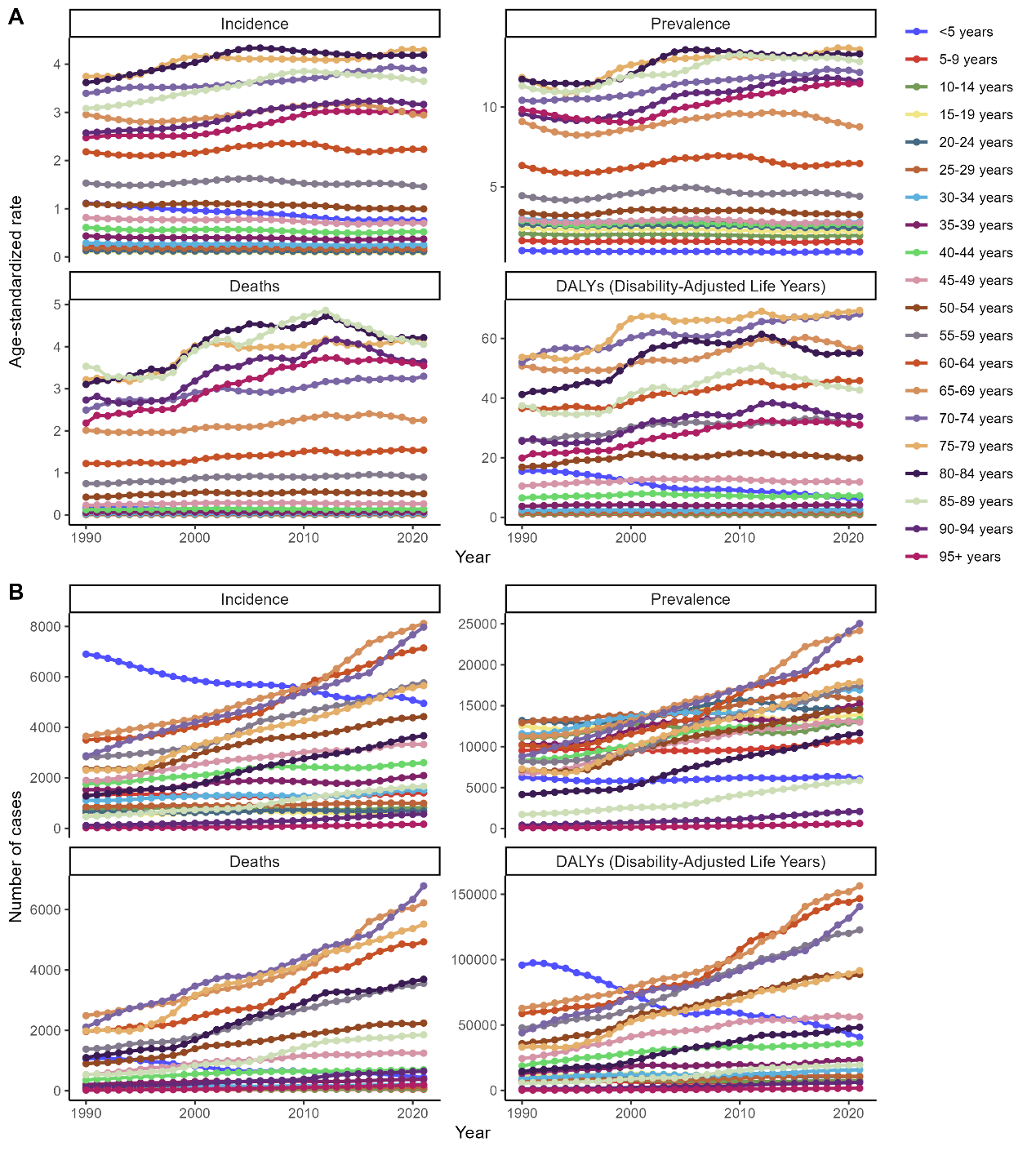


**Figure S5.** The global trend of ASR (A) and case number (B) of incidence, prevalence, deaths, and DALYs by age from 1990 to 2021. Abbreviations: ASR, age-standardized rate; DALYs, disability-adjusted life-years.


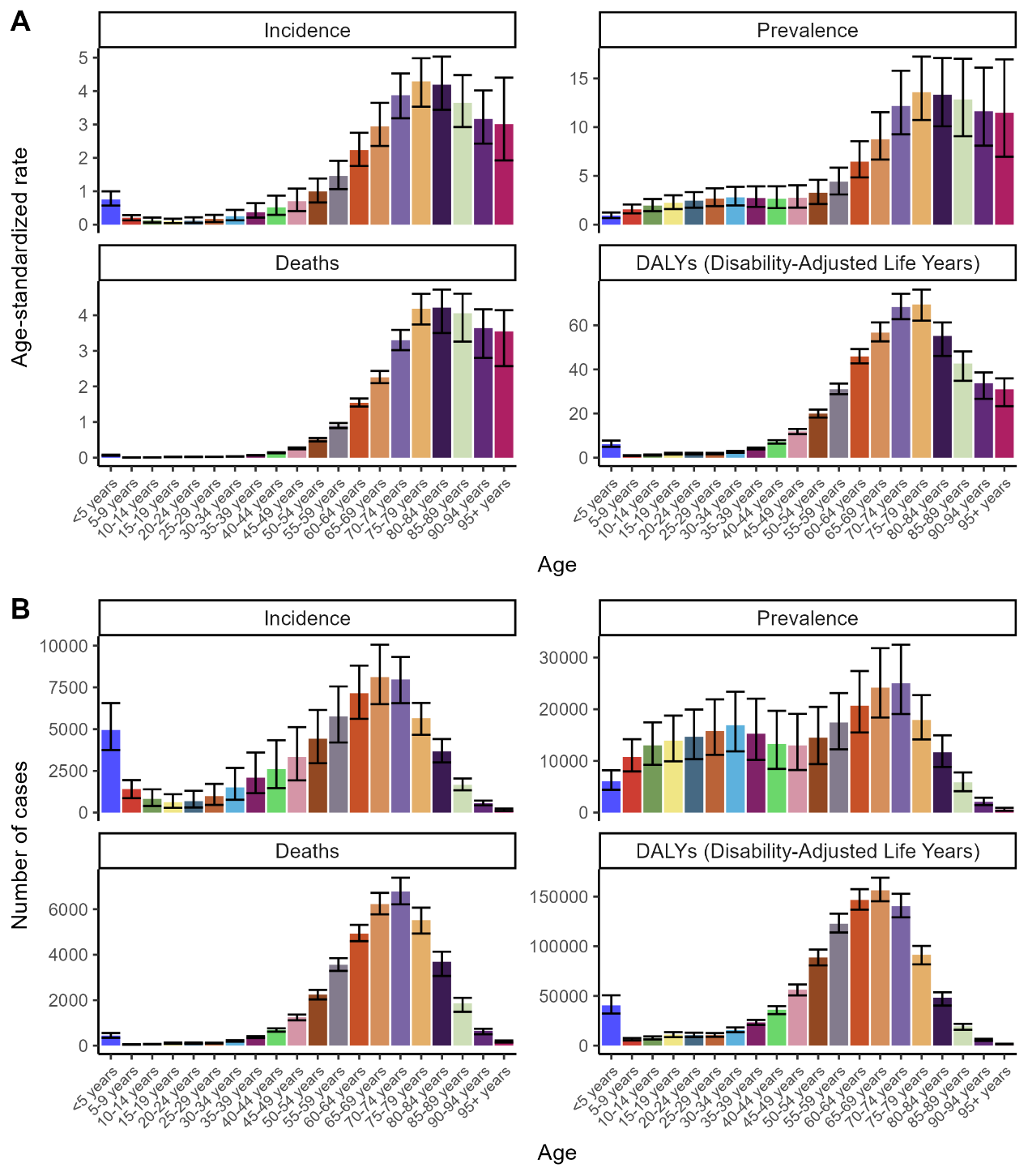
**Figure S6.** The ASR (A) and case number (B) of incidence, prevalence, deaths, and DALYs of MND by age in 2021. Abbreviations: ASR, age-standardized rate; MND, motor neuron disease; DALYs, disability-adjusted life-years.

**
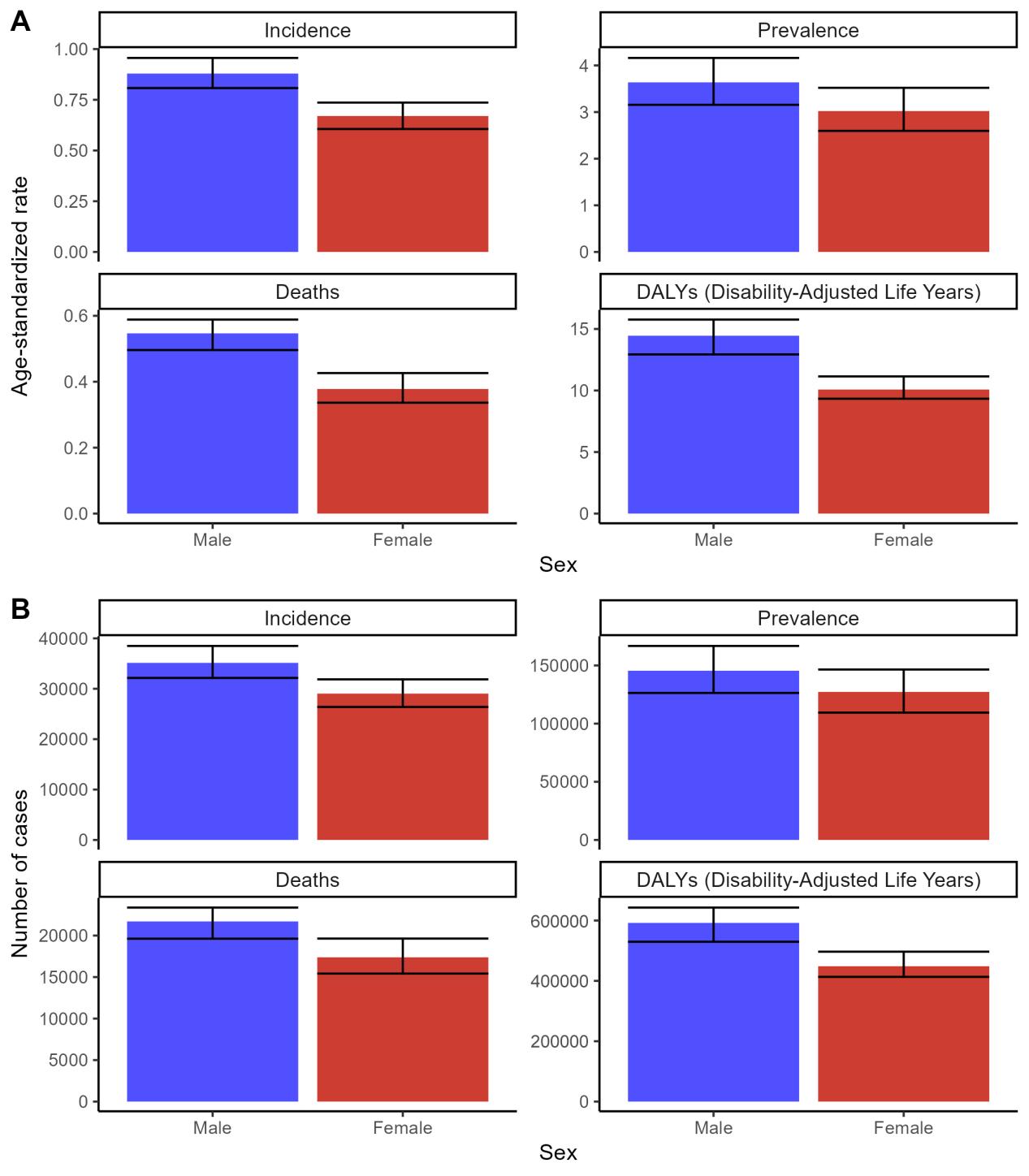
Figure S7.** The ASR (A) and case number (B) of incidence, prevalence, deaths, and DALYs of MND by sex in 2021. Abbreviations：ASR, age-standardized rate; MND, motor neuron disease; DALYs, disability-adjusted life-years.


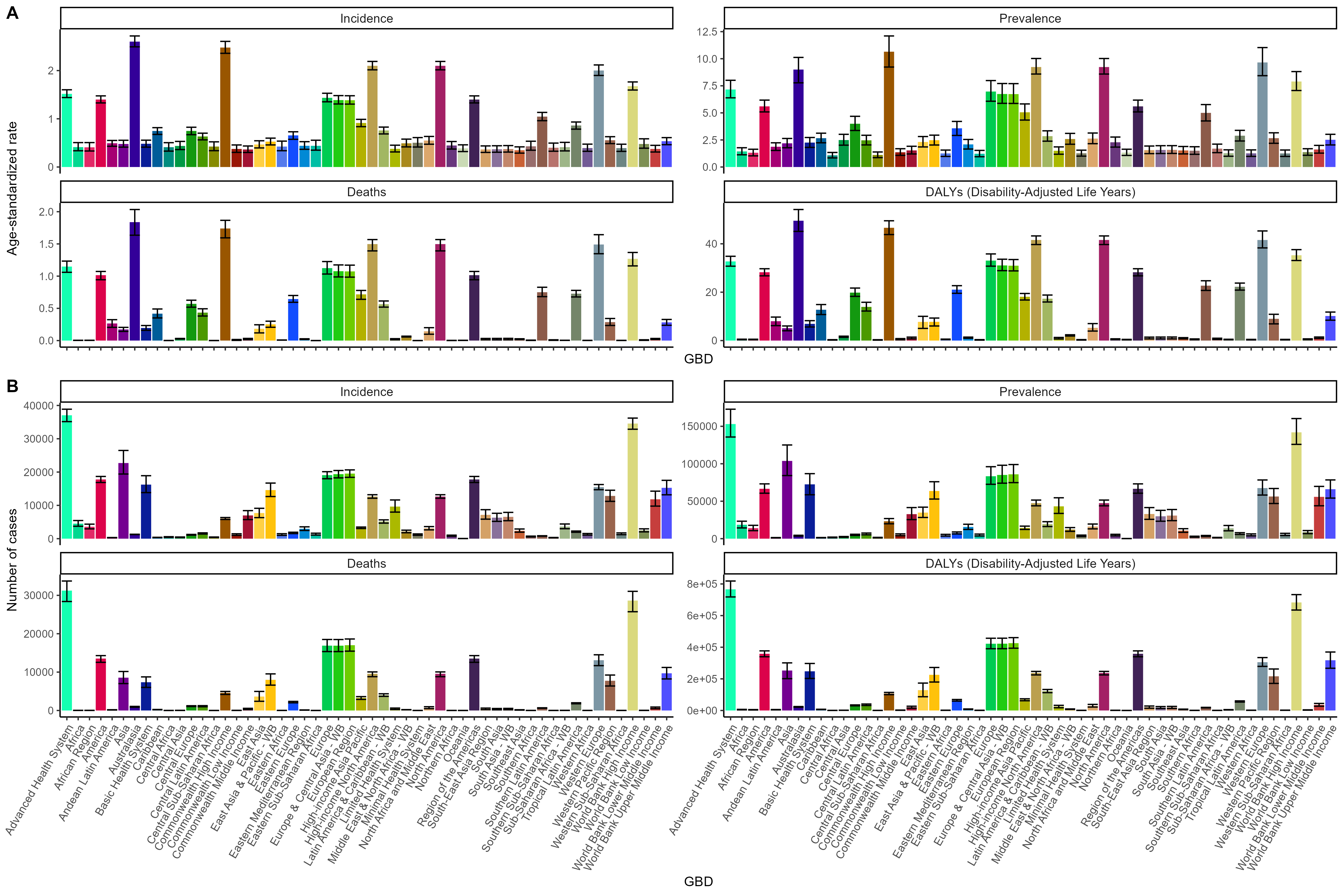
**Figure S8.** The ASR (A) and case number (B) of incidence, prevalence, deaths, and DALYs of MND by GBD regions in 2021. Abbreviations: ASR, age-standardized rate; MND, motor neuron disease; DALYs, disability-adjusted life-years; GBD, Global Burden of Diseases, Injuries, and Risk Factors Study.

**
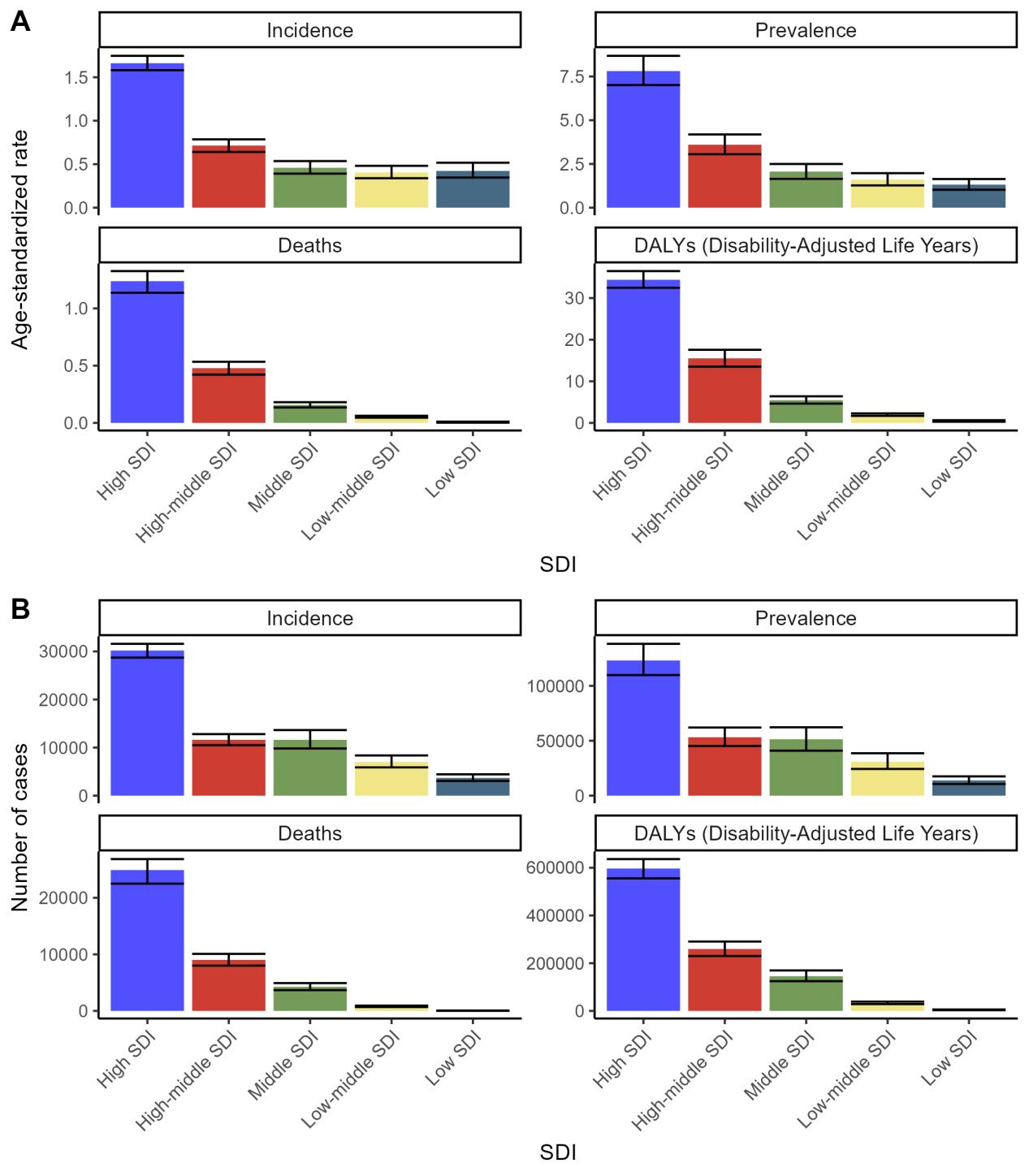
Figure S9.** The ASR (A) and case number (B) of incidence, prevalence, deaths, and DALYs of MND by SDI region in 2021. Abbreviations: ASR, age-standardized rate; MND, motor neuron disease; DALYs, disability-adjusted life-years; SDI, sociodemographic index.

**
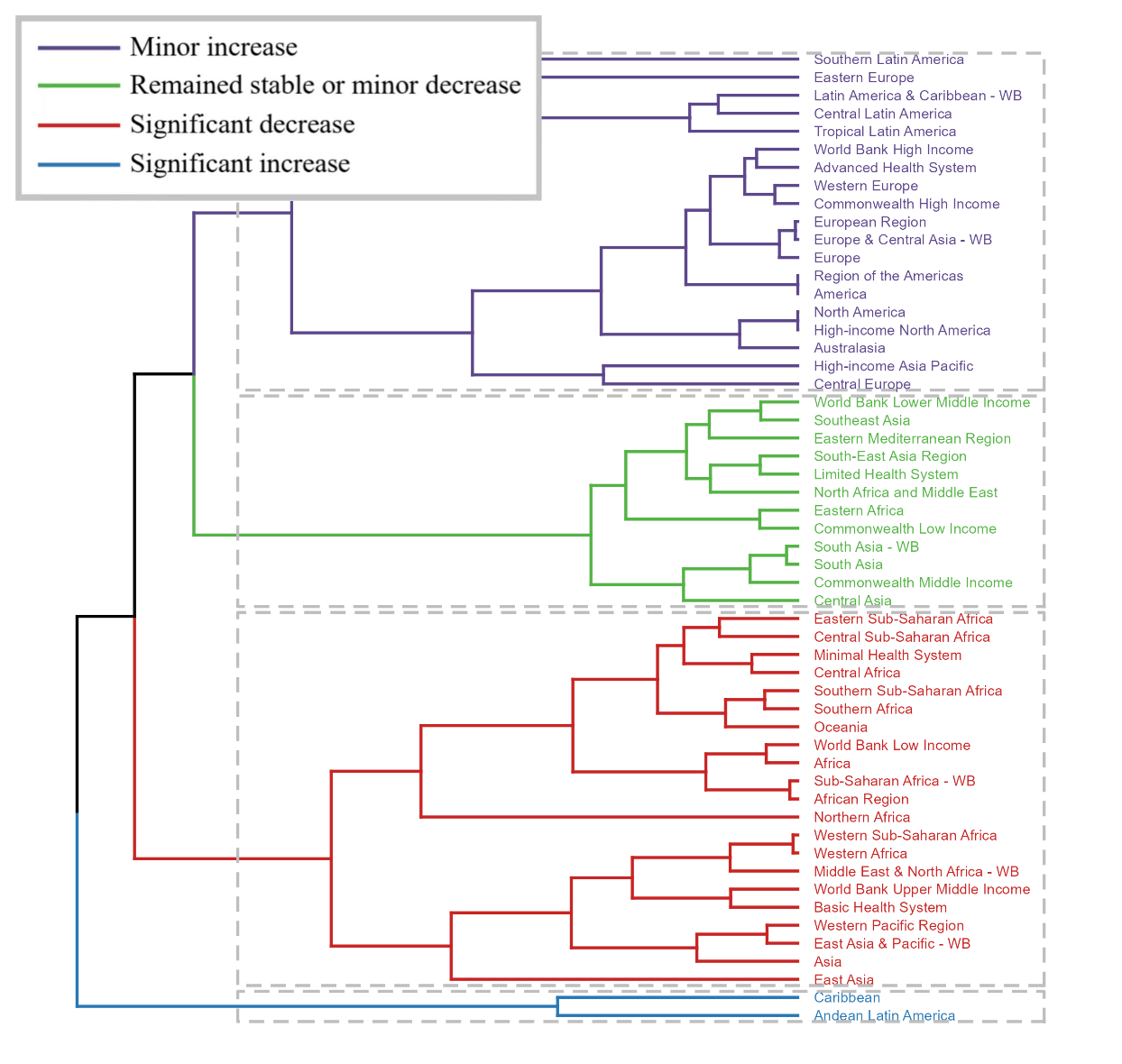
Figure S10.** The clustering of the trend from 1990 to 2021 by GBD regions. Abbreviations: GBD, Global Burden of Diseases.

**F
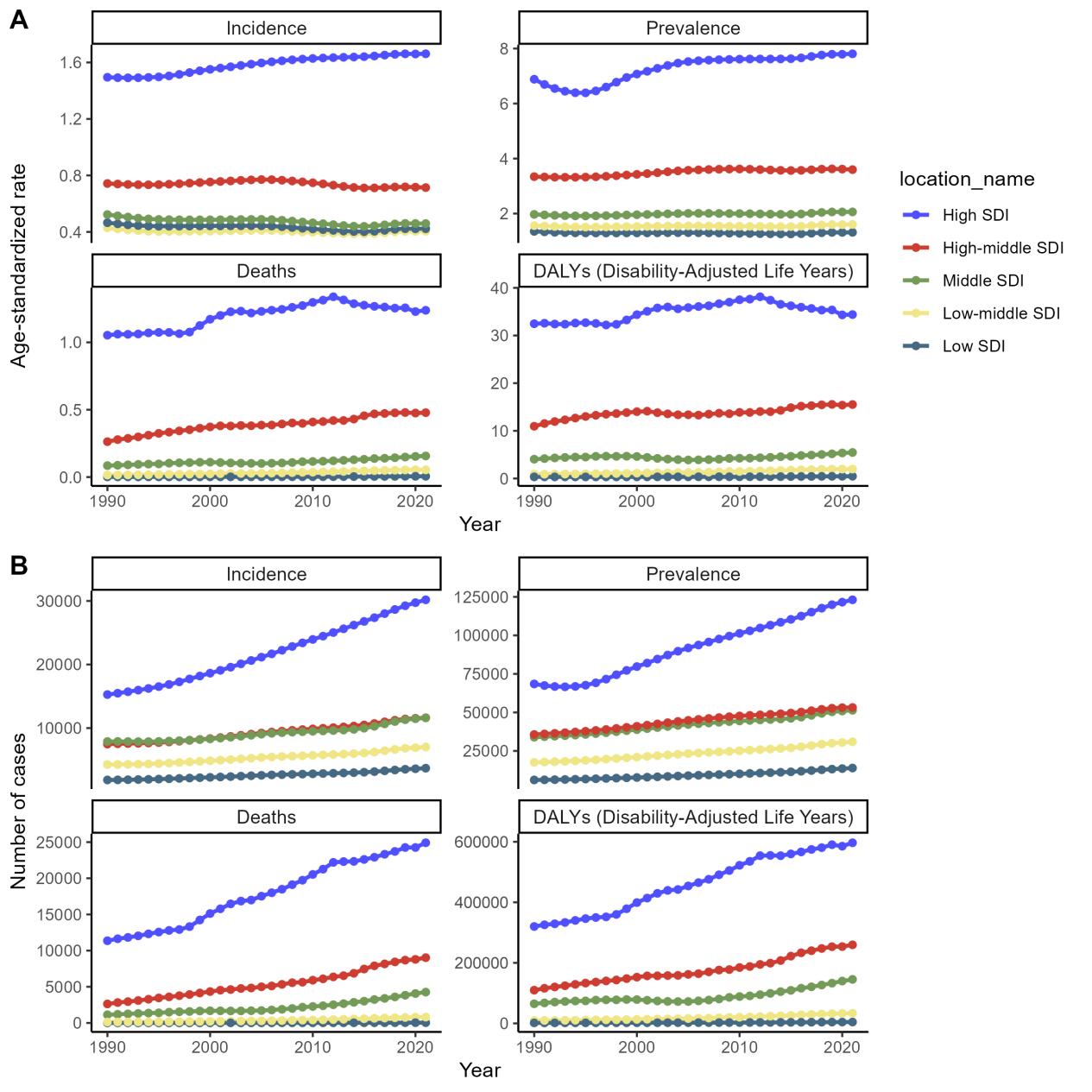
Figure S11.** The trend of ASR (A) and case number (B) of incidence, prevalence, deaths and DALYs of MND by SDI region from 1990 to 2021. Abbreviations: ASR, age-standardized rate; DALYs, disability-adjusted life-years; MND, motor neuron disease; SDI, sociodemographic index.

**
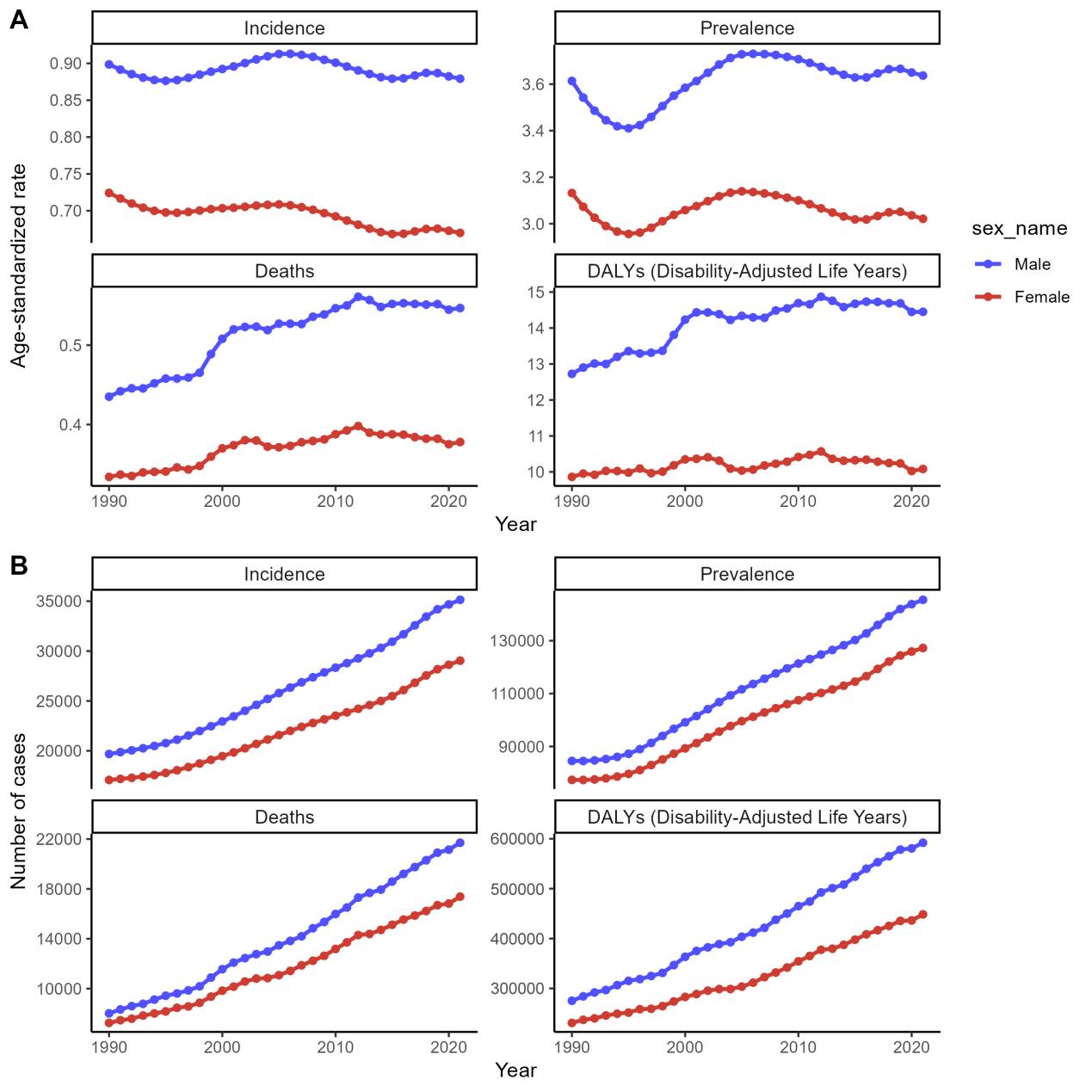
Figure S12.** The trend of ASR (A) and case number (B) of incidence, prevalence, deaths and DALYs of MND by sex from 1990 to 2021. Abbreviations: ASR, age-standardized rate; MND, motor neuron disease; DALYs, disability-adjusted life-years.

**
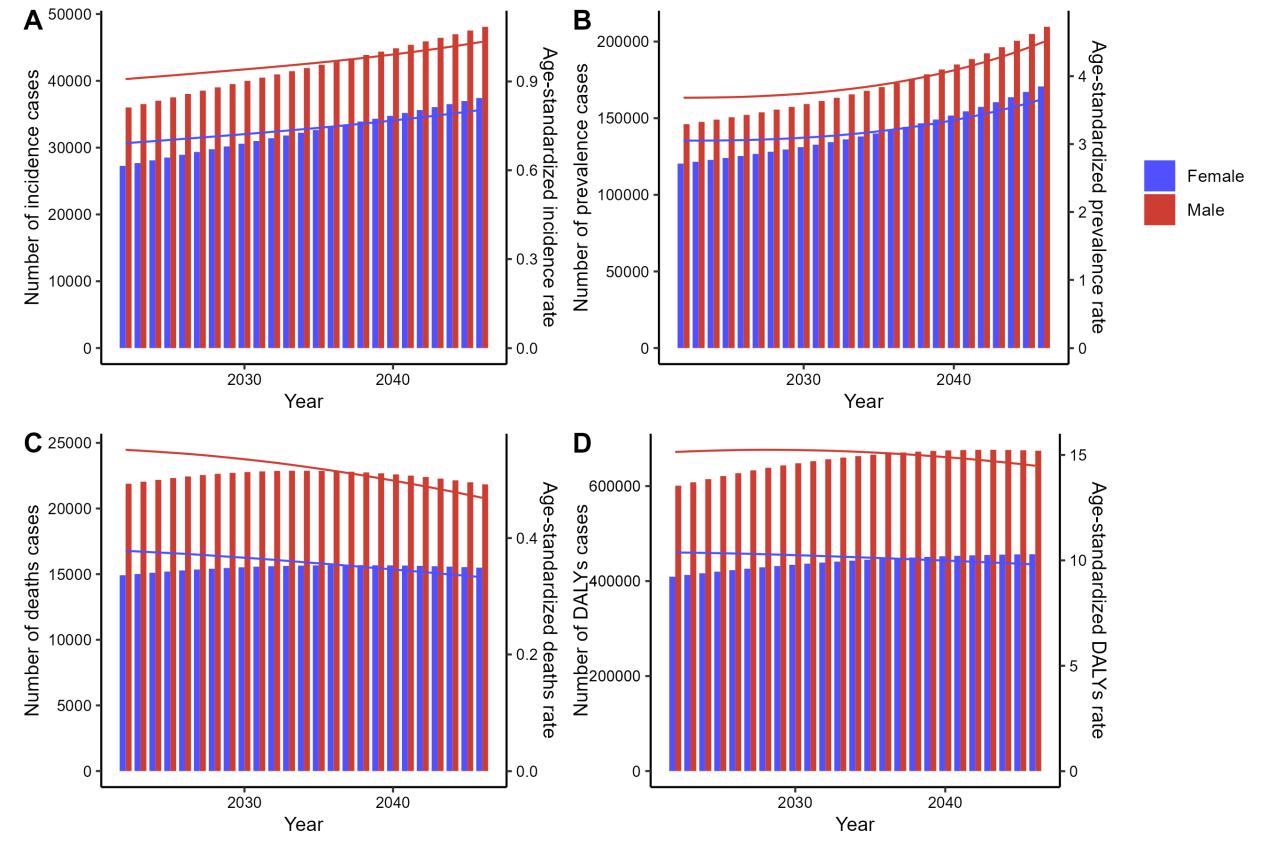
Figure S13.** (A) The predicted case number and ASR of incidence to 2046; (B) The predicted case number and ASR of prevalence to 2046; (C) The predicted case number and ASR of deaths to 2046; (D) The predicted case number and ASR of incidence to 2046. Abbreviation: ASR, age-standardized rate; DALYs, disability-adjusted life-years.

**
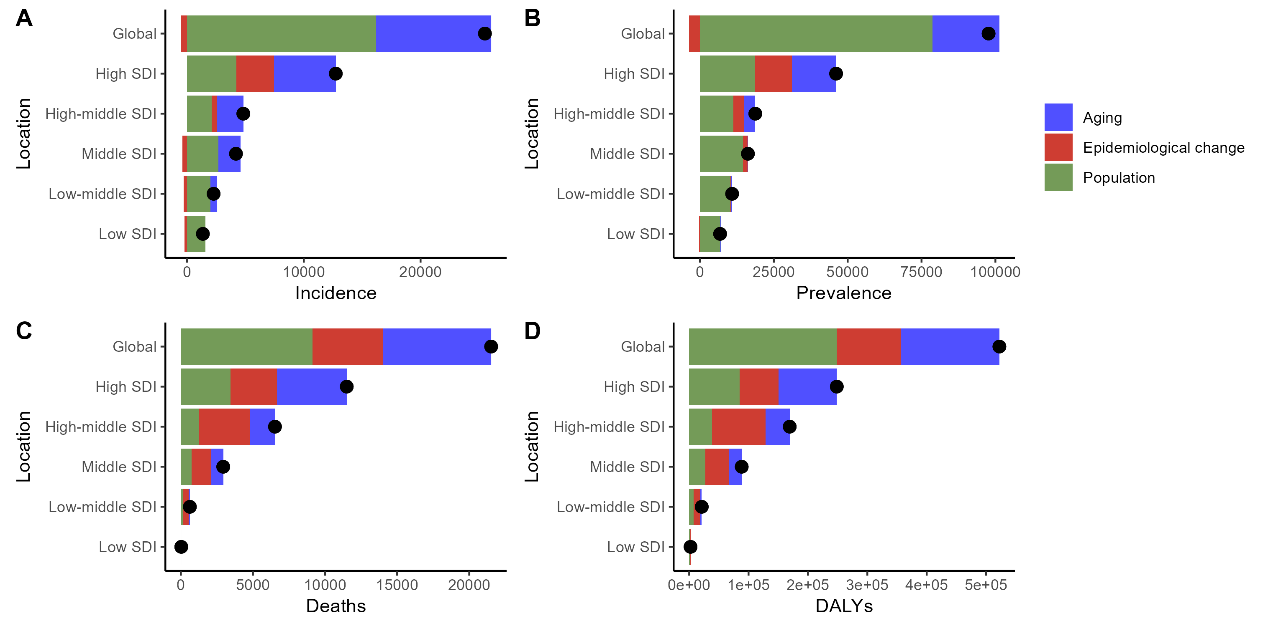
**

**Figure. S14.** Changes in incidence, prevalence, deaths and DALYs according to aging, population and epidemiological change from 1990 to 2021 at global level by SDI regions. The black dot denotes the overall value of the change resulting from all three components. For each component, the magnitude of a positive value suggests a corresponding increase attributed to the component; the magnitude of a negative value suggests a corresponding decrease attributed to the component. Abbreviations: DALYs, disability-adjusted life-years; SDI, sociodemographic index.


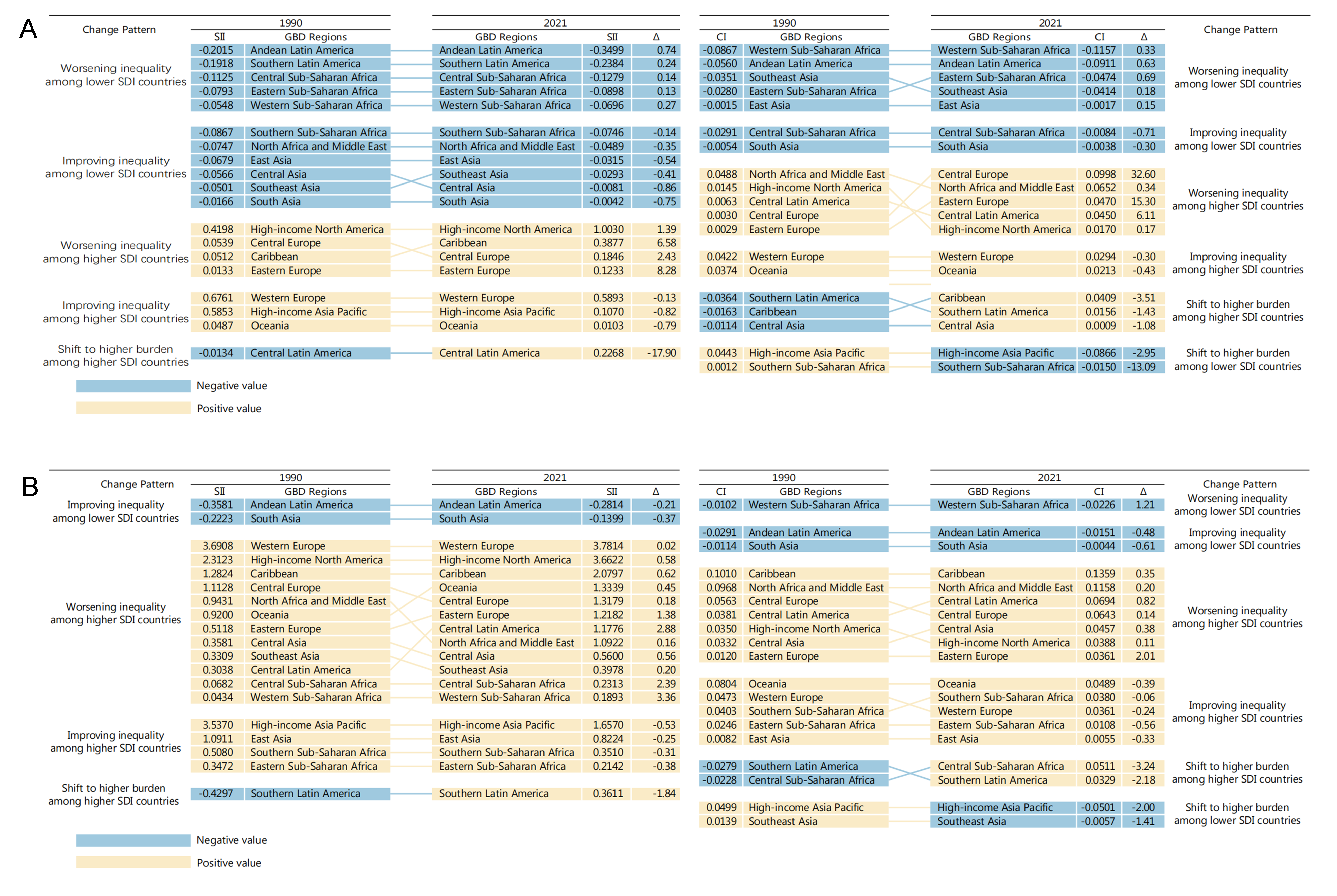


**Figure S15.** Ranking graphs and change patterns for SII and CI of incidence (A) and prevalence (B). Abbreviations: SII, slope index of inequality; GBD, global burden of disease; Δ, the percentage change of inequality from 1990 to 2021; CI, concentration index; SDI, sociodemographic index.

**Table S1.** The case number and ASR of prevalence of MND in 1990 and 2021 for both sex by SDI regions and by GBD regions, with EAPC from 1990 to 2021.

|  | **1990** | |  | **2021** | | **EAPC (95% CI)** |
| --- | --- | --- | --- | --- | --- | --- |
|  | **Number (95% UI)** | **ASR (95% UI)** |  | **Number (95% UI)** | **ASR (95% UI)** |  |
| Global | 161926 (137006 to 189263) | 3.36 (2.87 to 3.92) |  | 272732 (236194 to 313676) | 3.31 (2.86 to 3.80) | 0.11 (0.04 to 0.19) |
| **SDI regions** |  |  |  |  |  |  |
| High-middle SDI | 35651 (29922 to 41940) | 3.34 (2.83 to 3.91) |  | 53188 (45204 to 62047) | 3.60 (3.05 to 4.19) | 0.33 (0.27 to 0.38) |
| High SDI | 68474 (59429 to 78627) | 6.88 (6.01 to 7.86) |  | 123101 (109797 to 138256) | 7.81 (7.01 to 8.68) | 0.67 (0.55 to 0.78) |
| Low-middle SDI | 17573 (13572 to 21999) | 1.56 (1.23 to 1.93) |  | 30937 (24326 to 38615) | 1.60 (1.27 to 1.97) | 0.11 (0.06 to 0.15) |
| Low SDI | 6158 (4723 to 7762) | 1.35 (1.05 to 1.68) |  | 13898 (10612 to 17565) | 1.32 (1.03 to 1.64) | −0.07 (−0.13 to −0.02) |
| Middle SDI | 33916 (26744 to 41892) | 1.97 (1.58 to 2.40) |  | 51387 (40966 to 62241) | 2.06 (1.65 to 2.50) | 0.18 (0.14 to 0.23) |
| **GBD regions** |  |  |  |  |  |  |
| Advanced Health System | 88647 (77125 to 101986) | 6.05 (5.28 to 6.92) |  | 153041 (135695 to 172827) | 7.16 (6.39 to 8.01) | 0.78 (0.68 to 0.88) |
| Africa | 8835 (6897 to 10992) | 1.54 (1.20 to 1.89) |  | 18601 (14361 to 23312) | 1.44 (1.13 to 1.78) | −0.19 (−0.24 to −0.15) |
| African Region | 6374 (4894 to 7997) | 1.40 (1.09 to 1.73) |  | 14068 (10672 to 17826) | 1.32 (1.03 to 1.64) | −0.17 (−0.22 to −0.12) |
| America | 36074 (31425 to 41339) | 5.39 (4.68 to 6.18) |  | 66592 (60691 to 73159) | 5.60 (5.09 to 6.18) | 0.62 (0.45 to 0.80) |
| Andean Latin America | 605 (479 to 749) | 1.70 (1.37 to 2.07) |  | 1229 (999 to 1476) | 1.87 (1.53 to 2.24) | 0.42 (0.38 to 0.45) |
| Asia | 64900 (52069 to 79427) | 2.13 (1.74 to 2.56) |  | 103790 (84254 to 125082) | 2.19 (1.78 to 2.64) | 0.10 (0.04 to 0.16) |
| Australasia | 1520 (1315 to 1744) | 6.83 (5.95 to 7.80) |  | 3933 (3379 to 4536) | 9.00 (7.78 to 10.12) | 1.00 (0.91 to 1.08) |
| Basic Health System | 49356 (39531 to 60605) | 2.14 (1.74 to 2.58) |  | 72524 (58637 to 86781) | 2.26 (1.81 to 2.73) | 0.21 (0.16 to 0.25) |
| Caribbean | 863 (691 to 1041) | 2.49 (2.02 to 2.97) |  | 1306 (1100 to 1527) | 2.66 (2.24 to 3.12) | 0.31 (0.29 to 0.34) |
| Central Africa | 665 (511 to 842) | 1.14 (0.88 to 1.42) |  | 1644 (1251 to 2104) | 1.08 (0.84 to 1.34) | −0.17 (−0.22 to −0.13) |
| Central Asia | 1756 (1408 to 2136) | 2.56 (2.07 to 3.09) |  | 2375 (1902 to 2883) | 2.49 (2.02 to 3.02) | −0.02 (−0.09 to 0.05) |
| Central Europe | 4512 (3777 to 5319) | 3.56 (2.97 to 4.18) |  | 5063 (4299 to 5906) | 4.00 (3.37 to 4.68) | 0.47 (0.43 to 0.51) |
| Central Latin America | 3604 (2823 to 4427) | 2.25 (1.80 to 2.72) |  | 6312 (5168 to 7525) | 2.46 (2.04 to 2.93) | 0.39 (0.35 to 0.42) |
| Central Sub-Saharan Africa | 558 (428 to 705) | 1.17 (0.91 to 1.45) |  | 1381 (1047 to 1762) | 1.11 (0.87 to 1.39) | −0.14 (−0.19 to −0.09) |
| Commonwealth High Income | 11568 (10136 to 13188) | 8.75 (7.66 to 9.93) |  | 23231 (19879 to 26858) | 10.65 (9.23 to 12.1) | 0.73 (0.66 to 0.79) |
| Commonwealth Low Income | 2631 (2043 to 3325) | 1.38 (1.09 to 1.70) |  | 5121 (3948 to 6422) | 1.36 (1.08 to 1.69) | −0.05 (−0.09 to −0.01) |
| Commonwealth Middle Income | 17157 (13041 to 21809) | 1.49 (1.15 to 1.86) |  | 32754 (25422 to 41517) | 1.53 (1.21 to 1.92) | 0.11 (0.05 to 0.17) |
| East Asia | 26778 (21282 to 33189) | 2.15 (1.74 to 2.62) |  | 34809 (28296 to 42072) | 2.31 (1.85 to 2.81) | 0.27 (0.20 to 0.33) |
| East Asia & Pacific - WB | 43482 (35370 to 52598) | 2.39 (1.98 to 2.86) |  | 63685 (52882 to 76010) | 2.48 (2.03 to 2.94) | 0.14 (0.06 to 0.22) |
| Eastern Africa | 2009 (1528 to 2536) | 1.30 (1.00 to 1.61) |  | 4537 (3426 to 5779) | 1.26 (0.98 to 1.56) | −0.11 (−0.16 to −0.05) |
| Eastern Europe | 7713 (6201 to 9319) | 3.44 (2.76 to 4.12) |  | 7588 (6309 to 8978) | 3.58 (2.91 to 4.21) | 0.27 (0.18 to 0.35) |
| Eastern Mediterranean Region | 7767 (6164 to 9541) | 2.15 (1.71 to 2.61) |  | 15677 (12294 to 19250) | 2.09 (1.66 to 2.54) | −0.07 (−0.10 to −0.03) |
| Eastern Sub-Saharan Africa | 2106 (1595 to 2675) | 1.27 (0.98 to 1.58) |  | 4807 (3618 to 6142) | 1.23 (0.95 to 1.53) | −0.11 (−0.17 to −0.06) |
| Europe | 51837 (45108 to 59778) | 5.74 (4.98 to 6.61) |  | 83362 (72591 to 96071) | 6.97 (6.07 to 7.98) | 0.73 (0.67 to 0.78) |
| Europe & Central Asia - WB | 53023 (46155 to 61140) | 5.62 (4.87 to 6.47) |  | 85088 (74082 to 98009) | 6.74 (5.88 to 7.71) | 0.69 (0.64 to 0.75) |
| European Region | 53397 (46481 to 61568) | 5.62 (4.87 to 6.46) |  | 85920 (74822 to 98960) | 6.73 (5.87 to 7.70) | 0.69 (0.63 to 0.74) |
| High-income Asia Pacific | 8352 (7038 to 9721) | 4.45 (3.75 to 5.16) |  | 14361 (12291 to 16740) | 5.05 (4.33 to 5.83) | 0.43 (0.33 to 0.53) |
| High-income North America | 25480 (22210 to 29153) | 8.13 (7.10 to 9.25) |  | 47431 (43862 to 51537) | 9.23 (8.59 to 10.02) | 1.07 (0.83 to 1.32) |
| Latin America & Caribbean - WB | 10715 (8516 to 12955) | 2.55 (2.07 to 3.05) |  | 19324 (16183 to 22655) | 2.85 (2.39 to 3.34) | 0.46 (0.43 to 0.48) |
| Limited Health System | 22245 (17018 to 28069) | 1.47 (1.14 to 1.83) |  | 43149 (33700 to 54596) | 1.50 (1.18 to 1.86) | 0.07 (0.02 to 0.13) |
| Middle East & North Africa - WB | 6373 (5091 to 7760) | 2.63 (2.13 to 3.14) |  | 12217 (9770 to 14803) | 2.58 (2.10 to 3.10) | −0.01 (−0.05 to 0.02) |
| Minimal Health System | 1523 (1197 to 1904) | 1.30 (1.02 to 1.59) |  | 3797 (2937 to 4770) | 1.27 (1.00 to 1.56) | −0.07 (−0.11 to −0.04) |
| North Africa and Middle East | 8542 (6909 to 10299) | 2.64 (2.16 to 3.15) |  | 16230 (13099 to 19540) | 2.63 (2.15 to 3.14) | 0.04 (0.01 to 0.07) |
| North America | 25478 (22208 to 29151) | 8.13 (7.10 to 9.25) |  | 47428 (43860 to 51534) | 9.23 (8.58 to 10.02) | 1.07 (0.83 to 1.32) |
| Northern Africa | 2721 (2171 to 3336) | 2.31 (1.88 to 2.80) |  | 4790 (3817 to 5835) | 2.28 (1.84 to 2.77) | −0.02 (−0.06 to 0.01) |
| Oceania | 91 (71 to 114) | 1.44 (1.16 to 1.75) |  | 180 (140 to 225) | 1.33 (1.07 to 1.64) | −0.30 (−0.35 to −0.25) |
| Region of the Americas | 36074 (31425 to 41339) | 5.39 (4.68 to 6.18) |  | 66592 (60691 to 73159) | 5.60 (5.09 to 6.18) | 0.62 (0.45 to 0.80) |
| South-East Asia Region | 19194 (14669 to 24349) | 1.49 (1.16 to 1.85) |  | 32960 (25804 to 41514) | 1.56 (1.24 to 1.94) | 0.15 (0.10 to 0.21) |
| South Asia | 16034 (12216 to 20377) | 1.49 (1.15 to 1.86) |  | 29896 (23274 to 37806) | 1.58 (1.24 to 1.97) | 0.20 (0.14 to 0.26) |
| South Asia - WB | 16494 (12575 to 20949) | 1.49 (1.16 to 1.87) |  | 30841 (24062 to 38920) | 1.58 (1.25 to 1.97) | 0.20 (0.14 to 0.26) |
| Southeast Asia | 7013 (5448 to 8777) | 1.51 (1.20 to 1.87) |  | 10765 (8471 to 13331) | 1.53 (1.21 to 1.89) | 0.01 (−0.04 to 0.06) |
| Southern Africa | 1393 (1077 to 1746) | 1.61 (1.26 to 1.99) |  | 2611 (2010 to 3286) | 1.50 (1.17 to 1.87) | −0.20 (−0.25 to −0.15) |
| Southern Latin America | 1889 (1575 to 2219) | 3.86 (3.23 to 4.53) |  | 3707 (3158 to 4277) | 5.00 (4.25 to 5.77) | 0.90 (0.85 to 0.95) |
| Southern Sub-Saharan Africa | 903 (694 to 1131) | 1.78 (1.39 to 2.21) |  | 1368 (1062 to 1729) | 1.70 (1.33 to 2.11) | −0.14 (−0.20 to −0.08) |
| Sub-Saharan Africa - WB | 6137 (4674 to 7733) | 1.34 (1.04 to 1.67) |  | 13858 (10484 to 17609) | 1.28 (0.99 to 1.59) | −0.16 (−0.21 to −0.11) |
| Tropical Latin America | 3787 (2999 to 4644) | 2.53 (2.03 to 3.04) |  | 6820 (5688 to 7990) | 2.89 (2.40 to 3.39) | 0.56 (0.53 to 0.59) |
| Western Africa | 2046 (1554 to 2596) | 1.32 (1.01 to 1.64) |  | 5019 (3779 to 6387) | 1.26 (0.98 to 1.58) | −0.10 (−0.15 to −0.05) |
| Western Europe | 37572 (32710 to 43082) | 7.74 (6.73 to 8.88) |  | 67563 (58235 to 78365) | 9.66 (8.43 to 11.03) | 0.77 (0.70 to 0.84) |
| Western Pacific Region | 38270 (31250 to 46178) | 2.53 (2.11 to 3.03) |  | 56289 (46938 to 66980) | 2.66 (2.19 to 3.16) | 0.20 (0.12 to 0.28) |
| Western Sub-Saharan Africa | 2251 (1712 to 2851) | 1.31 (1.01 to 1.63) |  | 5611 (4227 to 7139) | 1.26 (0.98 to 1.57) | −0.10 (−0.15 to −0.05) |
| World Bank High Income | 78739 (68313 to 90435) | 6.71 (5.87 to 7.66) |  | 141970 (125872 to 160343) | 7.90 (7.06 to 8.81) | 0.76 (0.65 to 0.87) |
| World Bank Low Income | 4046 (3199 to 5006) | 1.48 (1.17 to 1.81) |  | 8656 (6714 to 10846) | 1.38 (1.08 to 1.69) | −0.21 (−0.25 to −0.17) |
| World Bank Lower Middle Income | 31506 (24269 to 39462) | 1.62 (1.27 to 2.01) |  | 55840 (43925 to 69782) | 1.62 (1.28 to 2.00) | 0.01 (−0.05 to 0.06) |
| World Bank Upper Middle Income | 47479 (38425 to 57689) | 2.37 (1.93 to 2.83) |  | 66045 (54179 to 78429) | 2.52 (2.04 to 3.02) | 0.27 (0.23 to 0.32) |

Abbreviations: ASR, age-standardized rate; MND, motor neuron diseases; EAPC, estimated annual percentage change; SDI, sociodemographic index; GBD, Global Burden of Diseases, Injuries, and Risk Factors Study; UI, uncertainty interval; CI, confidence interval.

**Table S2.** The case number and ASR of deaths of MND in 1990 and 2021 for both sex by SDI regions and by GBD regions, with EAPC from 1990 to 2021.

|  | **1990** | |  | **2021** | | **EAPC (95% CI)** |
| --- | --- | --- | --- | --- | --- | --- |
|  | **Number (95% UI)** | **ASR (95% UI)** |  | **Number (95% UI)** | **ASR (95% UI)** |  |
| Global | 15260 (14367 to 16043) | 0.38 (0.36 to 0.40) |  | 39082 (35757 to 42433) | 0.46 (0.42 to 0.49) | 0.69 (0.57 to 0.80) |
| **SDI regions** |  |  |  |  |  |  |
| High-middle SDI | 2626 (2220 to 3025) | 0.26 (0.22 to 0.30) |  | 9023 (8008 to 10086) | 0.48 (0.42 to 0.53) | 1.73 (1.58 to 1.89) |
| High SDI | 11365 (10853 to 11632) | 1.05 (1.01 to 1.08) |  | 24911 (22501 to 26848) | 1.24 (1.14 to 1.33) | 0.73 (0.57 to 0.88) |
| Low-middle SDI | 121 (100 to 154) | 0.02 (0.01 to 0.02) |  | 809 (686 to 940) | 0.05 (0.05 to 0.06) | 4.37 (4.28 to 4.46) |
| Low SDI | 5 (3 to 8) | 0 (0 to 0) |  | 36 (14 to 65) | 0.01 (0 to 0.01) | 5.07 (4.66 to 5.47) |
| Middle SDI | 1133 (811 to 1332) | 0.09 (0.06 to 0.10) |  | 4266 (3675 to 4918) | 0.16 (0.13 to 0.18) | 1.63 (1.41 to 1.86) |
| **GBD regions** |  |  |  |  |  |  |
| Advanced Health System | 13088 (12493 to 13471) | 0.82 (0.79 to 0.85) |  | 31237 (28392 to 33733) | 1.15 (1.06 to 1.23) | 1.28 (1.14 to 1.42) |
| Africa | 14 (8 to 17) | 0 (0 to 0) |  | 29 (23 to 36) | 0 (0 to 0) | −0.56 (−1.46 to 0.35) |
| African Region | 9 (5 to 13) | 0 (0 to 0) |  | 27 (22 to 34) | 0 (0 to 0.01) | 0.66 (−0.06 to 1.38) |
| America | 4865 (4635 to 4998) | 0.78 (0.74 to 0.80) |  | 13530 (12547 to 14297) | 1.02 (0.94 to 1.07) | 0.92 (0.65 to 1.20) |
| Andean Latin America | 1 (1 to 2) | 0 (0 to 0.01) |  | 160 (123 to 196) | 0.27 (0.21 to 0.32) | 13.85 (11.27 to 16.50) |
| Asia | 3168 (2410 to 3665) | 0.14 (0.11 to 0.16) |  | 8553 (7010 to 10160) | 0.17 (0.14 to 0.20) | 0.34 (0.16 to 0.53) |
| Australasia | 375 (354 to 391) | 1.60 (1.52 to 1.67) |  | 960 (846 to 1068) | 1.84 (1.64 to 2.03) | 0.60 (0.31 to 0.89) |
| Basic Health System | 2102 (1399 to 2540) | 0.11 (0.08 to 0.14) |  | 7363 (6015 to 8753) | 0.20 (0.16 to 0.23) | 1.28 (1.02 to 1.54) |
| Caribbean | 3 (3 to 4) | 0.01 (0.01 to 0.01) |  | 223 (188 to 262) | 0.42 (0.35 to 0.49) | 10.45 (7.19 to 13.80) |
| Central Africa | 1 (0 to 1) | 0 (0 to 0) |  | 1 (1 to 2) | 0 (0 to 0) | −1.85 (−2.70 to −0.99) |
| Central Asia | 3 (3 to 5) | 0.01 (0 to 0.01) |  | 26 (23 to 30) | 0.03 (0.02 to 0.03) | 6.55 (5.68 to 7.43) |
| Central Europe | 310 (299 to 325) | 0.23 (0.22 to 0.24) |  | 1125 (1026 to 1238) | 0.57 (0.52 to 0.63) | 3.38 (3.10 to 3.67) |
| Central Latin America | 186 (181 to 190) | 0.19 (0.18 to 0.19) |  | 1109 (973 to 1261) | 0.43 (0.38 to 0.49) | 2.81 (2.67 to 2.95) |
| Central Sub-Saharan Africa | 1 (0 to 1) | 0 (0 to 0) |  | 0 (0 to 1) | 0 (0 to 0) | −3.96 (−5.11 to −2.80) |
| Commonwealth High Income | 2148 (2071 to 2191) | 1.45 (1.41 to 1.48) |  | 4605 (4181 to 4959) | 1.74 (1.59 to 1.87) | 0.85 (0.56 to 1.15) |
| Commonwealth Low Income | 2 (1 to 4) | 0 (0 to 0) |  | 21 (7 to 41) | 0.01 (0 to 0.02) | 5.55 (5.24 to 5.86) |
| Commonwealth Middle Income | 40 (20 to 74) | 0.01 (0 to 0.01) |  | 397 (222 to 562) | 0.02 (0.01 to 0.03) | 5.06 (4.91 to 5.21) |
| East Asia | 1589 (900 to 2017) | 0.15 (0.09 to 0.19) |  | 3631 (2396 to 4967) | 0.18 (0.12 to 0.25) | −0.52 (−1.06 to 0.02) |
| East Asia & Pacific - WB | 3084 (2346 to 3541) | 0.21 (0.17 to 0.24) |  | 7987 (6604 to 9536) | 0.25 (0.21 to 0.30) | 0.14 (−0.07 to 0.34) |
| Eastern Africa | 1 (1 to 2) | 0 (0 to 0) |  | 13 (12 to 14) | 0.01 (0.01 to 0.01) | 5.58 (3.73 to 7.47) |
| Eastern Europe | 226 (163 to 312) | 0.09 (0.06 to 0.12) |  | 2175 (1997 to 2366) | 0.64 (0.59 to 0.70) | 6.23 (5.61 to 6.85) |
| Eastern Mediterranean Region | 19 (13 to 28) | 0.01 (0 to 0.01) |  | 114 (86 to 143) | 0.02 (0.02 to 0.03) | 4.25 (3.97 to 4.53) |
| Eastern Sub-Saharan Africa | 1 (1 to 2) | 0 (0 to 0) |  | 1 (1 to 2) | 0 (0 to 0) | −3.28 (−4.12 to −2.42) |
| Europe | 7202 (6880 to 7483) | 0.72 (0.69 to 0.75) |  | 16895 (15348 to 18502) | 1.12 (1.03 to 1.23) | 1.72 (1.61 to 1.84) |
| Europe & Central Asia - WB | 7195 (6873 to 7475) | 0.70 (0.67 to 0.73) |  | 16885 (15341 to 18490) | 1.08 (0.99 to 1.17) | 1.71 (1.59 to 1.83) |
| European Region | 7240 (6918 to 7522) | 0.70 (0.66 to 0.73) |  | 17012 (15457 to 18628) | 1.07 (0.98 to 1.17) | 1.70 (1.58 to 1.82) |
| High-income Asia Pacific | 1095 (1046 to 1130) | 0.54 (0.52 to 0.56) |  | 3290 (2882 to 3614) | 0.72 (0.64 to 0.78) | 0.95 (0.79 to 1.11) |
| High-income North America | 4328 (4110 to 4455) | 1.27 (1.21 to 1.30) |  | 9571 (8809 to 10051) | 1.50 (1.39 to 1.57) | 0.64 (0.33 to 0.96) |
| Latin America & Caribbean - WB | 539 (525 to 552) | 0.18 (0.17 to 0.18) |  | 3997 (3682 to 4353) | 0.56 (0.52 to 0.61) | 3.80 (3.40 to 4.21) |
| Limited Health System | 59 (33 to 102) | 0.01 (0 to 0.01) |  | 444 (241 to 632) | 0.02 (0.01 to 0.03) | 4.31 (4.16 to 4.47) |
| Middle East & North Africa - WB | 59 (54 to 66) | 0.04 (0.04 to 0.05) |  | 202 (181 to 223) | 0.06 (0.06 to 0.07) | 1.16 (1.03 to 1.28) |
| Minimal Health System | 1 (1 to 1) | 0 (0 to 0) |  | 1 (1 to 2) | 0 (0 to 0) | −1.69 (−2.53 to −0.85) |
| North Africa and Middle East | 179 (106 to 302) | 0.06 (0.03 to 0.10) |  | 699 (482 to 981) | 0.14 (0.10 to 0.20) | 3.12 (2.87 to 3.37) |
| North America | 4328 (4109 to 4455) | 1.27 (1.21 to 1.30) |  | 9571 (8809 to 10051) | 1.50 (1.39 to 1.57) | 0.64 (0.33 to 0.96) |
| Northern Africa | 5 (3 to 6) | 0 (0 to 0.01) |  | 2 (1 to 5) | 0 (0 to 0) | −6.99 (−8.94 to −5.00) |
| Oceania | 0 (0 to 0) | 0.01 (0 to 0.01) |  | 0 (0 to 0) | 0 (0 to 0) | −2.51 (−3.24 to −1.76) |
| Region of the Americas | 4865 (4635 to 4998) | 0.78 (0.74 to 0.80) |  | 13530 (12547 to 14297) | 1.02 (0.94 to 1.07) | 0.92 (0.65 to 1.20) |
| South-East Asia Region | 58 (30 to 103) | 0.01 (0 to 0.01) |  | 420 (212 to 625) | 0.02 (0.01 to 0.03) | 3.99 (3.78 to 4.19) |
| South Asia | 32 (16 to 69) | 0 (0 to 0.01) |  | 348 (168 to 522) | 0.02 (0.01 to 0.03) | 5.23 (5.06 to 5.40) |
| South Asia - WB | 34 (17 to 71) | 0 (0 to 0.01) |  | 370 (181 to 549) | 0.02 (0.01 to 0.03) | 5.41 (5.27 to 5.55) |
| Southeast Asia | 26 (13 to 39) | 0.01 (0 to 0.01) |  | 137 (91 to 195) | 0.02 (0.01 to 0.03) | 2.73 (2.59 to 2.88) |
| Southern Africa | 5 (2 to 9) | 0.01 (0 to 0.01) |  | 6 (2 to 10) | 0 (0 to 0.01) | −2.65 (−2.87 to −2.42) |
| Southern Latin America | 35 (34 to 36) | 0.07 (0.07 to 0.08) |  | 637 (579 to 704) | 0.75 (0.68 to 0.83) | 6.18 (4.60 to 7.80) |
| Southern Sub-Saharan Africa | 5 (1 to 9) | 0.01 (0 to 0.02) |  | 5 (2 to 9) | 0.01 (0 to 0.01) | −2.23 (−2.42 to −2.05) |
| Sub-Saharan Africa - WB | 9 (5 to 13) | 0 (0 to 0) |  | 27 (21 to 33) | 0 (0 to 0) | 0.62 (−0.11 to 1.36) |
| Tropical Latin America | 314 (304 to 325) | 0.30 (0.29 to 0.31) |  | 1877 (1748 to 2010) | 0.73 (0.68 to 0.78) | 3.18 (3.06 to 3.29) |
| Western Africa | 2 (1 to 2) | 0 (0 to 0) |  | 7 (5 to 10) | 0 (0 to 0) | 2.06 (1.71 to 2.41) |
| Western Europe | 6549 (6289 to 6711) | 1.20 (1.16 to 1.23) |  | 13100 (11685 to 14514) | 1.49 (1.35 to 1.64) | 1.03 (0.87 to 1.19) |
| Western Pacific Region | 3011 (2287 to 3462) | 0.24 (0.19 to 0.28) |  | 7744 (6382 to 9234) | 0.29 (0.23 to 0.34) | 0.07 (−0.14 to 0.28) |
| Western Sub-Saharan Africa | 2 (1 to 2) | 0 (0 to 0) |  | 8 (6 to 11) | 0 (0 to 0) | 1.97 (1.63 to 2.30) |
| World Bank High Income | 12723 (12159 to 13006) | 1.02 (0.97 to 1.04) |  | 28639 (25732 to 31046) | 1.27 (1.16 to 1.37) | 0.93 (0.76 to 1.09) |
| World Bank Low Income | 17 (9 to 29) | 0.01 (0 to 0.01) |  | 23 (12 to 48) | 0.01 (0 to 0.01) | −0.86 (−1.04 to −0.67) |
| World Bank Lower Middle Income | 134 (88 to 201) | 0.01 (0.01 to 0.02) |  | 690 (461 to 897) | 0.03 (0.02 to 0.03) | 2.63 (2.43 to 2.83) |
| World Bank Upper Middle Income | 2375 (1612 to 2953) | 0.13 (0.09 to 0.17) |  | 9693 (8218 to 11211) | 0.28 (0.24 to 0.33) | 1.96 (1.68 to 2.24) |

Abbreviations: ASR, age-standardized rate; MND, motor neuron diseases; EAPC, estimated annual percentage change; SDI, sociodemographic index; GBD, Global Burden of Diseases, Injuries, and Risk Factors Study; UI, uncertainty interval; CI, confidence interval.

**Table S3.** The case number and ASR of DALYs of MND in 1990 and 2021 for both sex by SDI regions and by GBD regions, with EAPC from 1990 to 2021.

|  | **1990** | |  | **2021** | | **EAPC (95% CI)** |
| --- | --- | --- | --- | --- | --- | --- |
|  | **Number (95% UI)** | **ASR (95% UI)** |  | **Number (95% UI)** | **ASR (95% UI)** |  |
| Global | 506146 (462035 to 545050) | 11.22 (10.39 to 11.98) |  | 1040566 (963064 to 1123956) | 12.17 (11.24 to 13.15) | 0.30 (0.23 to 0.37) |
| **SDI region** |  |  |  |  |  |  |
| High-middle SDI | 109742 (90146 to 130338) | 10.96 (9.02 to 13.09) |  | 259530 (230126 to 290727) | 15.52 (13.52 to 17.57) | 0.79 (0.64 to 0.94) |
| High SDI | 320152 (311204 to 328059) | 32.46 (31.58 to 33.26) |  | 596797 (555483 to 636342) | 34.39 (32.48 to 36.49) | 0.40 (0.26 to 0.55) |
| Low-middle SDI | 9586 (8017 to 11593) | 0.89 (0.74 to 1.08) |  | 33386 (28573 to 38886) | 1.97 (1.70 to 2.29) | 2.83 (2.75 to 2.91) |
| Low SDI | 1565 (1119 to 2164) | 0.34 (0.24 to 0.46) |  | 4443 (3035 to 6008) | 0.47 (0.31 to 0.65) | 1.06 (0.89 to 1.23) |
| Middle SDI | 64713 (45775 to 76598) | 4.05 (2.95 to 4.75) |  | 145405 (124627 to 169676) | 5.45 (4.64 to 6.39) | 0.46 (0.16 to 0.76) |
| **GBD region** |  |  |  |  |  |  |
| Advanced Health System | 382104 (368954 to 395890) | 26.25 (25.30 to 27.51) |  | 766011 (717907 to 818570) | 32.72 (30.75 to 34.84) | 0.92 (0.79 to 1.05) |
| Africa | 2676 (1998 to 3520) | 0.45 (0.34 to 0.59) |  | 5172 (3795 to 7080) | 0.42 (0.32 to 0.57) | −0.41 (−0.60 to −0.22) |
| African Region | 1842 (1341 to 2506) | 0.41 (0.30 to 0.54) |  | 4156 (3105 to 5646) | 0.42 (0.32 to 0.56) | −0.01 (−0.18 to 0.17) |
| America | 147492 (142781 to 151409) | 23.08 (22.34 to 23.69) |  | 358497 (339730 to 376883) | 28.25 (26.76 to 29.69) | 0.79 (0.57 to 1.00) |
| Andean Latin America | 161 (116 to 225) | 0.49 (0.36 to 0.67) |  | 4963 (3823 to 6019) | 8.00 (6.17 to 9.71) | 9.97 (8.42 to 11.55) |
| Asia | 143030 (102840 to 169392) | 5.18 (3.89 to 6.05) |  | 252771 (201243 to 301064) | 5.12 (4.06 to 6.04) | −0.54 (−0.80 to −0.27) |
| Australasia | 10281 (9851 to 10701) | 46.81 (44.95 to 48.65) |  | 22758 (20520 to 24987) | 49.54 (45.12 to 54.17) | 0.44 (0.16 to 0.73) |
| Basic Health System | 115821 (77217 to 140240) | 5.38 (3.66 to 6.48) |  | 247799 (202513 to 297249) | 6.91 (5.61 to 8.23) | 0.18 (−0.14 to 0.51) |
| Caribbean | 305 (245 to 383) | 0.92 (0.74 to 1.13) |  | 6637 (5611 to 7756) | 12.75 (10.76 to 14.89) | 7.91 (5.59 to 10.28) |
| Central Africa | 184 (136 to 251) | 0.30 (0.22 to 0.41) |  | 411 (288 to 573) | 0.27 (0.19 to 0.37) | −0.40 (−0.55 to −0.24) |
| Central Asia | 572 (431 to 745) | 0.82 (0.62 to 1.05) |  | 1492 (1284 to 1745) | 1.56 (1.35 to 1.82) | 2.67 (2.22 to 3.11) |
| Central Europe | 13398 (12874 to 14034) | 11.09 (10.64 to 11.63) |  | 32148 (29468 to 35150) | 19.82 (18.27 to 21.71) | 2.30 (2.06 to 2.53) |
| Central Latin America | 8504 (8163 to 8892) | 6.76 (6.55 to 7.00) |  | 35995 (31564 to 40871) | 13.92 (12.20 to 15.83) | 2.44 (2.32 to 2.56) |
| Central Sub-Saharan Africa | 153 (111 to 212) | 0.31 (0.22 to 0.42) |  | 316 (211 to 456) | 0.26 (0.18 to 0.36) | −0.62 (−0.79 to −0.45) |
| Commonwealth High Income | 57309 (55851 to 58629) | 43.21 (42.19 to 44.18) |  | 107222 (100037 to 114282) | 46.62 (43.82 to 49.52) | 0.54 (0.27 to 0.82) |
| Commonwealth Low Income | 683 (496 to 934) | 0.35 (0.25 to 0.48) |  | 1849 (1171 to 2650) | 0.55 (0.33 to 0.82) | 1.38 (1.20 to 1.56) |
| Commonwealth Middle Income | 5384 (3912 to 7149) | 0.50 (0.36 to 0.68) |  | 20702 (14317 to 27057) | 1.09 (0.74 to 1.43) | 2.63 (2.50 to 2.77) |
| East Asia | 90418 (52778 to 114963) | 7.96 (4.73 to 10.09) |  | 128689 (86939 to 173235) | 7.73 (5.03 to 10.02) | −1.43 (−2.02 to −0.84) |
| East Asia & Pacific - WB | 135157 (96347 to 160221) | 8.18 (6.03 to 9.59) |  | 225529 (179885 to 271919) | 7.75 (6.03 to 9.31) | −0.84 (−1.16 to −0.53) |
| Eastern Africa | 503 (354 to 707) | 0.32 (0.23 to 0.44) |  | 1377 (1026 to 1836) | 0.48 (0.39 to 0.60) | 0.98 (0.61 to 1.36) |
| Eastern Europe | 10039 (7604 to 12852) | 4.16 (3.20 to 5.31) |  | 64276 (59371 to 69494) | 21.06 (19.54 to 22.69) | 4.94 (4.41 to 5.48) |
| Eastern Mediterranean Region | 2756 (2110 to 3533) | 0.74 (0.57 to 0.96) |  | 7753 (6270 to 9669) | 1.17 (0.95 to 1.43) | 1.76 (1.58 to 1.95) |
| Eastern Sub-Saharan Africa | 522 (368 to 734) | 0.30 (0.22 to 0.42) |  | 1071 (695 to 1555) | 0.28 (0.19 to 0.39) | −0.37 (−0.46 to −0.27) |
| Europe | 212504 (203834 to 224394) | 23.74 (22.67 to 25.56) |  | 422076 (390518 to 456793) | 33.06 (30.76 to 35.81) | 1.30 (1.19 to 1.41) |
| Europe & Central Asia - WB | 212501 (203823 to 224421) | 22.52 (21.51 to 24.17) |  | 422361 (390754 to 457075) | 31.07 (28.90 to 33.63) | 1.28 (1.16 to 1.39) |
| European Region | 214049 (205331 to 225997) | 22.52 (21.52 to 24.16) |  | 425656 (393760 to 460611) | 30.95 (28.79 to 33.50) | 1.27 (1.15 to 1.38) |
| High-income Asia Pacific | 31936 (30422 to 33171) | 16.35 (15.46 to 17.01) |  | 68295 (61566 to 73932) | 18.11 (16.76 to 19.47) | 0.39 (0.22 to 0.56) |
| High-income North America | 123874 (119504 to 127252) | 39.82 (38.60 to 40.85) |  | 237212 (224879 to 247051) | 41.58 (39.69 to 43.26) | 0.30 (0.04 to 0.56) |
| Latin America & Caribbean - WB | 23677 (22739 to 24768) | 6.61 (6.39 to 6.85) |  | 122325 (112711 to 132839) | 17.32 (15.94 to 18.82) | 3.20 (2.89 to 3.51) |
| Limited Health System | 7455 (5486 to 9923) | 0.52 (0.37 to 0.70) |  | 24873 (17308 to 32577) | 0.98 (0.67 to 1.29) | 2.15 (1.99 to 2.31) |
| Middle East & North Africa - WB | 3640 (3146 to 4270) | 1.78 (1.57 to 2.03) |  | 8516 (7449 to 9769) | 2.12 (1.89 to 2.40) | 0.76 (0.64 to 0.88) |
| Minimal Health System | 379 (269 to 530) | 0.31 (0.22 to 0.43) |  | 878 (590 to 1253) | 0.29 (0.20 to 0.40) | −0.21 (−0.29 to −0.12) |
| North Africa and Middle East | 13111 (8421 to 21978) | 3.57 (2.33 to 5.66) |  | 28917 (21768 to 38491) | 5.27 (3.93 to 7.02) | 1.66 (1.46 to 1.86) |
| North America | 123867 (119498 to 127246) | 39.81 (38.60 to 40.85) |  | 237216 (224881 to 247053) | 41.58 (39.69 to 43.26) | 0.30 (0.04 to 0.56) |
| Northern Africa | 893 (686 to 1182) | 0.71 (0.54 to 0.91) |  | 1087 (738 to 1533) | 0.52 (0.36 to 0.73) | −1.43 (−1.72 to −1.14) |
| Oceania | 26 (19 to 35) | 0.46 (0.35 to 0.59) |  | 47 (35 to 64) | 0.37 (0.28 to 0.50) | −0.91 (−1.10 to −0.72) |
| Region of the Americas | 147492 (142781 to 151409) | 23.08 (22.34 to 23.69) |  | 358497 (339730 to 376883) | 28.25 (26.76 to 29.69) | 0.79 (0.57 to 1.00) |
| South-East Asia Region | 6646 (4845 to 8917) | 0.55 (0.39 to 0.77) |  | 21163 (14203 to 28285) | 1.06 (0.70 to 1.42) | 2.14 (1.97 to 2.32) |
| South Asia | 4801 (3429 to 6568) | 0.47 (0.33 to 0.67) |  | 18450 (12113 to 24784) | 1.08 (0.69 to 1.45) | 2.75 (2.57 to 2.92) |
| South Asia - WB | 4955 (3540 to 6757) | 0.48 (0.34 to 0.67) |  | 19315 (12671 to 25887) | 1.10 (0.70 to 1.47) | 2.84 (2.67 to 3.00) |
| Southeast Asia | 2543 (1872 to 3285) | 0.61 (0.44 to 0.80) |  | 6739 (4964 to 8644) | 0.95 (0.70 to 1.21) | 1.38 (1.31 to 1.45) |
| Southern Africa | 548 (371 to 793) | 0.67 (0.44 to 0.96) |  | 837 (552 to 1179) | 0.49 (0.33 to 0.69) | −1.13 (−1.22 to −1.04) |
| Southern Latin America | 1543 (1417 to 1688) | 3.21 (2.96 to 3.51) |  | 18154 (16602 to 19792) | 22.70 (20.73 to 24.72) | 5.32 (3.94 to 6.73) |
| Southern Sub-Saharan Africa | 423 (266 to 671) | 0.89 (0.53 to 1.40) |  | 557 (346 to 803) | 0.68 (0.44 to 0.97) | −1.03 (−1.13 to −0.92) |
| Sub-Saharan Africa - WB | 1789 (1304 to 2432) | 0.39 (0.29 to 0.52) |  | 4099 (3061 to 5570) | 0.41 (0.31 to 0.54) | 0 (−0.18 to 0.18) |
| Tropical Latin America | 13176 (12688 to 13825) | 10.87 (10.50 to 11.31) |  | 56816 (53279 to 60714) | 22.24 (20.86 to 23.76) | 2.61 (2.48 to 2.73) |
| Western Africa | 547 (394 to 751) | 0.34 (0.25 to 0.46) |  | 1461 (1074 to 1968) | 0.37 (0.27 to 0.49) | 0.32 (0.22 to 0.42) |
| Western Europe | 179755 (174002 to 183650) | 38.34 (37.36 to 39.13) |  | 305400 (278825 to 334428) | 41.57 (38.30 to 45.31) | 0.56 (0.41 to 0.71) |
| Western Pacific Region | 130918 (92780 to 155560) | 9.51 (6.95 to 11.18) |  | 216168 (171224 to 262326) | 9.00 (6.90 to 10.87) | −0.92 (−1.26 to −0.59) |
| Western Sub-Saharan Africa | 604 (437 to 827) | 0.34 (0.25 to 0.45) |  | 1635 (1203 to 2205) | 0.37 (0.28 to 0.49) | 0.31 (0.21 to 0.40) |
| World Bank High Income | 361574 (351538 to 369561) | 31.91 (31.10 to 32.59) |  | 683227 (635035 to 732605) | 35.27 (33.10 to 37.59) | 0.56 (0.40 to 0.71) |
| World Bank Low Income | 1768 (1274 to 2448) | 0.61 (0.43 to 0.83) |  | 2854 (2023 to 4118) | 0.48 (0.34 to 0.71) | −0.74 (−0.83 to −0.64) |
| World Bank Lower Middle Income | 12431 (9514 to 16047) | 0.72 (0.55 to 0.95) |  | 35537 (26865 to 44615) | 1.17 (0.89 to 1.47) | 1.37 (1.27 to 1.47) |
| World Bank Upper Middle Income | 129775 (88809 to 162042) | 6.67 (4.64 to 8.27) |  | 317646 (267020 to 369680) | 10.17 (8.42 to 11.79) | 0.79 (0.48 to 1.10) |

Abbreviations: ASR, age-standardized rate; MND, motor neuron diseases; EAPC, estimated annual percentage change; SDI, sociodemographic index; GBD, Global Burden of Diseases, Injuries, and Risk Factors Study; UI, uncertainty interval; CI, confidence interval.

**Table S4.** The case number and ASR of incidence of MND in 1990 and 2021, with EAPC from 1990 to 2021.

|  | **1990** | | **2021** | | **EAPC (95% CI)** |
| --- | --- | --- | --- | --- | --- |
|  | **Number (95% UI)** | **ASR (95% UI)** | **Number (95% UI)** | **ASR (95% UI)** |  |
| **Sex** |  |  |  |  |  |
| Female | 17084 (15300 to 19087) | 0.72 (0.65 to 0.81) | 29040 (26390 to 31866) | 0.67 (0.61 to 0.74) | −0.21 (−0.25 to −0.16) |
| Male | 19685 (17693 to 22183) | 0.90 (0.81 to 1.00) | 35138 (32151 to 38498) | 0.88 (0.81 to 0.96) | 0.00 (−0.05 to 0.05) |
| **Age** |  |  |  |  |  |
| <5 years | 6902 (5677 to 8343) | 1.11 (0.92 to 1.35) | 4951 (3751 to 6564) | 0.75 (0.57 to 1.00) | −1.35 (−1.41 to −1.28) |
| 5-9 years | 1313 (864 to 1775) | 0.23 (0.15 to 0.30) | 1408 (866 to 1943) | 0.20 (0.13 to 0.28) | −0.37 (−0.42 to −0.33) |
| 10-14 years | 751 (373 to 1260) | 0.14 (0.07 to 0.24) | 821 (402 to 1394) | 0.12 (0.06 to 0.21) | −0.51 (−0.58 to −0.44) |
| 15-19 years | 608 (284 to 1064) | 0.12 (0.05 to 0.20) | 628 (285 to 1101) | 0.10 (0.05 to 0.18) | −0.52 (−0.59 to −0.44) |
| 20-24 years | 652 (287 to 1230) | 0.13 (0.06 to 0.25) | 687 (305 to 1308) | 0.11 (0.05 to 0.22) | −0.47 (−0.52 to −0.41) |
| 25-29 years | 850 (401 to 1477) | 0.19 (0.09 to 0.33) | 990 (463 to 1720) | 0.17 (0.08 to 0.29) | −0.44 (−0.51 to −0.38) |
| 30-34 years | 1113 (569 to 2009) | 0.29 (0.15 to 0.52) | 1512 (772 to 2672) | 0.25 (0.13 to 0.44) | −0.47 (−0.55 to −0.39) |
| 35-39 years | 1531 (843 to 2646) | 0.43 (0.24 to 0.75) | 2092 (1165 to 3604) | 0.37 (0.21 to 0.64) | −0.51 (−0.59 to −0.42) |
| 40-44 years | 1751 (957 to 2971) | 0.61 (0.33 to 1.04) | 2602 (1467 to 4339) | 0.52 (0.29 to 0.87) | −0.54 (−0.64 to −0.44) |
| 45-49 years | 1899 (1068 to 2981) | 0.82 (0.46 to 1.28) | 3326 (1930 to 5118) | 0.70 (0.41 to 1.08) | −0.51 (−0.60 to −0.42) |
| 50-54 years | 2349 (1512 to 3358) | 1.11 (0.71 to 1.58) | 4427 (2956 to 6146) | 0.99 (0.66 to 1.38) | −0.33 (−0.41 to −0.25) |
| 55-59 years | 2837 (1989 to 3836) | 1.53 (1.07 to 2.07) | 5766 (4202 to 7554) | 1.46 (1.06 to 1.91) | −0.03 (−0.14 to 0.08) |
| 60-64 years | 3502 (2695 to 4441) | 2.18 (1.68 to 2.77) | 7150 (5617 to 8805) | 2.23 (1.75 to 2.75) | 0.21 (0.09 to 0.33) |
| 65-69 years | 3651 (2834 to 4631) | 2.95 (2.29 to 3.75) | 8118 (6496 to 10059) | 2.94 (2.36 to 3.65) | 0.33 (0.22 to 0.44) |
| 70-74 years | 2874 (2279 to 3446) | 3.39 (2.69 to 4.07) | 7975 (6557 to 9323) | 3.87 (3.19 to 4.53) | 0.46 (0.42 to 0.49) |
| 75-79 years | 2306 (1855 to 2764) | 3.75 (3.01 to 4.49) | 5655 (4658 to 6571) | 4.29 (3.53 to 4.98) | 0.38 (0.29 to 0.47) |
| 80-84 years | 1280 (996 to 1573) | 3.62 (2.82 to 4.45) | 3671 (3011 to 4406) | 4.19 (3.44 to 5.03) | 0.45 (0.32 to 0.59) |
| 85-89 years | 465 (357 to 588) | 3.08 (2.37 to 3.89) | 1668 (1336 to 2047) | 3.65 (2.92 to 4.48) | 0.71 (0.58 to 0.84) |
| 90-94 years | 110 (79 to 149) | 2.57 (1.84 to 3.47) | 566 (434 to 719) | 3.17 (2.43 to 4.02) | 0.88 (0.79 to 0.97) |
| 95+ years | 25 (14 to 41) | 2.47 (1.39 to 4.01) | 164 (105 to 240) | 3.01 (1.92 to 4.40) | 0.83 (0.73 to 0.93) |
| **Country** |  |  |  |  |  |
| Afghanistan | 62 (51 to 73) | 0.62 (0.52 to 0.75) | 158 (129 to 190) | 0.57 (0.48 to 0.69) | −0.31 (−0.40 to −0.22) |
| Albania | 16 (13 to 18) | 0.52 (0.44 to 0.62) | 15 (12 to 17) | 0.48 (0.41 to 0.56) | −0.29 (−0.35 to −0.22) |
| Algeria | 100 (84 to 118) | 0.46 (0.38 to 0.55) | 184 (150 to 222) | 0.42 (0.35 to 0.51) | −0.28 (−0.34 to −0.21) |
| American Samoa | 0 (0 to 0) | 0.49 (0.42 to 0.56) | 0 (0 to 0) | 0.38 (0.32 to 0.45) | −0.98 (−1.08 to −0.88) |
| Andorra | 1 (1 to 1) | 1.91 (1.75 to 2.09) | 3 (3 to 3) | 2.25 (2.07 to 2.43) | 0.61 (0.58 to 0.64) |
| Angola | 36 (29 to 43) | 0.50 (0.40 to 0.63) | 101 (82 to 123) | 0.44 (0.35 to 0.54) | −0.48 (−0.56 to −0.40) |
| Antigua and Barbuda | 0 (0 to 0) | 0.53 (0.44 to 0.61) | 1 (1 to 1) | 0.66 (0.59 to 0.73) | 0.91 (0.87 to 0.95) |
| Argentina | 247 (212 to 283) | 0.75 (0.65 to 0.87) | 487 (446 to 530) | 0.97 (0.88 to 1.05) | 0.83 (0.77 to 0.89) |
| Armenia | 14 (11 to 17) | 0.43 (0.35 to 0.52) | 13 (11 to 16) | 0.39 (0.32 to 0.47) | −0.34 (−0.40 to −0.29) |
| Australia | 391 (371 to 412) | 2.08 (1.97 to 2.19) | 1070 (1027 to 1116) | 2.60 (2.49 to 2.71) | 0.78 (0.71 to 0.85) |
| Austria | 119 (109 to 130) | 1.24 (1.13 to 1.35) | 232 (219 to 246) | 1.60 (1.49 to 1.70) | 0.90 (0.81 to 1.00) |
| Azerbaijan | 29 (24 to 35) | 0.44 (0.36 to 0.54) | 42 (34 to 51) | 0.40 (0.33 to 0.48) | −0.40 (−0.48 to −0.33) |
| Bahrain | 2 (1 to 2) | 0.42 (0.35 to 0.51) | 6 (4 to 7) | 0.40 (0.33 to 0.49) | −0.20 (−0.26 to −0.14) |
| Bangladesh | 348 (286 to 423) | 0.39 (0.32 to 0.48) | 549 (450 to 668) | 0.36 (0.29 to 0.43) | −0.27 (−0.33 to −0.22) |
| Barbados | 2 (1 to 2) | 0.63 (0.55 to 0.72) | 5 (5 to 6) | 1.19 (1.11 to 1.27) | 1.99 (1.79 to 2.18) |
| Belarus | 81 (70 to 91) | 0.70 (0.60 to 0.80) | 118 (107 to 128) | 0.89 (0.80 to 0.97) | 0.80 (0.71 to 0.90) |
| Belgium | 298 (279 to 316) | 2.11 (1.98 to 2.24) | 417 (396 to 437) | 2.11 (1.99 to 2.24) | −0.02 (−0.12 to 0.08) |
| Belize | 1 (1 to 1) | 0.55 (0.46 to 0.64) | 2 (2 to 2) | 0.54 (0.47 to 0.61) | −0.01 (−0.06 to 0.04) |
| Benin | 15 (12 to 18) | 0.41 (0.33 to 0.51) | 38 (31 to 46) | 0.38 (0.30 to 0.46) | −0.29 (−0.35 to −0.22) |
| Bermuda | 1 (1 to 1) | 1.08 (0.98 to 1.17) | 1 (1 to 1) | 1.13 (1.05 to 1.21) | 0.14 (0.12 to 0.17) |
| Bhutan | 2 (2 to 2) | 0.39 (0.32 to 0.49) | 3 (2 to 3) | 0.36 (0.30 to 0.43) | −0.32 (−0.37 to −0.26) |
| Bolivia | 27 (23 to 31) | 0.55 (0.47 to 0.63) | 67 (60 to 75) | 0.65 (0.58 to 0.72) | 0.75 (0.69 to 0.81) |
| Bosnia and Herzegovina | 26 (22 to 31) | 0.62 (0.53 to 0.71) | 22 (17 to 27) | 0.52 (0.43 to 0.62) | −0.73 (−0.83 to −0.64) |
| Botswana | 4 (3 to 5) | 0.44 (0.35 to 0.53) | 8 (6 to 10) | 0.39 (0.32 to 0.47) | −0.41 (−0.47 to −0.34) |
| Brazil | 784 (675 to 893) | 0.65 (0.57 to 0.74) | 2078 (1891 to 2253) | 0.87 (0.79 to 0.94) | 1.16 (1.09 to 1.23) |
| Brunei | 1 (1 to 1) | 0.48 (0.40 to 0.57) | 2 (2 to 2) | 0.47 (0.40 to 0.53) | −0.01 (−0.07 to 0.06) |
| Bulgaria | 54 (44 to 64) | 0.57 (0.48 to 0.66) | 44 (36 to 53) | 0.52 (0.44 to 0.61) | −0.30 (−0.36 to −0.23) |
| Burkina Faso | 33 (27 to 39) | 0.44 (0.36 to 0.55) | 71 (58 to 85) | 0.41 (0.33 to 0.50) | −0.28 (−0.35 to −0.21) |
| Burundi | 18 (15 to 22) | 0.47 (0.37 to 0.59) | 41 (33 to 51) | 0.46 (0.36 to 0.57) | −0.10 (−0.16 to −0.03) |
| Cambodia | 38 (32 to 45) | 0.44 (0.36 to 0.53) | 60 (50 to 72) | 0.39 (0.32 to 0.47) | −0.43 (−0.50 to −0.35) |
| Cameroon | 30 (25 to 36) | 0.38 (0.31 to 0.46) | 83 (69 to 100) | 0.35 (0.29 to 0.42) | −0.29 (−0.35 to −0.23) |
| Canada | 644 (606 to 687) | 2.09 (1.96 to 2.23) | 1566 (1487 to 1643) | 2.45 (2.32 to 2.58) | 0.57 (0.52 to 0.62) |
| Cape Verde | 1 (1 to 1) | 0.42 (0.33 to 0.52) | 2 (1 to 2) | 0.37 (0.29 to 0.45) | −0.49 (−0.56 to −0.42) |
| Central African Republic | 10 (8 to 12) | 0.51 (0.40 to 0.64) | 19 (15 to 23) | 0.48 (0.39 to 0.61) | −0.19 (−0.26 to −0.12) |
| Chad | 22 (18 to 26) | 0.46 (0.38 to 0.58) | 56 (46 to 67) | 0.43 (0.35 to 0.53) | −0.31 (−0.39 to −0.24) |
| Chile | 103 (91 to 115) | 0.90 (0.80 to 0.99) | 264 (245 to 284) | 1.15 (1.06 to 1.23) | 0.90 (0.85 to 0.95) |
| China | 6854 (5929 to 7957) | 0.65 (0.57 to 0.75) | 7325 (5993 to 8709) | 0.46 (0.39 to 0.54) | −1.33 (−1.51 to −1.16) |
| Colombia | 124 (104 to 143) | 0.47 (0.40 to 0.53) | 351 (315 to 383) | 0.68 (0.62 to 0.75) | 1.55 (1.46 to 1.64) |
| Comoros | 2 (1 to 2) | 0.49 (0.39 to 0.60) | 3 (2 to 3) | 0.45 (0.36 to 0.56) | −0.25 (−0.31 to −0.18) |
| Congo | 7 (6 to 8) | 0.41 (0.32 to 0.52) | 16 (12 to 20) | 0.37 (0.30 to 0.47) | −0.33 (−0.40 to −0.26) |
| Cook Islands | 0 (0 to 0) | 0.49 (0.41 to 0.56) | 0 (0 to 0) | 0.39 (0.32 to 0.45) | −0.89 (−0.96 to −0.81) |
| Costa Rica | 13 (11 to 15) | 0.55 (0.47 to 0.62) | 62 (58 to 66) | 1.18 (1.11 to 1.26) | 2.39 (2.12 to 2.66) |
| Cote d'Ivoire | 34 (28 to 42) | 0.39 (0.31 to 0.48) | 77 (63 to 94) | 0.36 (0.29 to 0.44) | −0.26 (−0.31 to −0.20) |
| Croatia | 38 (33 to 42) | 0.74 (0.65 to 0.82) | 52 (47 to 56) | 0.84 (0.77 to 0.92) | 0.53 (0.48 to 0.58) |
| Cuba | 58 (50 to 67) | 0.58 (0.49 to 0.66) | 128 (116 to 139) | 0.81 (0.74 to 0.88) | 1.17 (1.08 to 1.25) |
| Cyprus | 9 (8 to 10) | 1.14 (1.03 to 1.24) | 23 (21 to 24) | 1.21 (1.11 to 1.30) | 0.26 (0.23 to 0.30) |
| Czech Republic | 74 (65 to 83) | 0.67 (0.59 to 0.76) | 113 (102 to 124) | 0.75 (0.67 to 0.82) | 0.43 (0.39 to 0.46) |
| Democratic Republic of the Congo | 118 (97 to 142) | 0.44 (0.35 to 0.55) | 269 (220 to 324) | 0.42 (0.34 to 0.52) | −0.17 (−0.25 to −0.09) |
| Denmark | 139 (131 to 148) | 1.96 (1.84 to 2.09) | 213 (202 to 224) | 2.09 (1.98 to 2.23) | 0.21 (0.16 to 0.26) |
| Djibouti | 1 (1 to 2) | 0.48 (0.38 to 0.60) | 5 (4 to 6) | 0.45 (0.36 to 0.56) | −0.28 (−0.35 to −0.21) |
| Dominica | 0 (0 to 0) | 0.58 (0.50 to 0.66) | 1 (1 to 1) | 0.93 (0.85 to 1.01) | 1.70 (1.58 to 1.82) |
| Dominican Republic | 32 (27 to 37) | 0.56 (0.47 to 0.64) | 67 (59 to 75) | 0.64 (0.57 to 0.71) | 0.72 (0.62 to 0.83) |
| Ecuador | 30 (25 to 35) | 0.38 (0.32 to 0.46) | 91 (80 to 101) | 0.54 (0.48 to 0.60) | 1.42 (1.31 to 1.52) |
| Egypt | 247 (205 to 294) | 0.50 (0.41 to 0.60) | 438 (362 to 515) | 0.45 (0.38 to 0.53) | −0.31 (−0.37 to −0.25) |
| El Salvador | 21 (18 to 25) | 0.49 (0.40 to 0.57) | 27 (22 to 31) | 0.43 (0.36 to 0.51) | −0.38 (−0.45 to −0.32) |
| Equatorial Guinea | 1 (1 to 2) | 0.42 (0.34 to 0.53) | 3 (3 to 4) | 0.34 (0.27 to 0.42) | −0.88 (−1 to −0.76) |
| Eritrea | 13 (10 to 16) | 0.56 (0.45 to 0.71) | 24 (20 to 30) | 0.51 (0.41 to 0.64) | −0.32 (−0.40 to −0.24) |
| Estonia | 10 (8 to 12) | 0.57 (0.48 to 0.68) | 9 (7 to 10) | 0.53 (0.45 to 0.62) | −0.26 (−0.32 to −0.21) |
| Ethiopia | 185 (150 to 230) | 0.52 (0.41 to 0.66) | 343 (277 to 433) | 0.44 (0.35 to 0.56) | −0.61 (−0.75 to −0.47) |
| Federated States of Micronesia | 0 (0 to 0) | 0.46 (0.39 to 0.53) | 0 (0 to 0) | 0.42 (0.35 to 0.49) | −0.36 (−0.42 to −0.31) |
| Fiji | 3 (2 to 3) | 0.44 (0.37 to 0.53) | 4 (3 to 4) | 0.41 (0.34 to 0.48) | −0.29 (−0.35 to −0.23) |
| Finland | 166 (158 to 175) | 2.52 (2.39 to 2.67) | 195 (185 to 207) | 2.00 (1.88 to 2.13) | −0.90 (−1.08 to −0.72) |
| France | 1407 (1328 to 1491) | 1.93 (1.82 to 2.05) | 2544 (2428 to 2664) | 2.23 (2.11 to 2.35) | 0.49 (0.46 to 0.52) |
| Gabon | 3 (2 to 3) | 0.37 (0.29 to 0.46) | 5 (4 to 6) | 0.34 (0.27 to 0.42) | −0.31 (−0.37 to −0.24) |
| Georgia | 24 (20 to 29) | 0.43 (0.36 to 0.52) | 19 (15 to 22) | 0.43 (0.36 to 0.51) | 0.06 (0.03 to 0.10) |
| Germany | 1511 (1401 to 1618) | 1.41 (1.31 to 1.51) | 2675 (2520 to 2813) | 1.73 (1.63 to 1.83) | 0.79 (0.74 to 0.83) |
| Ghana | 44 (36 to 53) | 0.39 (0.32 to 0.49) | 95 (78 to 115) | 0.35 (0.29 to 0.42) | −0.40 (−0.46 to −0.34) |
| Greece | 101 (88 to 114) | 0.82 (0.71 to 0.91) | 218 (204 to 232) | 1.27 (1.18 to 1.36) | 1.72 (1.60 to 1.84) |
| Greenland | 1 (1 to 1) | 1.69 (1.44 to 1.97) | 1 (1 to 1) | 1.49 (1.30 to 1.71) | −0.39 (−0.48 to −0.29) |
| Grenada | 0 (0 to 0) | 0.55 (0.47 to 0.64) | 1 (1 to 1) | 0.60 (0.53 to 0.67) | 0.15 (0.04 to 0.27) |
| Guam | 1 (1 to 1) | 0.83 (0.75 to 0.90) | 1 (1 to 1) | 0.35 (0.29 to 0.42) | −3.57 (−3.96 to −3.17) |
| Guatemala | 36 (30 to 42) | 0.52 (0.43 to 0.61) | 63 (53 to 74) | 0.47 (0.40 to 0.56) | −0.29 (−0.36 to −0.23) |
| Guinea | 21 (17 to 25) | 0.42 (0.34 to 0.52) | 40 (33 to 48) | 0.39 (0.32 to 0.48) | −0.26 (−0.33 to −0.20) |
| Guinea-Bissau | 3 (3 to 4) | 0.45 (0.36 to 0.55) | 6 (5 to 8) | 0.41 (0.34 to 0.51) | −0.28 (−0.35 to −0.22) |
| Guyana | 3 (3 to 4) | 0.51 (0.43 to 0.60) | 4 (4 to 5) | 0.60 (0.53 to 0.67) | 0.67 (0.62 to 0.72) |
| Haiti | 37 (31 to 42) | 0.65 (0.55 to 0.74) | 71 (62 to 81) | 0.65 (0.58 to 0.73) | 0.08 (0.04 to 0.12) |
| Honduras | 20 (17 to 24) | 0.54 (0.46 to 0.63) | 51 (45 to 58) | 0.64 (0.57 to 0.72) | 0.77 (0.71 to 0.82) |
| Hungary | 86 (75 to 96) | 0.78 (0.70 to 0.86) | 112 (102 to 124) | 0.87 (0.80 to 0.94) | 0.47 (0.42 to 0.52) |
| Iceland | 5 (5 to 5) | 1.82 (1.69 to 1.96) | 10 (9 to 11) | 2.02 (1.89 to 2.15) | 0.38 (0.35 to 0.40) |
| India | 2926 (2402 to 3604) | 0.39 (0.32 to 0.49) | 4858 (3978 to 5902) | 0.37 (0.30 to 0.44) | −0.20 (−0.28 to −0.13) |
| Indonesia | 567 (462 to 692) | 0.37 (0.30 to 0.45) | 853 (694 to 1050) | 0.32 (0.26 to 0.38) | −0.51 (−0.61 to −0.42) |
| Iran | 274 (230 to 323) | 0.55 (0.46 to 0.68) | 415 (339 to 503) | 0.50 (0.42 to 0.59) | −0.39 (−0.46 to −0.32) |
| Iraq | 86 (71 to 104) | 0.54 (0.44 to 0.66) | 174 (145 to 211) | 0.48 (0.40 to 0.59) | −0.40 (−0.47 to −0.33) |
| Ireland | 87 (82 to 92) | 2.23 (2.11 to 2.35) | 212 (201 to 224) | 2.91 (2.76 to 3.07) | 1.03 (0.98 to 1.07) |
| Israel | 58 (54 to 63) | 1.21 (1.11 to 1.31) | 143 (133 to 153) | 1.28 (1.19 to 1.37) | 0.22 (0.20 to 0.24) |
| Italy | 1020 (947 to 1098) | 1.38 (1.28 to 1.48) | 2110 (1992 to 2223) | 1.84 (1.74 to 1.96) | 0.97 (0.88 to 1.06) |
| Jamaica | 11 (10 to 13) | 0.56 (0.48 to 0.65) | 17 (15 to 19) | 0.61 (0.53 to 0.68) | 0.35 (0.29 to 0.40) |
| Japan | 1381 (1250 to 1517) | 0.89 (0.80 to 0.97) | 2831 (2661 to 2998) | 1.04 (0.96 to 1.11) | 0.59 (0.56 to 0.62) |
| Jordan | 16 (13 to 19) | 0.50 (0.41 to 0.60) | 49 (41 to 59) | 0.46 (0.39 to 0.55) | −0.26 (−0.33 to −0.20) |
| Kazakhstan | 78 (64 to 96) | 0.51 (0.42 to 0.64) | 89 (72 to 108) | 0.46 (0.38 to 0.56) | −0.40 (−0.46 to −0.34) |
| Kenya | 66 (54 to 81) | 0.42 (0.33 to 0.54) | 132 (104 to 166) | 0.35 (0.28 to 0.44) | −0.70 (−0.82 to −0.57) |
| Kiribati | 0 (0 to 0) | 0.76 (0.68 to 0.84) | 1 (1 to 1) | 0.85 (0.77 to 0.92) | 0.44 (0.42 to 0.47) |
| Kuwait | 7 (6 to 9) | 0.53 (0.45 to 0.61) | 18 (15 to 23) | 0.46 (0.39 to 0.53) | −0.51 (−0.55 to −0.48) |
| Kyrgyzstan | 18 (15 to 22) | 0.45 (0.37 to 0.55) | 27 (23 to 32) | 0.43 (0.35 to 0.51) | −0.19 (−0.25 to −0.13) |
| Laos | 17 (14 to 20) | 0.48 (0.39 to 0.57) | 27 (22 to 32) | 0.42 (0.35 to 0.50) | −0.48 (−0.55 to −0.41) |
| Latvia | 17 (13 to 20) | 0.57 (0.47 to 0.68) | 15 (13 to 17) | 0.59 (0.51 to 0.67) | 0.28 (0.21 to 0.36) |
| Lebanon | 14 (11 to 16) | 0.48 (0.40 to 0.58) | 24 (20 to 29) | 0.44 (0.37 to 0.53) | −0.30 (−0.36 to −0.24) |
| Lesotho | 7 (5 to 8) | 0.54 (0.44 to 0.67) | 7 (6 to 9) | 0.47 (0.39 to 0.58) | −0.47 (−0.54 to −0.39) |
| Liberia | 8 (6 to 9) | 0.40 (0.32 to 0.49) | 16 (13 to 19) | 0.38 (0.31 to 0.46) | −0.26 (−0.33 to −0.19) |
| Libya | 15 (13 to 18) | 0.42 (0.35 to 0.51) | 25 (20 to 30) | 0.40 (0.33 to 0.47) | −0.22 (−0.30 to −0.14) |
| Lithuania | 23 (19 to 27) | 0.59 (0.50 to 0.68) | 44 (41 to 47) | 1.03 (0.95 to 1.12) | 2.03 (1.85 to 2.20) |
| Luxembourg | 8 (7 to 8) | 1.58 (1.47 to 1.71) | 14 (13 to 15) | 1.56 (1.45 to 1.66) | −0.09 (−0.16 to −0.02) |
| Macedonia | 11 (10 to 13) | 0.60 (0.52 to 0.69) | 12 (10 to 15) | 0.51 (0.42 to 0.60) | −0.66 (−0.73 to −0.59) |
| Madagascar | 48 (40 to 58) | 0.58 (0.46 to 0.73) | 106 (87 to 130) | 0.53 (0.43 to 0.67) | −0.31 (−0.38 to −0.24) |
| Malawi | 37 (30 to 45) | 0.54 (0.43 to 0.69) | 65 (52 to 80) | 0.50 (0.39 to 0.62) | −0.34 (−0.41 to −0.27) |
| Malaysia | 47 (38 to 57) | 0.33 (0.26 to 0.40) | 88 (71 to 108) | 0.29 (0.24 to 0.36) | −0.34 (−0.40 to −0.28) |
| Maldives | 1 (1 to 1) | 0.37 (0.30 to 0.42) | 2 (1 to 2) | 0.35 (0.30 to 0.41) | −0.09 (−0.16 to −0.02) |
| Mali | 68 (60 to 75) | 1.16 (1.04 to 1.29) | 157 (142 to 173) | 1.13 (1.03 to 1.23) | −0.14 (−0.19 to −0.09) |
| Malta | 6 (5 to 6) | 1.37 (1.27 to 1.47) | 12 (12 to 13) | 1.55 (1.45 to 1.65) | 0.46 (0.44 to 0.48) |
| Marshall Islands | 0 (0 to 0) | 0.47 (0.40 to 0.55) | 0 (0 to 0) | 0.41 (0.35 to 0.48) | −0.49 (−0.54 to −0.43) |
| Mauritania | 7 (6 to 9) | 0.45 (0.37 to 0.56) | 14 (12 to 17) | 0.41 (0.34 to 0.51) | −0.32 (−0.38 to −0.25) |
| Mauritius | 4 (3 to 5) | 0.44 (0.37 to 0.51) | 6 (5 to 7) | 0.41 (0.35 to 0.48) | −0.17 (−0.24 to −0.10) |
| Mexico | 394 (335 to 456) | 0.55 (0.47 to 0.64) | 815 (717 to 909) | 0.64 (0.57 to 0.72) | 0.70 (0.64 to 0.77) |
| Moldova | 24 (20 to 28) | 0.53 (0.45 to 0.62) | 21 (17 to 25) | 0.50 (0.43 to 0.58) | −0.16 (−0.22 to −0.10) |
| Mongolia | 10 (9 to 12) | 0.56 (0.47 to 0.66) | 16 (14 to 19) | 0.51 (0.43 to 0.60) | −0.32 (−0.36 to −0.27) |
| Montenegro | 3 (3 to 4) | 0.53 (0.44 to 0.64) | 4 (3 to 4) | 0.49 (0.41 to 0.59) | −0.30 (−0.37 to −0.23) |
| Morocco | 118 (98 to 142) | 0.53 (0.44 to 0.65) | 172 (141 to 207) | 0.47 (0.39 to 0.57) | −0.36 (−0.43 to −0.29) |
| Mozambique | 58 (48 to 70) | 0.60 (0.47 to 0.75) | 110 (91 to 133) | 0.53 (0.42 to 0.65) | −0.50 (−0.57 to −0.43) |
| Myanmar | 167 (139 to 198) | 0.50 (0.41 to 0.61) | 230 (190 to 281) | 0.43 (0.36 to 0.52) | −0.56 (−0.64 to −0.48) |
| Namibia | 5 (4 to 5) | 0.43 (0.35 to 0.54) | 8 (6 to 9) | 0.39 (0.32 to 0.48) | −0.38 (−0.45 to −0.31) |
| Nepal | 70 (58 to 84) | 0.42 (0.34 to 0.52) | 108 (88 to 132) | 0.38 (0.32 to 0.47) | −0.29 (−0.35 to −0.22) |
| Netherlands | 383 (363 to 405) | 2.10 (1.99 to 2.22) | 759 (720 to 800) | 2.45 (2.31 to 2.59) | 0.54 (0.51 to 0.58) |
| New Zealand | 86 (82 to 91) | 2.26 (2.14 to 2.39) | 204 (195 to 215) | 2.62 (2.50 to 2.76) | 0.55 (0.51 to 0.59) |
| Nicaragua | 14 (12 to 17) | 0.47 (0.39 to 0.56) | 26 (21 to 30) | 0.44 (0.37 to 0.51) | −0.22 (−0.28 to −0.17) |
| Niger | 29 (24 to 35) | 0.48 (0.39 to 0.59) | 83 (69 to 99) | 0.46 (0.37 to 0.56) | −0.17 (−0.24 to −0.10) |
| Nigeria | 282 (231 to 344) | 0.38 (0.31 to 0.48) | 600 (489 to 736) | 0.33 (0.27 to 0.41) | −0.58 (−0.70 to −0.47) |
| North Korea | 142 (121 to 165) | 0.72 (0.61 to 0.83) | 177 (148 to 208) | 0.64 (0.54 to 0.74) | −0.46 (−0.55 to −0.37) |
| Northern Mariana Islands | 0 (0 to 0) | 0.38 (0.31 to 0.46) | 0 (0 to 0) | 0.36 (0.29 to 0.43) | −0.16 (−0.22 to −0.11) |
| Norway | 122 (115 to 130) | 2.09 (1.97 to 2.23) | 198 (186 to 209) | 2.28 (2.15 to 2.42) | 0.31 (0.29 to 0.34) |
| Oman | 8 (6 to 9) | 0.48 (0.40 to 0.56) | 18 (15 to 22) | 0.50 (0.43 to 0.58) | 0.18 (0.12 to 0.23) |
| Pakistan | 409 (337 to 494) | 0.43 (0.35 to 0.52) | 794 (652 to 958) | 0.39 (0.32 to 0.46) | −0.30 (−0.38 to −0.22) |
| Palestine | 10 (8 to 12) | 0.54 (0.44 to 0.66) | 21 (18 to 25) | 0.49 (0.41 to 0.59) | −0.28 (−0.35 to −0.22) |
| Panama | 9 (7 to 10) | 0.44 (0.37 to 0.52) | 21 (18 to 24) | 0.49 (0.42 to 0.55) | 0.30 (0.27 to 0.33) |
| Papua New Guinea | 14 (11 to 16) | 0.41 (0.34 to 0.48) | 34 (28 to 40) | 0.37 (0.31 to 0.45) | −0.32 (−0.38 to −0.26) |
| Paraguay | 20 (17 to 23) | 0.61 (0.51 to 0.72) | 35 (30 to 41) | 0.56 (0.47 to 0.64) | −0.32 (−0.41 to −0.24) |
| Peru | 71 (60 to 82) | 0.41 (0.34 to 0.49) | 147 (125 to 168) | 0.42 (0.36 to 0.48) | 0.13 (0.09 to 0.16) |
| Philippines | 207 (169 to 250) | 0.39 (0.32 to 0.49) | 349 (289 to 424) | 0.34 (0.28 to 0.41) | −0.51 (−0.61 to −0.41) |
| Poland | 273 (235 to 315) | 0.70 (0.61 to 0.80) | 545 (504 to 589) | 0.99 (0.91 to 1.07) | 1.25 (1.16 to 1.35) |
| Portugal | 128 (116 to 142) | 1.10 (0.99 to 1.20) | 291 (275 to 307) | 1.57 (1.47 to 1.67) | 1.28 (1.17 to 1.38) |
| Principality of Monaco | 0 (0 to 0) | 0.82 (0.70 to 0.93) | 1 (0 to 1) | 0.83 (0.73 to 0.92) | 0.07 (0.02 to 0.12) |
| Puerto Rico | 20 (17 to 23) | 0.57 (0.49 to 0.65) | 44 (40 to 47) | 0.83 (0.76 to 0.89) | 1.44 (1.36 to 1.53) |
| Qatar | 1 (1 to 2) | 0.40 (0.33 to 0.49) | 10 (8 to 13) | 0.38 (0.31 to 0.46) | −0.21 (−0.26 to −0.16) |
| Republic of Nauru | 0 (0 to 0) | 0.43 (0.36 to 0.49) | 0 (0 to 0) | 0.38 (0.32 to 0.44) | −0.49 (−0.55 to −0.43) |
| Republic of Niue | 0 (0 to 0) | 0.51 (0.43 to 0.59) | 0 (0 to 0) | 0.44 (0.38 to 0.51) | −0.57 (−0.63 to −0.51) |
| Republic of Palau | 0 (0 to 0) | 0.51 (0.43 to 0.58) | 0 (0 to 0) | 0.41 (0.35 to 0.48) | −0.79 (−0.85 to −0.72) |
| Republic of San Marino | 0 (0 to 0) | 0.90 (0.74 to 1.06) | 0 (0 to 0) | 0.74 (0.62 to 0.87) | −0.71 (−0.84 to −0.58) |
| Romania | 158 (135 to 182) | 0.69 (0.60 to 0.78) | 126 (106 to 146) | 0.57 (0.49 to 0.65) | −0.80 (−0.91 to −0.70) |
| Russian Federation | 872 (724 to 1051) | 0.56 (0.47 to 0.67) | 1267 (1124 to 1412) | 0.69 (0.60 to 0.76) | 0.91 (0.82 to 1.01) |
| Rwanda | 22 (18 to 27) | 0.45 (0.36 to 0.56) | 40 (32 to 50) | 0.41 (0.33 to 0.51) | −0.37 (−0.44 to −0.30) |
| Saint Kitts and Nevis | 0 (0 to 0) | 0.59 (0.50 to 0.68) | 1 (1 to 1) | 0.88 (0.80 to 0.95) | 1.45 (1.38 to 1.52) |
| Saint Lucia | 1 (1 to 1) | 0.55 (0.47 to 0.63) | 2 (2 to 2) | 0.83 (0.76 to 0.90) | 1.55 (1.47 to 1.63) |
| Saint Vincent and the Grenadines | 0 (0 to 1) | 0.54 (0.46 to 0.63) | 1 (1 to 1) | 0.66 (0.59 to 0.73) | 0.83 (0.77 to 0.89) |
| Samoa | 1 (1 to 1) | 0.47 (0.40 to 0.55) | 1 (1 to 1) | 0.41 (0.35 to 0.47) | −0.53 (−0.59 to −0.47) |
| Sao Tome and Principe | 0 (0 to 0) | 0.41 (0.35 to 0.48) | 1 (1 to 1) | 0.54 (0.48 to 0.61) | 1.14 (1.07 to 1.21) |
| Saudi Arabia | 58 (47 to 70) | 0.44 (0.36 to 0.54) | 136 (107 to 171) | 0.41 (0.34 to 0.49) | −0.30 (−0.37 to −0.23) |
| Senegal | 24 (20 to 29) | 0.42 (0.34 to 0.52) | 48 (39 to 58) | 0.39 (0.31 to 0.48) | −0.28 (−0.35 to −0.21) |
| Serbia | 58 (48 to 70) | 0.57 (0.47 to 0.67) | 55 (45 to 67) | 0.52 (0.43 to 0.61) | −0.39 (−0.47 to −0.31) |
| Seychelles | 0 (0 to 0) | 0.42 (0.35 to 0.48) | 1 (0 to 1) | 0.46 (0.40 to 0.52) | 0.38 (0.33 to 0.43) |
| Sierra Leone | 14 (11 to 17) | 0.42 (0.34 to 0.52) | 26 (22 to 32) | 0.39 (0.32 to 0.48) | −0.21 (−0.29 to −0.14) |
| Singapore | 13 (11 to 15) | 0.54 (0.47 to 0.60) | 40 (35 to 44) | 0.53 (0.47 to 0.58) | −0.03 (−0.05 to −0.01) |
| Slovakia | 30 (25 to 36) | 0.56 (0.47 to 0.66) | 35 (29 to 40) | 0.53 (0.45 to 0.61) | −0.16 (−0.23 to −0.10) |
| Slovenia | 14 (12 to 16) | 0.70 (0.62 to 0.79) | 14 (11 to 17) | 0.51 (0.42 to 0.60) | −1.39 (−1.59 to −1.18) |
| Solomon Islands | 1 (1 to 1) | 0.45 (0.38 to 0.54) | 2 (2 to 3) | 0.41 (0.34 to 0.48) | −0.37 (−0.44 to −0.31) |
| Somalia | 29 (23 to 35) | 0.55 (0.44 to 0.70) | 73 (59 to 90) | 0.53 (0.41 to 0.66) | −0.21 (−0.28 to −0.14) |
| South Africa | 138 (113 to 170) | 0.45 (0.37 to 0.57) | 213 (170 to 270) | 0.40 (0.32 to 0.50) | −0.47 (−0.58 to −0.36) |
| South Korea | 250 (210 to 291) | 0.72 (0.61 to 0.82) | 396 (323 to 471) | 0.57 (0.49 to 0.66) | −0.91 (−1.00 to −0.81) |
| South Sudan | 19 (16 to 23) | 0.47 (0.37 to 0.59) | 31 (26 to 38) | 0.46 (0.37 to 0.57) | −0.17 (−0.24 to −0.09) |
| Spain | 621 (579 to 669) | 1.29 (1.20 to 1.39) | 1333 (1263 to 1408) | 1.69 (1.59 to 1.78) | 0.86 (0.76 to 0.97) |
| Sri Lanka | 54 (45 to 65) | 0.38 (0.31 to 0.45) | 82 (68 to 97) | 0.34 (0.29 to 0.40) | −0.32 (−0.35 to −0.28) |
| Sudan | 85 (70 to 102) | 0.46 (0.38 to 0.56) | 155 (127 to 185) | 0.42 (0.35 to 0.50) | −0.38 (−0.45 to −0.31) |
| Suriname | 2 (1 to 2) | 0.49 (0.41 to 0.56) | 4 (3 to 4) | 0.60 (0.54 to 0.66) | 0.78 (0.74 to 0.83) |
| Swaziland | 3 (2 to 3) | 0.48 (0.39 to 0.60) | 4 (3 to 5) | 0.44 (0.36 to 0.53) | −0.36 (−0.43 to −0.29) |
| Sweden | 271 (256 to 288) | 2.07 (1.94 to 2.22) | 449 (426 to 472) | 2.41 (2.28 to 2.55) | 0.55 (0.48 to 0.63) |
| Switzerland | 179 (169 to 191) | 1.92 (1.80 to 2.05) | 287 (270 to 303) | 1.88 (1.77 to 2.01) | −0.08 (−0.11 to −0.05) |
| Syria | 59 (50 to 71) | 0.54 (0.44 to 0.65) | 66 (54 to 80) | 0.49 (0.40 to 0.59) | −0.33 (−0.40 to −0.26) |
| Taiwan (Province of China) | 92 (79 to 106) | 0.53 (0.45 to 0.60) | 198 (176 to 220) | 0.61 (0.54 to 0.68) | −0.30 (−0.67 to 0.06) |
| Tajikistan | 22 (18 to 26) | 0.46 (0.38 to 0.57) | 40 (32 to 48) | 0.43 (0.36 to 0.53) | −0.25 (−0.32 to −0.19) |
| Tanzania | 83 (68 to 100) | 0.46 (0.37 to 0.58) | 175 (142 to 211) | 0.42 (0.33 to 0.52) | −0.42 (−0.49 to −0.34) |
| Thailand | 186 (152 to 229) | 0.40 (0.33 to 0.49) | 292 (232 to 365) | 0.36 (0.29 to 0.44) | −0.39 (−0.46 to −0.32) |
| The Bahamas | 1 (1 to 1) | 0.58 (0.49 to 0.67) | 3 (2 to 3) | 0.69 (0.61 to 0.76) | 0.68 (0.64 to 0.72) |
| The Gambia | 3 (2 to 4) | 0.43 (0.35 to 0.52) | 7 (6 to 8) | 0.40 (0.32 to 0.48) | −0.27 (−0.33 to −0.20) |
| Timor-Leste | 3 (2 to 3) | 0.40 (0.32 to 0.48) | 4 (4 to 5) | 0.35 (0.29 to 0.42) | −0.45 (−0.53 to −0.36) |
| Togo | 11 (9 to 13) | 0.41 (0.32 to 0.50) | 25 (20 to 30) | 0.38 (0.31 to 0.47) | −0.25 (−0.32 to −0.19) |
| Tokelau | 0 (0 to 0) | 0.43 (0.36 to 0.50) | 0 (0 to 0) | 0.37 (0.31 to 0.44) | −0.53 (−0.59 to −0.47) |
| Tonga | 0 (0 to 0) | 0.46 (0.39 to 0.54) | 0 (0 to 0) | 0.42 (0.35 to 0.48) | −0.40 (−0.47 to −0.34) |
| Trinidad and Tobago | 5 (4 to 6) | 0.51 (0.42 to 0.59) | 11 (9 to 12) | 0.63 (0.55 to 0.69) | 0.73 (0.64 to 0.82) |
| Tunisia | 37 (31 to 44) | 0.51 (0.42 to 0.61) | 55 (45 to 67) | 0.45 (0.38 to 0.54) | −0.37 (−0.43 to −0.30) |
| Turkey | 422 (384 to 462) | 0.84 (0.76 to 0.92) | 815 (747 to 888) | 0.95 (0.88 to 1.03) | 0.48 (0.46 to 0.51) |
| Turkmenistan | 14 (12 to 17) | 0.44 (0.36 to 0.52) | 21 (17 to 25) | 0.42 (0.36 to 0.50) | −0.12 (−0.16 to −0.08) |
| Tuvalu | 0 (0 to 0) | 0.45 (0.38 to 0.53) | 0 (0 to 0) | 0.39 (0.33 to 0.46) | −0.56 (−0.62 to −0.50) |
| Uganda | 52 (43 to 63) | 0.45 (0.36 to 0.56) | 113 (93 to 135) | 0.39 (0.31 to 0.49) | −0.47 (−0.54 to −0.40) |
| Ukraine | 293 (242 to 354) | 0.52 (0.43 to 0.62) | 253 (211 to 298) | 0.50 (0.42 to 0.58) | −0.09 (−0.15 to −0.02) |
| United Arab Emirates | 6 (5 to 8) | 0.42 (0.34 to 0.50) | 41 (32 to 53) | 0.42 (0.36 to 0.49) | 0.08 (0.05 to 0.12) |
| United Kingdom | 1650 (1561 to 1746) | 2.06 (1.95 to 2.19) | 3120 (2949 to 3297) | 2.66 (2.52 to 2.82) | 0.94 (0.89 to 0.99) |
| United States | 5281 (5004 to 5604) | 1.79 (1.69 to 1.91) | 11089 (10634 to 11559) | 2.06 (1.98 to 2.14) | 0.57 (0.52 to 0.61) |
| Uruguay | 30 (27 to 34) | 0.86 (0.75 to 0.96) | 65 (61 to 69) | 1.37 (1.28 to 1.46) | 1.49 (1.34 to 1.63) |
| Uzbekistan | 89 (74 to 106) | 0.49 (0.40 to 0.61) | 145 (120 to 178) | 0.44 (0.36 to 0.54) | −0.37 (−0.43 to −0.32) |
| Vanuatu | 1 (0 to 1) | 0.48 (0.40 to 0.56) | 1 (1 to 1) | 0.43 (0.36 to 0.51) | −0.38 (−0.44 to −0.31) |
| Venezuela | 67 (56 to 77) | 0.46 (0.38 to 0.53) | 159 (140 to 177) | 0.54 (0.48 to 0.60) | 0.66 (0.63 to 0.69) |
| Vietnam | 239 (198 to 279) | 0.42 (0.34 to 0.50) | 385 (319 to 455) | 0.38 (0.32 to 0.44) | −0.36 (−0.42 to −0.30) |
| Virgin Islands, U.S. | 1 (1 to 1) | 0.75 (0.66 to 0.83) | 2 (2 to 2) | 1.18 (1.10 to 1.27) | 1.64 (1.49 to 1.78) |
| Yemen | 58 (48 to 70) | 0.47 (0.38 to 0.56) | 125 (104 to 151) | 0.43 (0.36 to 0.51) | −0.30 (−0.37 to −0.22) |
| Zambia | 27 (23 to 33) | 0.52 (0.41 to 0.65) | 62 (51 to 75) | 0.46 (0.37 to 0.57) | −0.47 (−0.55 to −0.40) |
| Zimbabwe | 32 (27 to 39) | 0.43 (0.36 to 0.52) | 50 (42 to 60) | 0.43 (0.36 to 0.50) | 0 (−0.04 to 0.04) |

Abbreviations: ASR, age-standardized rate; MND, motor neuron disease; EAPC, estimated annual percentage change; SDI, sociodemographic index; UI, uncertainty interval; CI, confidence interval.

**Table S5.** The case number and ASR of prevalence of MND in 1990 and 2021, with EAPC from 1990 to 2021.

|  | **1990** | | **2021** | | **EAPC (95% CI)** |
| --- | --- | --- | --- | --- | --- |
|  | **Number (95% UI)** | **ASR (95% UI)** | **Number (95% UI)** | **ASR (95% UI)** |  |
| Sex |  |  |  |  |  |
| Female | 77382 (65287 to 90853) | 3.13 (2.66 to 3.66) | 127253 (109471 to 146510) | 3.02 (2.60 to 3.52) | 0.02 (−0.04 to 0.09) |
| Male | 84544 (71637 to 98825) | 3.61 (3.11 to 4.21) | 145479 (126364 to 166787) | 3.64 (3.16 to 4.16) | 0.19 (0.11 to 0.28) |
| Age |  |  |  |  |  |
| <5 years | 6256 (4709 to 8166) | 1.01 (0.76 to 1.32) | 6094 (4429 to 8181) | 0.93 (0.67 to 1.24) | −0.21 (−0.26 to −0.16) |
| 5-9 years | 9531 (7166 to 12417) | 1.63 (1.23 to 2.13) | 10748 (7975 to 14197) | 1.56 (1.16 to 2.07) | −0.12 (−0.15 to −0.08) |
| 10-14 years | 11116 (8005 to 14729) | 2.08 (1.49 to 2.75) | 13012 (9252 to 17459) | 1.95 (1.39 to 2.62) | −0.21 (−0.26 to −0.16) |
| 15-19 years | 12584 (9017 to 16993) | 2.42 (1.74 to 3.27) | 13911 (9938 to 18772) | 2.23 (1.59 to 3.01) | −0.26 (−0.31 to −0.20) |
| 20-24 years | 13192 (9306 to 17892) | 2.68 (1.89 to 3.64) | 14669 (10341 to 19928) | 2.46 (1.73 to 3.34) | −0.22 (−0.27 to −0.18) |
| 25-29 years | 12941 (9152 to 17965) | 2.92 (2.07 to 4.06) | 15772 (11185 to 21916) | 2.68 (1.90 to 3.72) | −0.19 (−0.22 to −0.15) |
| 30-34 years | 11580 (8125 to 16132) | 3.00 (2.11 to 4.19) | 16927 (11860 to 23398) | 2.80 (1.96 to 3.87) | −0.17 (−0.22 to −0.12) |
| 35-39 years | 10217 (6679 to 14993) | 2.90 (1.90 to 4.26) | 15269 (10207 to 22038) | 2.72 (1.82 to 3.93) | −0.15 (−0.21 to −0.10) |
| 40-44 years | 8278 (5062 to 12564) | 2.89 (1.77 to 4.39) | 13311 (8465 to 19690) | 2.66 (1.69 to 3.94) | −0.14 (−0.24 to −0.04) |
| 45-49 years | 6866 (4167 to 10367) | 2.96 (1.79 to 4.46) | 12999 (8246 to 19103) | 2.75 (1.74 to 4.03) | −0.09 (−0.20 to 0.02) |
| 50-54 years | 7219 (4413 to 10432) | 3.40 (2.08 to 4.91) | 14510 (9382 to 20454) | 3.26 (2.11 to 4.60) | 0.04 (−0.10 to 0.18) |
| 55-59 years | 8237 (5443 to 11426) | 4.45 (2.94 to 6.17) | 17437 (12230 to 23135) | 4.41 (3.09 to 5.85) | 0.24 (0.07 to 0.41) |
| 60-64 years | 10178 (7221 to 13968) | 6.34 (4.50 to 8.70) | 20677 (15507 to 27391) | 6.46 (4.85 to 8.56) | 0.31 (0.13 to 0.49) |
| 65-69 years | 11238 (8270 to 14893) | 9.09 (6.69 to 12.05) | 24174 (18401 to 31811) | 8.76 (6.67 to 11.53) | 0.36 (0.21 to 0.52) |
| 70-74 years | 8825 (6450 to 11715) | 10.42 (7.62 to 13.84) | 25047 (19080 to 32499) | 12.17 (9.27 to 15.79) | 0.64 (0.60 to 0.69) |
| 75-79 years | 7292 (5421 to 9569) | 11.85 (8.81 to 15.55) | 17922 (14150 to 22728) | 13.59 (10.73 to 17.23) | 0.66 (0.53 to 0.79) |
| 80-84 years | 4152 (2971 to 5473) | 11.74 (8.40 to 15.47) | 11674 (8830 to 14965) | 13.33 (10.08 to 17.09) | 0.60 (0.46 to 0.75) |
| 85-89 years | 1711 (1183 to 2328) | 11.33 (7.83 to 15.41) | 5875 (4146 to 7782) | 12.85 (9.07 to 17.02) | 0.69 (0.58 to 0.80) |
| 90-94 years | 411 (270 to 589) | 9.60 (6.30 to 13.75) | 2079 (1449 to 2879) | 11.62 (8.10 to 16.10) | 0.97 (0.87 to 1.08) |
| 95+ years | 100 (57 to 155) | 9.84 (5.61 to 15.19) | 625 (380 to 924) | 11.47 (6.96 to 16.95) | 0.80 (0.66 to 0.94) |
| **Country** |  |  |  |  |  |
| Afghanistan | 178 (145 to 219) | 1.92 (1.56 to 2.35) | 553 (442 to 688) | 1.83 (1.49 to 2.25) | −0.06 (−0.12 to 0) |
| Albania | 93 (75 to 113) | 2.85 (2.32 to 3.44) | 82 (68 to 98) | 2.94 (2.40 to 3.54) | 0.17 (0.12 to 0.21) |
| Algeria | 608 (481 to 747) | 2.49 (2.03 to 3.02) | 1072 (855 to 1308) | 2.43 (1.94 to 2.96) | −0.04 (−0.08 to −0.01) |
| American Samoa | 1 (1 to 1) | 2.06 (1.66 to 2.50) | 1 (1 to 1) | 1.83 (1.45 to 2.22) | −0.43 (−0.48 to −0.39) |
| Andorra | 5 (5 to 6) | 9.21 (7.90 to 10.81) | 14 (12 to 17) | 10.89 (9.49 to 12.68) | 0.63 (0.59 to 0.67) |
| Angola | 121 (95 to 153) | 1.37 (1.07 to 1.71) | 388 (298 to 495) | 1.35 (1.06 to 1.69) | 0.03 (−0.01 to 0.06) |
| Antigua and Barbuda | 2 (1 to 2) | 2.67 (2.15 to 3.21) | 3 (2 to 3) | 3 (2.48 to 3.54) | 0.42 (0.40 to 0.44) |
| Argentina | 1214 (1008 to 1433) | 3.69 (3.06 to 4.36) | 2275 (1931 to 2637) | 4.66 (3.95 to 5.41) | 0.82 (0.77 to 0.86) |
| Armenia | 92 (73 to 113) | 2.67 (2.15 to 3.27) | 80 (65 to 98) | 2.66 (2.14 to 3.22) | 0.07 (0.01 to 0.13) |
| Australia | 1244 (1078 to 1430) | 6.72 (5.86 to 7.69) | 3285 (2822 to 3793) | 8.98 (7.77 to 10.16) | 1.04 (0.96 to 1.13) |
| Austria | 631 (541 to 729) | 6.76 (5.77 to 7.75) | 1151 (976 to 1342) | 8.73 (7.48 to 10.01) | 0.88 (0.79 to 0.98) |
| Azerbaijan | 181 (144 to 222) | 2.47 (1.98 to 3.03) | 262 (209 to 320) | 2.45 (1.97 to 2.98) | 0.08 (−0.01 to 0.17) |
| Bahrain | 14 (11 to 18) | 2.76 (2.19 to 3.32) | 44 (35 to 55) | 2.74 (2.19 to 3.33) | 0.02 (−0.02 to 0.05) |
| Bangladesh | 1532 (1185 to 1940) | 1.49 (1.18 to 1.84) | 2608 (2045 to 3248) | 1.56 (1.24 to 1.93) | 0.14 (0.10 to 0.18) |
| Barbados | 7 (6 to 9) | 2.79 (2.28 to 3.32) | 13 (11 to 15) | 3.66 (3.07 to 4.25) | 0.87 (0.77 to 0.97) |
| Belarus | 411 (339 to 487) | 3.86 (3.17 to 4.57) | 443 (375 to 516) | 4.29 (3.57 to 5.01) | 0.43 (0.39 to 0.46) |
| Belgium | 1232 (1049 to 1429) | 9.28 (8.02 to 10.63) | 1836 (1557 to 2130) | 10.22 (8.77 to 11.77) | 0.31 (0.28 to 0.35) |
| Belize | 4 (3 to 5) | 2.23 (1.81 to 2.69) | 10 (8 to 12) | 2.29 (1.86 to 2.74) | 0.09 (0.06 to 0.12) |
| Benin | 52 (40 to 66) | 1.23 (0.95 to 1.53) | 147 (112 to 188) | 1.21 (0.95 to 1.50) | −0.04 (−0.08 to 0) |
| Bermuda | 3 (3 to 4) | 5.26 (4.49 to 6.11) | 4 (4 to 5) | 5.50 (4.71 to 6.33) | 0.16 (0.14 to 0.17) |
| Bhutan | 10 (8 to 13) | 1.67 (1.32 to 2.06) | 14 (11 to 18) | 1.79 (1.42 to 2.20) | 0.21 (0.18 to 0.24) |
| Bolivia | 106 (84 to 130) | 1.83 (1.48 to 2.20) | 234 (192 to 280) | 2.05 (1.70 to 2.44) | 0.46 (0.43 to 0.50) |
| Bosnia and Herzegovina | 153 (125 to 182) | 3.35 (2.76 to 3.96) | 117 (96 to 142) | 3.55 (2.86 to 4.27) | 0.24 (0.14 to 0.34) |
| Botswana | 20 (16 to 26) | 1.64 (1.30 to 2.03) | 40 (31 to 50) | 1.66 (1.32 to 2.04) | 0.02 (−0.01 to 0.06) |
| Brazil | 3686 (2916 to 4523) | 2.52 (2.03 to 3.04) | 6628 (5532 to 7769) | 2.89 (2.40 to 3.39) | 0.57 (0.54 to 0.60) |
| Brunei | 5 (4 to 6) | 2.22 (1.82 to 2.71) | 10 (8 to 13) | 2.33 (1.94 to 2.80) | 0.26 (0.22 to 0.30) |
| Bulgaria | 271 (222 to 328) | 3.15 (2.59 to 3.78) | 209 (172 to 252) | 3.11 (2.54 to 3.75) | −0.03 (−0.07 to 0.02) |
| Burkina Faso | 100 (78 to 126) | 1.21 (0.94 to 1.50) | 244 (188 to 314) | 1.19 (0.94 to 1.49) | −0.02 (−0.06 to 0.02) |
| Burundi | 53 (41 to 68) | 1.10 (0.86 to 1.38) | 123 (94 to 158) | 1.04 (0.81 to 1.31) | −0.19 (−0.23 to −0.15) |
| Cambodia | 129 (100 to 161) | 1.32 (1.05 to 1.62) | 233 (183 to 290) | 1.37 (1.09 to 1.68) | 0.13 (0.09 to 0.17) |
| Cameroon | 108 (83 to 137) | 1.17 (0.91 to 1.45) | 332 (253 to 426) | 1.12 (0.88 to 1.39) | −0.12 (−0.16 to −0.08) |
| Canada | 3277 (2883 to 3765) | 11.07 (9.77 to 12.63) | 6929 (5946 to 7933) | 12.76 (11.07 to 14.42) | 0.52 (0.48 to 0.57) |
| Cape Verde | 5 (4 to 7) | 1.63 (1.29 to 2.02) | 9 (7 to 12) | 1.64 (1.29 to 2.02) | 0.03 (0 to 0.07) |
| Central African Republic | 26 (20 to 33) | 1.07 (0.83 to 1.33) | 50 (38 to 65) | 1.00 (0.77 to 1.25) | −0.22 (−0.26 to −0.18) |
| Chad | 66 (52 to 84) | 1.27 (0.99 to 1.57) | 194 (149 to 249) | 1.26 (0.98 to 1.56) | 0.02 (−0.02 to 0.05) |
| Chile | 549 (456 to 644) | 4.32 (3.63 to 5.05) | 1204 (1027 to 1396) | 5.67 (4.82 to 6.54) | 0.96 (0.90 to 1.01) |
| China | 25692 (20345 to 31960) | 2.13 (1.72 to 2.60) | 33342 (27028 to 40366) | 2.30 (1.84 to 2.80) | 0.28 (0.22 to 0.35) |
| Colombia | 594 (470 to 735) | 1.90 (1.53 to 2.29) | 1191 (990 to 1394) | 2.33 (1.95 to 2.74) | 0.81 (0.76 to 0.85) |
| Comoros | 6 (5 to 7) | 1.46 (1.15 to 1.82) | 10 (8 to 13) | 1.39 (1.09 to 1.73) | −0.17 (−0.21 to −0.13) |
| Congo | 25 (19 to 31) | 1.13 (0.89 to 1.42) | 57 (44 to 73) | 1.10 (0.85 to 1.38) | −0.07 (−0.10 to −0.03) |
| Cook Islands | 0 (0 to 1) | 2.36 (1.89 to 2.83) | 0 (0 to 0) | 2.27 (1.82 to 2.74) | −0.19 (−0.23 to −0.15) |
| Costa Rica | 71 (57 to 88) | 2.45 (2.00 to 2.93) | 179 (152 to 208) | 3.51 (3.00 to 4.09) | 1.20 (1.13 to 1.26) |
| Cote d'Ivoire | 133 (101 to 170) | 1.23 (0.96 to 1.53) | 308 (235 to 394) | 1.19 (0.93 to 1.48) | −0.11 (−0.16 to −0.07) |
| Croatia | 212 (179 to 247) | 4.31 (3.66 to 5.02) | 217 (184 to 250) | 4.64 (3.93 to 5.46) | 0.34 (0.30 to 0.38) |
| Cuba | 317 (256 to 379) | 2.89 (2.33 to 3.43) | 414 (353 to 478) | 3.35 (2.85 to 3.91) | 0.57 (0.54 to 0.59) |
| Cyprus | 46 (39 to 54) | 5.58 (4.76 to 6.50) | 112 (96 to 131) | 6.38 (5.46 to 7.37) | 0.49 (0.43 to 0.55) |
| Czech Republic | 470 (390 to 551) | 4.51 (3.74 to 5.33) | 546 (463 to 638) | 4.73 (3.98 to 5.55) | 0.21 (0.18 to 0.24) |
| Democratic Republic of the Congo | 370 (283 to 470) | 1.12 (0.87 to 1.40) | 847 (645 to 1094) | 1.03 (0.80 to 1.29) | −0.26 (−0.33 to −0.20) |
| Denmark | 622 (530 to 720) | 9.26 (7.83 to 10.67) | 966 (825 to 1125) | 10.56 (9.05 to 12.20) | 0.45 (0.38 to 0.51) |
| Djibouti | 6 (4 to 7) | 1.49 (1.17 to 1.84) | 17 (14 to 22) | 1.43 (1.13 to 1.78) | −0.16 (−0.21 to −0.11) |
| Dominica | 2 (1 to 2) | 2.35 (1.89 to 2.81) | 2 (2 to 2) | 2.95 (2.47 to 3.41) | 0.82 (0.77 to 0.87) |
| Dominican Republic | 154 (121 to 189) | 2.25 (1.82 to 2.73) | 282 (232 to 335) | 2.57 (2.13 to 3.03) | 0.55 (0.50 to 0.60) |
| Ecuador | 145 (113 to 182) | 1.55 (1.23 to 1.90) | 324 (263 to 387) | 1.82 (1.48 to 2.17) | 0.72 (0.66 to 0.78) |
| Egypt | 1153 (919 to 1414) | 2.13 (1.72 to 2.59) | 2268 (1800 to 2762) | 2.16 (1.73 to 2.62) | 0.06 (0.03 to 0.09) |
| El Salvador | 97 (76 to 119) | 1.89 (1.52 to 2.31) | 124 (99 to 150) | 1.90 (1.54 to 2.31) | 0.02 (−0.01 to 0.06) |
| Equatorial Guinea | 4 (3 to 5) | 1.11 (0.86 to 1.39) | 17 (13 to 23) | 1.22 (0.96 to 1.54) | 0.47 (0.36 to 0.58) |
| Eritrea | 41 (31 to 52) | 1.36 (1.07 to 1.70) | 83 (63 to 106) | 1.34 (1.05 to 1.65) | −0.11 (−0.16 to −0.05) |
| Estonia | 63 (52 to 75) | 4.02 (3.29 to 4.79) | 54 (45 to 63) | 4.08 (3.34 to 4.81) | 0.09 (0.05 to 0.13) |
| Ethiopia | 549 (408 to 714) | 1.24 (0.94 to 1.56) | 1238 (910 to 1630) | 1.20 (0.91 to 1.53) | −0.08 (−0.17 to 0) |
| Federated States of Micronesia | 1 (1 to 2) | 1.39 (1.13 to 1.69) | 1 (1 to 2) | 1.34 (1.07 to 1.66) | −0.13 (−0.17 to −0.10) |
| Fiji | 13 (11 to 17) | 1.75 (1.41 to 2.14) | 16 (13 to 19) | 1.75 (1.39 to 2.12) | −0.03 (−0.07 to 0.01) |
| Finland | 741 (634 to 859) | 11.79 (10.18 to 13.50) | 990 (833 to 1154) | 11.16 (9.46 to 12.82) | −0.24 (−0.39 to −0.09) |
| France | 6332 (5453 to 7243) | 8.95 (7.76 to 10.17) | 11408 (9997 to 13107) | 10.84 (9.52 to 12.42) | 0.64 (0.59 to 0.68) |
| Gabon | 11 (9 to 14) | 1.27 (1.01 to 1.59) | 21 (16 to 26) | 1.21 (0.95 to 1.51) | −0.16 (−0.19 to −0.12) |
| Georgia | 151 (123 to 183) | 2.75 (2.23 to 3.33) | 98 (81 to 117) | 2.75 (2.22 to 3.27) | 0.14 (0.05 to 0.24) |
| Germany | 7226 (6175 to 8443) | 7.14 (6.13 to 8.29) | 12379 (10700 to 14387) | 8.90 (7.75 to 10.17) | 0.79 (0.74 to 0.84) |
| Ghana | 166 (127 to 209) | 1.22 (0.95 to 1.52) | 400 (309 to 513) | 1.22 (0.97 to 1.52) | −0.01 (−0.05 to 0.03) |
| Greece | 566 (479 to 668) | 4.64 (3.93 to 5.44) | 1032 (892 to 1195) | 6.74 (5.82 to 7.74) | 1.45 (1.32 to 1.57) |
| Greenland | 4 (4 to 5) | 8.95 (7.70 to 10.39) | 6 (5 to 7) | 9.36 (8.04 to 10.88) | 0.21 (0.15 to 0.26) |
| Grenada | 2 (1 to 2) | 2.14 (1.71 to 2.57) | 2 (2 to 3) | 2.33 (1.91 to 2.80) | 0.24 (0.18 to 0.30) |
| Guam | 3 (3 to 4) | 2.80 (2.32 to 3.27) | 3 (3 to 4) | 2.00 (1.60 to 2.42) | −1.39 (−1.54 to −1.23) |
| Guatemala | 139 (109 to 170) | 1.79 (1.45 to 2.17) | 280 (223 to 345) | 1.79 (1.45 to 2.17) | 0 (−0.04 to 0.04) |
| Guinea | 64 (50 to 80) | 1.20 (0.93 to 1.50) | 141 (108 to 181) | 1.16 (0.90 to 1.45) | −0.15 (−0.19 to −0.10) |
| Guinea-Bissau | 11 (8 to 13) | 1.16 (0.91 to 1.44) | 22 (16 to 28) | 1.12 (0.88 to 1.39) | −0.15 (−0.19 to −0.11) |
| Guyana | 12 (9 to 15) | 1.59 (1.26 to 1.94) | 14 (11 to 17) | 1.84 (1.51 to 2.19) | 0.51 (0.48 to 0.53) |
| Haiti | 103 (82 to 128) | 1.72 (1.40 to 2.08) | 209 (168 to 255) | 1.69 (1.39 to 2.03) | −0.03 (−0.07 to 0) |
| Honduras | 84 (66 to 102) | 1.89 (1.55 to 2.27) | 200 (160 to 243) | 2.07 (1.71 to 2.47) | 0.38 (0.35 to 0.41) |
| Hungary | 407 (342 to 478) | 3.86 (3.24 to 4.54) | 466 (401 to 537) | 4.57 (3.91 to 5.31) | 0.66 (0.59 to 0.73) |
| Iceland | 28 (24 to 32) | 10.28 (8.85 to 11.77) | 57 (49 to 66) | 12.03 (10.48 to 13.80) | 0.57 (0.53 to 0.61) |
| India | 12434 (9448 to 15840) | 1.47 (1.13 to 1.84) | 22870 (17756 to 28955) | 1.57 (1.23 to 1.96) | 0.23 (0.17 to 0.29) |
| Indonesia | 2518 (1895 to 3221) | 1.35 (1.04 to 1.70) | 3771 (2899 to 4777) | 1.33 (1.04 to 1.68) | −0.10 (−0.16 to −0.03) |
| Iran | 1553 (1231 to 1907) | 2.88 (2.29 to 3.54) | 2478 (1936 to 3045) | 2.80 (2.22 to 3.40) | −0.05 (−0.10 to 0) |
| Iraq | 460 (364 to 571) | 2.59 (2.09 to 3.17) | 1057 (851 to 1287) | 2.54 (2.08 to 3.08) | 0.04 (−0.01 to 0.09) |
| Ireland | 387 (335 to 446) | 9.93 (8.65 to 11.34) | 927 (780 to 1078) | 13.67 (11.65 to 15.54) | 1.16 (1.12 to 1.20) |
| Israel | 286 (245 to 331) | 5.85 (4.99 to 6.77) | 713 (613 to 814) | 6.65 (5.76 to 7.64) | 0.42 (0.39 to 0.46) |
| Italy | 4721 (4050 to 5474) | 6.49 (5.58 to 7.53) | 8960 (7624 to 10458) | 8.70 (7.48 to 10.02) | 0.97 (0.82 to 1.12) |
| Jamaica | 62 (50 to 76) | 2.65 (2.16 to 3.18) | 80 (66 to 95) | 2.77 (2.31 to 3.28) | 0.18 (0.16 to 0.21) |
| Japan | 6720 (5656 to 7823) | 4.62 (3.90 to 5.35) | 11753 (10063 to 13647) | 5.55 (4.77 to 6.38) | 0.63 (0.54 to 0.73) |
| Jordan | 102 (80 to 129) | 2.78 (2.24 to 3.37) | 341 (275 to 417) | 2.71 (2.22 to 3.28) | −0.03 (−0.07 to 0) |
| Kazakhstan | 475 (389 to 570) | 2.90 (2.38 to 3.47) | 538 (432 to 652) | 2.89 (2.31 to 3.49) | 0.06 (0 to 0.12) |
| Kenya | 271 (199 to 349) | 1.31 (0.99 to 1.67) | 591 (439 to 768) | 1.20 (0.91 to 1.53) | −0.34 (−0.42 to −0.26) |
| Kiribati | 1 (1 to 1) | 1.44 (1.18 to 1.72) | 2 (1 to 2) | 1.52 (1.26 to 1.80) | 0.23 (0.21 to 0.24) |
| Kuwait | 62 (49 to 75) | 3.53 (2.90 to 4.22) | 164 (131 to 197) | 3.39 (2.77 to 4.04) | −0.12 (−0.14 to −0.10) |
| Kyrgyzstan | 109 (86 to 133) | 2.45 (1.96 to 2.95) | 161 (128 to 195) | 2.34 (1.89 to 2.82) | −0.12 (−0.19 to −0.04) |
| Laos | 56 (44 to 70) | 1.43 (1.14 to 1.76) | 110 (87 to 138) | 1.51 (1.21 to 1.85) | 0.17 (0.13 to 0.21) |
| Latvia | 100 (81 to 119) | 3.79 (3.07 to 4.50) | 76 (64 to 89) | 3.94 (3.23 to 4.66) | 0.24 (0.18 to 0.30) |
| Lebanon | 89 (72 to 107) | 3.01 (2.46 to 3.60) | 168 (136 to 203) | 2.95 (2.39 to 3.54) | −0.01 (−0.05 to 0.02) |
| Lesotho | 24 (19 to 29) | 1.64 (1.31 to 2.01) | 30 (24 to 38) | 1.62 (1.28 to 2.00) | −0.05 (−0.09 to 0) |
| Liberia | 25 (19 to 32) | 1.13 (0.89 to 1.41) | 55 (41 to 70) | 1.07 (0.83 to 1.34) | −0.10 (−0.15 to −0.04) |
| Libya | 102 (80 to 127) | 2.51 (2.02 to 3.06) | 161 (128 to 197) | 2.27 (1.81 to 2.74) | −0.31 (−0.34 to −0.27) |
| Lithuania | 143 (117 to 169) | 3.88 (3.17 to 4.58) | 168 (143 to 195) | 5.21 (4.38 to 6.08) | 1.11 (1.05 to 1.17) |
| Luxembourg | 38 (33 to 44) | 8.14 (7.03 to 9.37) | 78 (66 to 90) | 9.03 (7.77 to 10.31) | 0.32 (0.24 to 0.40) |
| Macedonia | 67 (55 to 80) | 3.32 (2.76 to 3.95) | 69 (57 to 84) | 3.18 (2.57 to 3.82) | −0.13 (−0.17 to −0.10) |
| Madagascar | 160 (125 to 200) | 1.54 (1.21 to 1.90) | 383 (298 to 484) | 1.47 (1.16 to 1.83) | −0.15 (−0.19 to −0.10) |
| Malawi | 113 (87 to 145) | 1.33 (1.05 to 1.66) | 228 (175 to 291) | 1.28 (0.99 to 1.59) | −0.13 (−0.17 to −0.09) |
| Malaysia | 278 (214 to 354) | 1.58 (1.25 to 1.97) | 524 (417 to 649) | 1.62 (1.29 to 2.00) | 0.06 (0.03 to 0.09) |
| Maldives | 3 (2 to 4) | 1.52 (1.19 to 1.86) | 9 (7 to 11) | 1.64 (1.30 to 1.99) | 0.26 (0.23 to 0.30) |
| Mali | 132 (106 to 160) | 1.96 (1.61 to 2.34) | 364 (294 to 443) | 2.03 (1.68 to 2.39) | 0.10 (0.08 to 0.13) |
| Malta | 25 (22 to 29) | 6.24 (5.38 to 7.21) | 55 (47 to 65) | 7.80 (6.74 to 8.99) | 0.77 (0.73 to 0.81) |
| Marshall Islands | 1 (0 to 1) | 1.45 (1.18 to 1.77) | 1 (1 to 1) | 1.39 (1.11 to 1.69) | −0.17 (−0.20 to −0.14) |
| Mauritania | 29 (23 to 37) | 1.58 (1.25 to 1.96) | 63 (49 to 79) | 1.54 (1.22 to 1.91) | −0.05 (−0.09 to −0.02) |
| Mauritius | 25 (20 to 30) | 2.19 (1.78 to 2.66) | 29 (24 to 35) | 2.27 (1.82 to 2.71) | 0.09 (0.05 to 0.13) |
| Mexico | 2143 (1672 to 2636) | 2.54 (2.02 to 3.07) | 3543 (2866 to 4255) | 2.71 (2.20 to 3.24) | 0.29 (0.25 to 0.33) |
| Moldova | 131 (107 to 157) | 2.95 (2.40 to 3.54) | 102 (84 to 122) | 2.89 (2.36 to 3.46) | −0.01 (−0.08 to 0.06) |
| Mongolia | 53 (42 to 65) | 2.47 (1.99 to 2.98) | 84 (67 to 101) | 2.56 (2.07 to 3.07) | 0.17 (0.12 to 0.21) |
| Montenegro | 22 (18 to 26) | 3.45 (2.82 to 4.14) | 21 (17 to 25) | 3.38 (2.77 to 4.05) | −0.01 (−0.06 to 0.04) |
| Morocco | 598 (478 to 735) | 2.39 (1.93 to 2.91) | 893 (722 to 1086) | 2.38 (1.93 to 2.89) | −0.01 (−0.05 to 0.02) |
| Mozambique | 160 (126 to 203) | 1.37 (1.09 to 1.70) | 382 (292 to 483) | 1.39 (1.09 to 1.72) | 0.06 (0.02 to 0.10) |
| Myanmar | 577 (451 to 726) | 1.45 (1.16 to 1.80) | 880 (702 to 1090) | 1.56 (1.26 to 1.93) | 0.27 (0.22 to 0.32) |
| Namibia | 22 (17 to 28) | 1.67 (1.31 to 2.05) | 39 (31 to 50) | 1.65 (1.31 to 2.03) | −0.01 (−0.05 to 0.02) |
| Nepal | 295 (231 to 371) | 1.59 (1.26 to 1.97) | 519 (407 to 646) | 1.65 (1.31 to 2.01) | 0.09 (0.04 to 0.13) |
| Netherlands | 1757 (1503 to 2024) | 9.86 (8.49 to 11.29) | 3315 (2803 to 3831) | 11.91 (10.25 to 13.54) | 0.67 (0.61 to 0.73) |
| New Zealand | 276 (236 to 320) | 7.38 (6.32 to 8.54) | 648 (555 to 751) | 9.13 (7.90 to 10.41) | 0.76 (0.68 to 0.83) |
| Nicaragua | 70 (55 to 87) | 1.96 (1.59 to 2.38) | 129 (103 to 158) | 1.95 (1.57 to 2.36) | 0.02 (−0.02 to 0.06) |
| Niger | 90 (70 to 113) | 1.31 (1.02 to 1.62) | 273 (209 to 345) | 1.28 (1.01 to 1.58) | −0.10 (−0.14 to −0.05) |
| Nigeria | 1081 (807 to 1389) | 1.31 (0.99 to 1.67) | 2640 (1941 to 3421) | 1.23 (0.93 to 1.58) | −0.16 (−0.23 to −0.09) |
| North Korea | 521 (428 to 628) | 2.59 (2.15 to 3.11) | 632 (518 to 767) | 2.36 (1.93 to 2.86) | −0.31 (−0.37 to −0.26) |
| Northern Mariana Islands | 1 (1 to 1) | 2.25 (1.79 to 2.76) | 1 (1 to 1) | 2.14 (1.72 to 2.61) | −0.23 (−0.29 to −0.17) |
| Norway | 551 (471 to 644) | 9.77 (8.36 to 11.30) | 937 (800 to 1100) | 11.46 (9.82 to 13.29) | 0.59 (0.53 to 0.66) |
| Oman | 46 (37 to 58) | 2.42 (1.92 to 2.92) | 119 (94 to 149) | 2.50 (2.04 to 3.01) | 0.18 (0.14 to 0.22) |
| Pakistan | 1763 (1348 to 2242) | 1.66 (1.28 to 2.07) | 3885 (2947 to 4931) | 1.64 (1.27 to 2.04) | −0.06 (−0.12 to 0) |
| Palestine | 51 (40 to 62) | 2.60 (2.10 to 3.14) | 134 (107 to 165) | 2.58 (2.06 to 3.12) | −0.02 (−0.05 to 0.01) |
| Panama | 47 (37 to 58) | 2.06 (1.65 to 2.47) | 99 (82 to 118) | 2.29 (1.89 to 2.72) | 0.38 (0.37 to 0.40) |
| Papua New Guinea | 50 (39 to 63) | 1.27 (1.02 to 1.57) | 127 (99 to 160) | 1.25 (1.00 to 1.55) | −0.09 (−0.13 to −0.05) |
| Paraguay | 102 (83 to 123) | 2.68 (2.22 to 3.22) | 192 (153 to 231) | 2.68 (2.18 to 3.22) | 0 (−0.04 to 0.04) |
| Peru | 353 (277 to 438) | 1.74 (1.39 to 2.13) | 670 (536 to 817) | 1.84 (1.49 to 2.22) | 0.27 (0.23 to 0.30) |
| Philippines | 954 (726 to 1215) | 1.53 (1.20 to 1.92) | 1712 (1306 to 2180) | 1.49 (1.16 to 1.87) | −0.13 (−0.20 to −0.06) |
| Poland | 1403 (1150 to 1696) | 3.60 (2.94 to 4.32) | 2025 (1716 to 2364) | 4.39 (3.74 to 5.15) | 0.76 (0.72 to 0.80) |
| Portugal | 634 (539 to 738) | 5.39 (4.59 to 6.28) | 1264 (1084 to 1495) | 7.53 (6.47 to 8.79) | 1.16 (1.04 to 1.27) |
| Principality of Monaco | 3 (2 to 3) | 5.93 (4.98 to 7.03) | 4 (3 to 5) | 6.54 (5.55 to 7.62) | 0.34 (0.29 to 0.39) |
| Puerto Rico | 114 (93 to 135) | 3.12 (2.56 to 3.72) | 151 (130 to 175) | 3.80 (3.20 to 4.46) | 0.78 (0.73 to 0.83) |
| Qatar | 14 (11 to 17) | 2.94 (2.36 to 3.58) | 97 (76 to 123) | 2.95 (2.37 to 3.60) | 0.09 (0.05 to 0.13) |
| Republic of Nauru | 0 (0 to 0) | 1.36 (1.10 to 1.66) | 0 (0 to 0) | 1.25 (0.99 to 1.54) | −0.32 (−0.42 to −0.23) |
| Republic of Niue | 0 (0 to 0) | 2.01 (1.63 to 2.42) | 0 (0 to 0) | 1.95 (1.58 to 2.35) | −0.08 (−0.11 to −0.05) |
| Republic of Palau | 0 (0 to 0) | 1.72 (1.39 to 2.09) | 0 (0 to 0) | 1.61 (1.30 to 1.95) | −0.26 (−0.30 to −0.22) |
| Republic of San Marino | 2 (1 to 2) | 5.92 (4.92 to 7.12) | 3 (2 to 3) | 5.68 (4.76 to 6.77) | −0.15 (−0.25 to −0.04) |
| Romania | 721 (606 to 868) | 3.06 (2.57 to 3.68) | 645 (537 to 768) | 3.41 (2.81 to 4.11) | 0.45 (0.38 to 0.52) |
| Russian Federation | 5214 (4188 to 6329) | 3.49 (2.81 to 4.20) | 5491 (4575 to 6491) | 3.67 (2.99 to 4.34) | 0.34 (0.25 to 0.43) |
| Rwanda | 68 (52 to 88) | 1.08 (0.84 to 1.35) | 135 (102 to 173) | 1.08 (0.84 to 1.37) | 0.01 (−0.04 to 0.07) |
| Saint Kitts and Nevis | 1 (1 to 1) | 2.44 (1.99 to 2.93) | 2 (2 to 2) | 3.01 (2.52 to 3.53) | 0.75 (0.72 to 0.78) |
| Saint Lucia | 3 (3 to 4) | 2.37 (1.90 to 2.85) | 6 (5 to 6) | 2.93 (2.46 to 3.42) | 0.77 (0.74 to 0.79) |
| Saint Vincent and the Grenadines | 2 (2 to 3) | 2.16 (1.76 to 2.58) | 3 (2 to 3) | 2.47 (2.03 to 2.94) | 0.51 (0.48 to 0.54) |
| Samoa | 3 (2 to 4) | 1.74 (1.40 to 2.11) | 4 (3 to 4) | 1.69 (1.36 to 2.04) | −0.10 (−0.14 to −0.06) |
| Sao Tome and Principe | 1 (1 to 2) | 1.22 (0.96 to 1.50) | 3 (2 to 3) | 1.41 (1.14 to 1.69) | 0.61 (0.56 to 0.67) |
| Saudi Arabia | 387 (301 to 480) | 2.46 (1.97 to 2.98) | 952 (750 to 1184) | 2.41 (1.92 to 2.90) | −0.04 (−0.07 to −0.01) |
| Senegal | 94 (73 to 118) | 1.39 (1.08 to 1.73) | 203 (158 to 257) | 1.36 (1.08 to 1.69) | −0.07 (−0.11 to −0.03) |
| Serbia | 328 (269 to 394) | 3.39 (2.77 to 4.07) | 302 (250 to 363) | 3.34 (2.72 to 4.03) | 0 (−0.05 to 0.05) |
| Seychelles | 1 (1 to 2) | 1.86 (1.49 to 2.24) | 2 (2 to 3) | 2.03 (1.64 to 2.46) | 0.29 (0.26 to 0.32) |
| Sierra Leone | 43 (33 to 55) | 1.15 (0.90 to 1.43) | 91 (69 to 117) | 1.10 (0.86 to 1.36) | −0.14 (−0.18 to −0.10) |
| Singapore | 72 (59 to 86) | 2.47 (2.06 to 2.95) | 203 (169 to 239) | 2.90 (2.44 to 3.39) | 0.57 (0.53 to 0.62) |
| Slovakia | 200 (164 to 239) | 3.75 (3.07 to 4.48) | 207 (170 to 244) | 3.78 (3.09 to 4.50) | 0.07 (0.03 to 0.11) |
| Slovenia | 92 (77 to 108) | 4.58 (3.86 to 5.39) | 84 (69 to 101) | 3.97 (3.24 to 4.74) | −0.59 (−0.69 to −0.49) |
| Solomon Islands | 5 (3 to 6) | 1.34 (1.06 to 1.65) | 9 (7 to 11) | 1.29 (1.03 to 1.58) | −0.17 (−0.20 to −0.13) |
| Somalia | 70 (53 to 90) | 1.02 (0.80 to 1.28) | 180 (134 to 234) | 0.95 (0.74 to 1.19) | −0.24 (−0.29 to −0.20) |
| South Africa | 681 (521 to 857) | 1.87 (1.46 to 2.32) | 1028 (790 to 1301) | 1.78 (1.39 to 2.23) | −0.14 (−0.20 to −0.07) |
| South Korea | 1556 (1286 to 1846) | 3.65 (3.03 to 4.31) | 2395 (1960 to 2926) | 3.88 (3.20 to 4.61) | 0.12 (0.07 to 0.18) |
| South Sudan | 67 (52 to 85) | 1.31 (1.02 to 1.63) | 102 (77 to 131) | 1.20 (0.93 to 1.49) | −0.28 (−0.32 to −0.24) |
| Spain | 2991 (2575 to 3451) | 6.32 (5.45 to 7.30) | 5937 (5090 to 6962) | 8.23 (7.13 to 9.51) | 0.87 (0.76 to 0.97) |
| Sri Lanka | 279 (216 to 346) | 1.62 (1.27 to 1.97) | 384 (307 to 472) | 1.71 (1.36 to 2.13) | 0.17 (0.15 to 0.19) |
| Sudan | 301 (235 to 372) | 1.60 (1.26 to 1.95) | 682 (534 to 851) | 1.59 (1.26 to 1.97) | 0.04 (0 to 0.07) |
| Suriname | 7 (6 to 9) | 1.86 (1.49 to 2.27) | 12 (10 to 15) | 2.11 (1.75 to 2.50) | 0.48 (0.46 to 0.51) |
| Swaziland | 12 (10 to 16) | 1.67 (1.33 to 2.05) | 19 (15 to 24) | 1.65 (1.32 to 2.02) | −0.06 (−0.09 to −0.02) |
| Sweden | 1303 (1119 to 1518) | 10.65 (9.16 to 12.38) | 2051 (1736 to 2397) | 12.29 (10.57 to 14.16) | 0.57 (0.40 to 0.74) |
| Switzerland | 838 (721 to 969) | 9.39 (8.08 to 10.82) | 1396 (1193 to 1631) | 10.04 (8.54 to 11.61) | 0.22 (0.19 to 0.26) |
| Syria | 326 (259 to 400) | 2.66 (2.18 to 3.25) | 358 (289 to 439) | 2.56 (2.06 to 3.12) | −0.09 (−0.12 to −0.05) |
| Taiwan (Province of China) | 565 (457 to 678) | 2.75 (2.25 to 3.28) | 834 (703 to 974) | 3.17 (2.63 to 3.73) | 0.16 (0 to 0.32) |
| Tajikistan | 117 (93 to 144) | 2.28 (1.81 to 2.78) | 216 (172 to 266) | 2.14 (1.73 to 2.63) | −0.15 (−0.24 to −0.06) |
| Tanzania | 283 (221 to 357) | 1.27 (0.99 to 1.57) | 667 (512 to 850) | 1.26 (0.98 to 1.57) | −0.02 (−0.07 to 0.03) |
| Thailand | 1015 (799 to 1266) | 1.78 (1.43 to 2.19) | 1255 (1009 to 1548) | 1.84 (1.47 to 2.27) | 0.06 (0.02 to 0.09) |
| The Bahamas | 8 (6 to 9) | 3.01 (2.44 to 3.57) | 13 (11 to 15) | 3.22 (2.69 to 3.77) | 0.28 (0.25 to 0.32) |
| The Gambia | 12 (9 to 15) | 1.37 (1.08 to 1.70) | 29 (22 to 38) | 1.30 (1.02 to 1.60) | −0.16 (−0.20 to −0.12) |
| Timor-Leste | 10 (8 to 12) | 1.34 (1.06 to 1.66) | 18 (14 to 23) | 1.36 (1.08 to 1.67) | 0.09 (0.07 to 0.11) |
| Togo | 38 (29 to 49) | 1.18 (0.92 to 1.47) | 90 (70 to 116) | 1.14 (0.89 to 1.42) | −0.13 (−0.17 to −0.09) |
| Tokelau | 0 (0 to 0) | 1.62 (1.31 to 1.97) | 0 (0 to 0) | 1.59 (1.27 to 1.95) | −0.04 (−0.08 to −0.01) |
| Tonga | 2 (2 to 2) | 1.96 (1.58 to 2.38) | 2 (2 to 2) | 1.92 (1.54 to 2.33) | −0.10 (−0.14 to −0.06) |
| Trinidad and Tobago | 26 (21 to 32) | 2.20 (1.76 to 2.65) | 37 (31 to 44) | 2.52 (2.09 to 2.98) | 0.53 (0.48 to 0.57) |
| Tunisia | 230 (183 to 278) | 2.79 (2.28 to 3.37) | 333 (271 to 402) | 2.78 (2.24 to 3.35) | −0.01 (−0.05 to 0.03) |
| Turkey | 2002 (1672 to 2364) | 3.64 (3.06 to 4.28) | 3548 (3010 to 4137) | 4.06 (3.47 to 4.70) | 0.42 (0.38 to 0.46) |
| Turkmenistan | 84 (66 to 104) | 2.31 (1.87 to 2.80) | 123 (98 to 149) | 2.38 (1.92 to 2.88) | 0.18 (0.11 to 0.26) |
| Tuvalu | 0 (0 to 0) | 1.33 (1.07 to 1.63) | 0 (0 to 0) | 1.30 (1.05 to 1.60) | −0.14 (−0.18 to −0.09) |
| Uganda | 162 (121 to 206) | 1.10 (0.85 to 1.38) | 419 (314 to 545) | 1.10 (0.85 to 1.38) | −0.02 (−0.05 to 0.02) |
| Ukraine | 1652 (1325 to 2005) | 3.20 (2.56 to 3.84) | 1254 (1020 to 1488) | 3.01 (2.43 to 3.60) | −0.12 (−0.20 to −0.04) |
| United Arab Emirates | 56 (44 to 70) | 2.89 (2.32 to 3.50) | 281 (217 to 358) | 2.77 (2.23 to 3.32) | −0.13 (−0.17 to −0.09) |
| United Kingdom | 6578 (5684 to 7604) | 8.70 (7.48 to 10.00) | 11919 (10168 to 13932) | 11.16 (9.67 to 12.84) | 0.91 (0.83 to 0.99) |
| United States | 22197 (19257 to 25430) | 7.82 (6.82 to 8.92) | 40495 (37645 to 43576) | 8.82 (8.26 to 9.51) | 1.16 (0.87 to 1.44) |
| Uruguay | 126 (106 to 147) | 3.81 (3.19 to 4.46) | 227 (195 to 261) | 5.44 (4.64 to 6.25) | 1.17 (1.10 to 1.24) |
| Uzbekistan | 493 (391 to 608) | 2.39 (1.92 to 2.90) | 813 (651 to 1000) | 2.38 (1.92 to 2.92) | 0.03 (−0.03 to 0.09) |
| Vanuatu | 2 (2 to 3) | 1.58 (1.26 to 1.91) | 5 (4 to 6) | 1.51 (1.21 to 1.85) | −0.15 (−0.18 to −0.11) |
| Venezuela | 358 (279 to 440) | 1.98 (1.59 to 2.38) | 566 (468 to 669) | 2.07 (1.70 to 2.45) | 0.27 (0.24 to 0.30) |
| Vietnam | 1157 (903 to 1433) | 1.71 (1.38 to 2.08) | 1823 (1457 to 2242) | 1.81 (1.45 to 2.20) | 0.19 (0.16 to 0.23) |
| Virgin Islands, U.S. | 3 (3 to 4) | 3.16 (2.64 to 3.72) | 5 (4 to 5) | 4.00 (3.41 to 4.59) | 0.83 (0.72 to 0.94) |
| Yemen | 204 (160 to 257) | 1.62 (1.28 to 1.99) | 512 (401 to 636) | 1.54 (1.23 to 1.89) | −0.11 (−0.15 to −0.07) |
| Zambia | 96 (74 to 122) | 1.40 (1.10 to 1.74) | 245 (187 to 313) | 1.37 (1.08 to 1.70) | −0.03 (−0.08 to 0.02) |
| Zimbabwe | 143 (110 to 182) | 1.48 (1.17 to 1.82) | 212 (165 to 269) | 1.41 (1.13 to 1.74) | −0.21 (−0.26 to −0.16) |

Abbreviations: ASR, age-standardized rate; MND, motor neuron disease; EAPC, estimated annual percentage change; SDI, sociodemographic index; UI, uncertainty interval; CI, confidence interval.

**Table S6.** The case number and ASR of deaths of MND in 1990 and 2021, with EAPC from 1990 to 2021.

|  | **1990** | | **2021** | | **EAPC (95% CI)** |
| --- | --- | --- | --- | --- | --- |
|  | **Number (95% UI)** | **ASR (95% UI)** | **Number (95% UI)** | **ASR (95% UI)** |  |
| **Sex** |  |  |  |  |  |
| Female | 7248 (6819 to 7577) | 0.33 (0.31 to 0.35) | 17379 (15419 to 19638) | 0.38 (0.34 to 0.43) | 0.50 (0.39 to 0.61) |
| Male | 8012 (7137 to 8579) | 0.44 (0.40 to 0.46) | 21703 (19623 to 23371) | 0.55 (0.50 to 0.59) | 0.84 (0.71 to 0.97) |
| **Age** |  |  |  |  |  |
| <5 years | 1060 (851 to 1278) | 0.17 (0.14 to 0.21) | 441 (349 to 553) | 0.07 (0.05 to 0.08) | −3.05 (−3.21 to −2.88) |
| 5-9 years | 55 (42 to 64) | 0.01 (0.01 to 0.01) | 49 (40 to 58) | 0.01 (0.01 to 0.01) | −0.99 (−1.18 to −0.80) |
| 10-14 years | 56 (41 to 67) | 0.01 (0.01 to 0.01) | 62 (51 to 75) | 0.01 (0.01 to 0.01) | −0.75 (−0.99 to −0.51) |
| 15-19 years | 123 (70 to 159) | 0.02 (0.01 to 0.03) | 112 (80 to 139) | 0.02 (0.01 to 0.02) | −1.29 (−1.49 to −1.10) |
| 20-24 years | 93 (56 to 120) | 0.02 (0.01 to 0.02) | 112 (84 to 142) | 0.02 (0.01 to 0.02) | −0.37 (−0.58 to −0.15) |
| 25-29 years | 74 (59 to 86) | 0.02 (0.01 to 0.02) | 116 (96 to 129) | 0.02 (0.02 to 0.02) | 0.37 (0.20 to 0.55) |
| 30-34 years | 117 (100 to 128) | 0.03 (0.03 to 0.03) | 211 (176 to 241) | 0.03 (0.03 to 0.04) | 0.20 (0.07 to 0.34) |
| 35-39 years | 205 (183 to 224) | 0.06 (0.05 to 0.06) | 382 (339 to 419) | 0.07 (0.06 to 0.07) | 0.07 (−0.10 to 0.25) |
| 40-44 years | 354 (321 to 381) | 0.12 (0.11 to 0.13) | 696 (615 to 760) | 0.14 (0.12 to 0.15) | 0.05 (−0.14 to 0.25) |
| 45-49 years | 534 (493 to 574) | 0.23 (0.21 to 0.25) | 1246 (1116 to 1365) | 0.26 (0.24 to 0.29) | 0.28 (0.09 to 0.47) |
| 50-54 years | 898 (831 to 969) | 0.42 (0.39 to 0.46) | 2243 (2033 to 2450) | 0.50 (0.46 to 0.55) | 0.52 (0.32 to 0.71) |
| 55-59 years | 1379 (1283 to 1465) | 0.74 (0.69 to 0.79) | 3557 (3288 to 3851) | 0.90 (0.83 to 0.97) | 0.76 (0.61 to 0.91) |
| 60-64 years | 1962 (1863 to 2061) | 1.22 (1.16 to 1.28) | 4932 (4596 to 5314) | 1.54 (1.44 to 1.66) | 0.92 (0.81 to 1.02) |
| 65-69 years | 2488 (2386 to 2590) | 2.01 (1.93 to 2.10) | 6226 (5775 to 6721) | 2.26 (2.09 to 2.44) | 0.72 (0.60 to 0.83) |
| 70-74 years | 2111 (2013 to 2187) | 2.49 (2.38 to 2.58) | 6785 (6214 to 7389) | 3.30 (3.02 to 3.59) | 0.79 (0.71 to 0.86) |
| 75-79 years | 1980 (1860 to 2053) | 3.22 (3.02 to 3.34) | 5520 (4933 to 6070) | 4.19 (3.74 to 4.60) | 0.88 (0.68 to 1.09) |
| 80-84 years | 1098 (982 to 1162) | 3.10 (2.78 to 3.28) | 3692 (3065 to 4133) | 4.22 (3.50 to 4.72) | 1.13 (0.81 to 1.46) |
| 85-89 years | 534 (458 to 584) | 3.53 (3.03 to 3.87) | 1854 (1489 to 2106) | 4.06 (3.26 to 4.61) | 1.10 (0.78 to 1.42) |
| 90-94 years | 117 (96 to 129) | 2.73 (2.23 to 3.00) | 651 (501 to 745) | 3.64 (2.80 to 4.17) | 1.46 (1.16 to 1.76) |
| 95+ years | 22 (17 to 25) | 2.19 (1.66 to 2.45) | 193 (140 to 226) | 3.55 (2.57 to 4.14) | 1.82 (1.59 to 2.05) |
| **Country** |  |  |  |  |  |
| Afghanistan | 0 (0 to 0) | 0 (0 to 0) | 0 (0 to 0) | 0 (0 to 0) | 8.49 (7.47 to 9.53) |
| Albania | 2 (1 to 3) | 0.07 (0.04 to 0.10) | 3 (2 to 5) | 0.09 (0.05 to 0.14) | 1.14 (0.97 to 1.31) |
| Algeria | 0 (0 to 0) | 0 (0 to 0) | 1 (0 to 2) | 0 (0 to 0.01) | 6.34 (5.92 to 6.77) |
| American Samoa | 0 (0 to 0) | 0.01 (0 to 0.02) | 0 (0 to 0) | 0 (0 to 0.01) | −6.72 (−7.73 to −5.69) |
| Andorra | 1 (0 to 1) | 1.26 (0.63 to 2.00) | 2 (1 to 3) | 1.43 (0.85 to 2.11) | 0.97 (0.74 to 1.19) |
| Angola | 0 (0 to 0) | 0 (0 to 0) | 0 (0 to 0) | 0 (0 to 0) | −3.22 (−4.05 to −2.39) |
| Antigua and Barbuda | 0 (0 to 0) | 0.01 (0.01 to 0.01) | 0 (0 to 1) | 0.46 (0.41 to 0.51) | 10.97 (7.56 to 14.49) |
| Argentina | 16 (15 to 17) | 0.05 (0.05 to 0.05) | 351 (318 to 391) | 0.64 (0.58 to 0.71) | 7.44 (5.45 to 9.47) |
| Armenia | 2 (1 to 3) | 0.05 (0.04 to 0.09) | 5 (4 to 6) | 0.12 (0.09 to 0.15) | 2.61 (1.88 to 3.35) |
| Australia | 308 (290 to 322) | 1.59 (1.50 to 1.66) | 822 (721 to 915) | 1.87 (1.66 to 2.07) | 0.70 (0.41 to 0.99) |
| Austria | 91 (85 to 96) | 0.82 (0.78 to 0.86) | 194 (177 to 211) | 1.14 (1.05 to 1.22) | 1.49 (1.30 to 1.67) |
| Azerbaijan | 0 (0 to 0) | 0 (0 to 0) | 1 (0 to 2) | 0.01 (0 to 0.02) | 6.16 (5.51 to 6.81) |
| Bahrain | 0 (0 to 0) | 0 (0 to 0.01) | 0 (0 to 0) | 0.01 (0 to 0.01) | 1.02 (0.56 to 1.48) |
| Bangladesh | 1 (1 to 3) | 0 (0 to 0.01) | 21 (6 to 40) | 0.01 (0 to 0.03) | 6.23 (6.09 to 6.37) |
| Barbados | 0 (0 to 0) | 0.02 (0.02 to 0.02) | 6 (4 to 8) | 1.15 (0.80 to 1.66) | 12.39 (8.86 to 16.04) |
| Belarus | 17 (10 to 37) | 0.14 (0.09 to 0.30) | 102 (82 to 124) | 0.66 (0.54 to 0.81) | 4.82 (4.28 to 5.37) |
| Belgium | 394 (363 to 425) | 2.54 (2.34 to 2.72) | 395 (345 to 450) | 1.83 (1.61 to 2.07) | 0.78 (−0.02 to 1.58) |
| Belize | 0 (0 to 0) | 0.01 (0.01 to 0.01) | 1 (1 to 1) | 0.27 (0.22 to 0.31) | 11.49 (7.79 to 15.31) |
| Benin | 0 (0 to 0) | 0 (0 to 0) | 0 (0 to 0) | 0 (0 to 0) | 0.38 (0.08 to 0.69) |
| Bermuda | 0 (0 to 0) | 0.03 (0.02 to 0.05) | 1 (0 to 1) | 0.44 (0.34 to 0.58) | 7.89 (5.44 to 10.40) |
| Bhutan | 0 (0 to 0) | 0 (0 to 0) | 0 (0 to 0) | 0.01 (0 to 0.02) | 7.67 (7.45 to 7.89) |
| Bolivia | 0 (0 to 0) | 0.01 (0 to 0.01) | 13 (7 to 19) | 0.13 (0.08 to 0.20) | 11.16 (10.01 to 12.31) |
| Bosnia and Herzegovina | 7 (4 to 10) | 0.17 (0.09 to 0.22) | 17 (10 to 23) | 0.30 (0.18 to 0.42) | 2.36 (2.18 to 2.54) |
| Botswana | 0 (0 to 0) | 0.01 (0 to 0.02) | 0 (0 to 0) | 0.01 (0 to 0.01) | −2.87 (−3.16 to −2.58) |
| Brazil | 313 (303 to 324) | 0.31 (0.30 to 0.32) | 1873 (1745 to 2006) | 0.74 (0.69 to 0.80) | 3.17 (3.06 to 3.28) |
| Brunei | 0 (0 to 0) | 0.22 (0.07 to 0.41) | 1 (1 to 2) | 0.37 (0.17 to 0.51) | 2.05 (1.92 to 2.18) |
| Bulgaria | 8 (7 to 9) | 0.08 (0.07 to 0.09) | 20 (17 to 25) | 0.17 (0.14 to 0.21) | 2.95 (2.68 to 3.21) |
| Burkina Faso | 0 (0 to 0) | 0 (0 to 0) | 0 (0 to 0) | 0 (0 to 0) | 0.34 (0.08 to 0.6) |
| Burundi | 0 (0 to 0) | 0 (0 to 0) | 0 (0 to 0) | 0 (0 to 0) | −5.8 (−6.76 to −4.83) |
| Cambodia | 0 (0 to 0) | 0 (0 to 0.01) | 1 (0 to 2) | 0.01 (0 to 0.01) | 2.37 (2.22 to 2.52) |
| Cameroon | 0 (0 to 0) | 0 (0 to 0) | 1 (0 to 1) | 0 (0 to 0) | 1.01 (0.75 to 1.28) |
| Canada | 437 (415 to 455) | 1.37 (1.30 to 1.42) | 1105 (1010 to 1188) | 1.57 (1.45 to 1.68) | 0.44 (0.18 to 0.69) |
| Cape Verde | 0 (0 to 0) | 0 (0 to 0) | 0 (0 to 0) | 0 (0 to 0) | 2.10 (2 to 2.2) |
| Central African Republic | 0 (0 to 0) | 0 (0 to 0) | 0 (0 to 0) | 0 (0 to 0) | −4.88 (−5.59 to −4.17) |
| Chad | 0 (0 to 0) | 0 (0 to 0) | 0 (0 to 0) | 0 (0 to 0) | 1.55 (1.31 to 1.8) |
| Chile | 16 (15 to 17) | 0.15 (0.14 to 0.16) | 220 (198 to 245) | 0.88 (0.79 to 0.97) | 3.97 (2.97 to 4.98) |
| China | 1528 (845 to 1953) | 0.15 (0.09 to 0.19) | 3450 (2220 to 4791) | 0.18 (0.11 to 0.25) | −0.6 (−1.14 to −0.04) |
| Colombia | 49 (46 to 51) | 0.24 (0.23 to 0.25) | 303 (248 to 364) | 0.56 (0.45 to 0.67) | 3.09 (2.84 to 3.35) |
| Comoros | 0 (0 to 0) | 0 (0 to 0) | 0 (0 to 0) | 0 (0 to 0) | −4.06 (−4.81 to −3.3) |
| Congo | 0 (0 to 0) | 0 (0 to 0) | 0 (0 to 0) | 0 (0 to 0) | −3.69 (−4.51 to −2.86) |
| Cook Islands | 0 (0 to 0) | 0.01 (0 to 0.01) | 0 (0 to 0) | 0.01 (0 to 0.01) | −1.39 (−1.62 to −1.16) |
| Costa Rica | 6 (6 to 6) | 0.32 (0.30 to 0.34) | 56 (49 to 65) | 1.03 (0.89 to 1.19) | 3.86 (3.46 to 4.27) |
| Cote d'Ivoire | 0 (0 to 0) | 0 (0 to 0) | 0 (0 to 1) | 0 (0 to 0) | 0.41 (0.2 to 0.62) |
| Croatia | 26 (24 to 28) | 0.44 (0.41 to 0.48) | 59 (50 to 73) | 0.76 (0.64 to 0.94) | 2.48 (2.2 to 2.76) |
| Cuba | 1 (1 to 1) | 0.01 (0.01 to 0.01) | 124 (103 to 147) | 0.67 (0.56 to 0.79) | 11.91 (8.27 to 15.68) |
| Cyprus | 6 (2 to 10) | 0.74 (0.3 to 1.28) | 18 (11 to 25) | 0.87 (0.55 to 1.19) | 0.4 (0.16 to 0.64) |
| Czech Republic | 51 (47 to 57) | 0.41 (0.37 to 0.45) | 120 (102 to 140) | 0.63 (0.54 to 0.74) | 2.21 (1.82 to 2.6) |
| Democratic Republic of the Congo | 0 (0 to 1) | 0 (0 to 0) | 0 (0 to 1) | 0 (0 to 0) | −4.36 (−5.81 to −2.89) |
| Denmark | 97 (88 to 106) | 1.29 (1.18 to 1.4) | 192 (173 to 215) | 1.63 (1.47 to 1.82) | 0.71 (0.54 to 0.88) |
| Djibouti | 0 (0 to 0) | 0 (0 to 0) | 0 (0 to 0) | 0 (0 to 0) | −4.46 (−5.08 to −3.84) |
| Dominica | 0 (0 to 0) | 0.01 (0.01 to 0.03) | 0 (0 to 1) | 0.63 (0.44 to 0.83) | 11.87 (9.28 to 14.52) |
| Dominican Republic | 0 (0 to 0) | 0 (0 to 0.01) | 0 (0 to 1) | 0 (0 to 0.01) | 1.13 (−0.38 to 2.67) |
| Ecuador | 0 (0 to 0) | 0 (0 to 0) | 82 (63 to 104) | 0.5 (0.38 to 0.62) | 25.61 (18.24 to 33.43) |
| Egypt | 4 (3 to 6) | 0.01 (0 to 0.01) | 0 (0 to 0) | 0 (0 to 0) | −17.47 (−20.14 to −14.72) |
| El Salvador | 1 (1 to 2) | 0.04 (0.02 to 0.05) | 7 (5 to 9) | 0.11 (0.07 to 0.15) | 3.59 (3.45 to 3.73) |
| Equatorial Guinea | 0 (0 to 0) | 0 (0 to 0) | 0 (0 to 0) | 0 (0 to 0) | −0.04 (−0.46 to 0.38) |
| Eritrea | 0 (0 to 0) | 0 (0 to 0) | 0 (0 to 0) | 0 (0 to 0) | −3.02 (−3.56 to −2.49) |
| Estonia | 7 (6 to 9) | 0.35 (0.29 to 0.44) | 19 (17 to 22) | 0.81 (0.70 to 0.94) | 4.06 (1.27 to 6.93) |
| Ethiopia | 0 (0 to 0) | 0 (0 to 0) | 0 (0 to 0) | 0 (0 to 0) | −3.27 (−4.25 to −2.27) |
| Federated States of Micronesia | 0 (0 to 0) | 0 (0 to 0.01) | 0 (0 to 0) | 0 (0 to 0) | −1.69 (−1.94 to −1.44) |
| Fiji | 0 (0 to 0) | 0 (0 to 0) | 0 (0 to 0) | 0 (0 to 0.01) | 4.65 (3.75 to 5.56) |
| Finland | 122 (115 to 129) | 1.75 (1.65 to 1.84) | 257 (208 to 316) | 2.14 (1.77 to 2.59) | 0.84 (0.73 to 0.95) |
| France | 1107 (1043 to 1160) | 1.43 (1.36 to 1.49) | 2138 (1815 to 2511) | 1.65 (1.41 to 1.93) | 0.68 (0.60 to 0.76) |
| Gabon | 0 (0 to 0) | 0 (0 to 0.01) | 0 (0 to 0) | 0 (0 to 0) | −3.75 (−4.43 to −3.07) |
| Georgia | 0 (0 to 0) | 0 (0 to 0) | 10 (8 to 12) | 0.20 (0.16 to 0.23) | 19.83 (16.69 to 23.06) |
| Germany | 1270 (1208 to 1332) | 1.08 (1.03 to 1.13) | 2395 (2173 to 2582) | 1.33 (1.23 to 1.43) | 1.08 (0.85 to 1.31) |
| Ghana | 0 (0 to 0) | 0 (0 to 0) | 1 (1 to 2) | 0 (0 to 0.01) | 1.68 (1.43 to 1.93) |
| Greece | 58 (55 to 62) | 0.40 (0.38 to 0.43) | 219 (195 to 247) | 1.02 (0.90 to 1.14) | 3.11 (3.00 to 3.23) |
| Greenland | 0 (0 to 0) | 0.31 (0.08 to 0.40) | 0 (0 to 0) | 0.31 (0.09 to 0.43) | 0.56 (0.41 to 0.71) |
| Grenada | 0 (0 to 0) | 0.01 (0.01 to 0.01) | 1 (0 to 1) | 0.45 (0.38 to 0.52) | 11.40 (8.16 to 14.73) |
| Guam | 0 (0 to 0) | 0.10 (0.05 to 0.13) | 0 (0 to 0) | 0 (0 to 0.01) | −13.28 (−14.52 to −12.03) |
| Guatemala | 3 (2 to 3) | 0.05 (0.05 to 0.05) | 14 (12 to 17) | 0.12 (0.10 to 0.14) | 2.98 (2.72 to 3.23) |
| Guinea | 0 (0 to 0) | 0 (0 to 0) | 0 (0 to 0) | 0 (0 to 0) | 1.32 (1.14 to 1.50) |
| Guinea-Bissau | 0 (0 to 0) | 0 (0 to 0) | 0 (0 to 0) | 0 (0 to 0) | 0.71 (0.55 to 0.87) |
| Guyana | 0 (0 to 0) | 0.01 (0.01 to 0.01) | 3 (2 to 4) | 0.49 (0.36 to 0.63) | 11.81 (8.50 to 15.22) |
| Haiti | 0 (0 to 1) | 0.01 (0 to 0.02) | 8 (3 to 15) | 0.09 (0.03 to 0.17) | 7.77 (6.92 to 8.62) |
| Honduras | 2 (1 to 3) | 0.07 (0.05 to 0.09) | 18 (11 to 26) | 0.26 (0.15 to 0.39) | 4.39 (4.26 to 4.52) |
| Hungary | 59 (53 to 64) | 0.46 (0.42 to 0.50) | 140 (120 to 162) | 0.83 (0.72 to 0.97) | 2.57 (2.26 to 2.88) |
| Iceland | 3 (3 to 3) | 1.07 (1 to 1.14) | 7 (6 to 8) | 1.25 (1.10 to 1.41) | 0.82 (0.61 to 1.03) |
| India | 28 (14 to 60) | 0 (0 to 0.01) | 292 (147 to 440) | 0.02 (0.01 to 0.04) | 5.10 (4.89 to 5.31) |
| Indonesia | 9 (4 to 14) | 0.01 (0 to 0.01) | 35 (20 to 62) | 0.01 (0.01 to 0.02) | 1.66 (1.43 to 1.89) |
| Iran | 4 (3 to 6) | 0.01 (0.01 to 0.02) | 34 (25 to 46) | 0.04 (0.03 to 0.06) | 4.30 (4.10 to 4.50) |
| Iraq | 0 (0 to 0) | 0 (0 to 0) | 0 (0 to 0) | 0 (0 to 0) | 4.54 (4.41 to 4.68) |
| Ireland | 68 (64 to 72) | 1.67 (1.56 to 1.76) | 161 (133 to 191) | 2.07 (1.70 to 2.45) | 1.01 (0.88 to 1.14) |
| Israel | 38 (36 to 40) | 0.78 (0.74 to 0.81) | 110 (96 to 121) | 0.91 (0.80 to 1.00) | 0.60 (0.46 to 0.73) |
| Italy | 693 (663 to 714) | 0.88 (0.84 to 0.90) | 1749 (1530 to 1970) | 1.31 (1.17 to 1.47) | 1.40 (1.12 to 1.68) |
| Jamaica | 0 (0 to 0) | 0.01 (0.01 to 0.01) | 15 (11 to 20) | 0.50 (0.35 to 0.66) | 12.47 (8.66 to 16.41) |
| Japan | 1008 (964 to 1038) | 0.59 (0.57 to 0.61) | 3008 (2593 to 3327) | 0.86 (0.77 to 0.94) | 1.25 (1.11 to 1.40) |
| Jordan | 0 (0 to 1) | 0.02 (0.01 to 0.03) | 4 (3 to 6) | 0.05 (0.03 to 0.07) | 4.33 (3.81 to 4.85) |
| Kazakhstan | 0 (0 to 0) | 0 (0 to 0) | 0 (0 to 0) | 0 (0 to 0) | 4.18 (0.88 to 7.60) |
| Kenya | 0 (0 to 0) | 0 (0 to 0) | 0 (0 to 0) | 0 (0 to 0) | −2.35 (−3.07 to −1.61) |
| Kiribati | 0 (0 to 0) | 0.10 (0.07 to 0.14) | 0 (0 to 0) | 0.18 (0.12 to 0.27) | 2.08 (1.89 to 2.26) |
| Kuwait | 4 (3 to 4) | 0.34 (0.29 to 0.38) | 8 (6 to 10) | 0.23 (0.17 to 0.29) | 0.29 (−1.71 to 2.33) |
| Kyrgyzstan | 0 (0 to 0) | 0 (0 to 0) | 7 (6 to 9) | 0.12 (0.10 to 0.15) | 19.48 (16.26 to 22.80) |
| Laos | 0 (0 to 0) | 0 (0 to 0.01) | 0 (0 to 1) | 0.01 (0 to 0.01) | 3.54 (3.39 to 3.68) |
| Latvia | 2 (2 to 3) | 0.07 (0.06 to 0.10) | 28 (23 to 32) | 0.81 (0.69 to 0.93) | 9.31 (7.40 to 11.27) |
| Lebanon | 1 (0 to 2) | 0.04 (0.02 to 0.09) | 6 (4 to 9) | 0.12 (0.07 to 0.15) | 4.22 (3.93 to 4.51) |
| Lesotho | 0 (0 to 0) | 0 (0 to 0.01) | 0 (0 to 0) | 0 (0 to 0.01) | −1.39 (−1.64 to −1.15) |
| Liberia | 0 (0 to 0) | 0 (0 to 0) | 0 (0 to 0) | 0 (0 to 0) | 0.50 (−0.05 to 1.06) |
| Libya | 0 (0 to 0) | 0 (0 to 0) | 0 (0 to 0) | 0 (0 to 0.01) | 9.02 (8.53 to 9.51) |
| Lithuania | 18 (15 to 22) | 0.41 (0.34 to 0.50) | 72 (62 to 83) | 1.46 (1.24 to 1.65) | 4.38 (2.96 to 5.81) |
| Luxembourg | 6 (5 to 6) | 1.08 (1.02 to 1.14) | 11 (10 to 13) | 1.10 (0.96 to 1.26) | 0.48 (0.29 to 0.67) |
| Macedonia | 3 (2 to 4) | 0.16 (0.10 to 0.21) | 6 (4 to 9) | 0.20 (0.12 to 0.28) | 1.18 (0.99 to 1.37) |
| Madagascar | 0 (0 to 0) | 0 (0 to 0) | 0 (0 to 0) | 0 (0 to 0) | −5.00 (−5.94 to −4.04) |
| Malawi | 0 (0 to 0) | 0 (0 to 0) | 0 (0 to 0) | 0 (0 to 0) | −3.49 (−4.31 to −2.66) |
| Malaysia | 2 (1 to 3) | 0.01 (0.01 to 0.02) | 6 (3 to 11) | 0.02 (0.01 to 0.04) | 0.75 (0.53 to 0.98) |
| Maldives | 0 (0 to 0) | 0.04 (0.02 to 0.09) | 1 (0 to 1) | 0.20 (0.11 to 0.29) | 4.94 (4.50 to 5.39) |
| Mali | 0 (0 to 0) | 0 (0 to 0) | 0 (0 to 0) | 0 (0 to 0) | 0.97 (0.65 to 1.28) |
| Malta | 4 (4 to 4) | 0.96 (0.89 to 1.02) | 11 (9 to 13) | 1.21 (1.04 to 1.41) | 0.81 (0.63 to 1.00) |
| Marshall Islands | 0 (0 to 0) | 0 (0 to 0.01) | 0 (0 to 0) | 0 (0 to 0.01) | −0.48 (−0.81 to −0.15) |
| Mauritania | 0 (0 to 0) | 0 (0 to 0) | 0 (0 to 0) | 0 (0 to 0) | 0.36 (0.05 to 0.68) |
| Mauritius | 0 (0 to 1) | 0.06 (0.06 to 0.06) | 12 (11 to 13) | 0.65 (0.58 to 0.72) | 8.75 (6.02 to 11.54) |
| Mexico | 109 (106 to 111) | 0.21 (0.20 to 0.21) | 575 (500 to 661) | 0.44 (0.38 to 0.50) | 2.32 (2.14 to 2.49) |
| Moldova | 14 (13 to 15) | 0.30 (0.28 to 0.33) | 22 (19 to 26) | 0.39 (0.34 to 0.44) | 3.78 (0.42 to 7.25) |
| Mongolia | 1 (0 to 3) | 0.07 (0.02 to 0.14) | 2 (2 to 4) | 0.08 (0.05 to 0.12) | −0.24 (−0.54 to 0.05) |
| Montenegro | 0 (0 to 0) | 0.01 (0.01 to 0.01) | 0 (0 to 0) | 0.01 (0.01 to 0.02) | 1.93 (1.80 to 2.07) |
| Morocco | 0 (0 to 0) | 0 (0 to 0) | 0 (0 to 2) | 0 (0 to 0) | 7.94 (7.37 to 8.51) |
| Mozambique | 0 (0 to 0) | 0 (0 to 0) | 0 (0 to 0) | 0 (0 to 0) | −2.89 (−3.76 to −2.01) |
| Myanmar | 1 (0 to 2) | 0 (0 to 0.01) | 4 (2 to 8) | 0.01 (0 to 0.02) | 3.06 (3.03 to 3.11) |
| Namibia | 0 (0 to 0) | 0.01 (0 to 0.02) | 0 (0 to 0) | 0.01 (0 to 0.01) | −2.23 (−2.61 to −1.86) |
| Nepal | 0 (0 to 0) | 0 (0 to 0) | 2 (1 to 5) | 0.01 (0 to 0.02) | 7.60 (7.24 to 7.95) |
| Netherlands | 290 (273 to 303) | 1.51 (1.44 to 1.58) | 634 (568 to 698) | 1.85 (1.68 to 2.04) | 0.80 (0.63 to 0.97) |
| New Zealand | 67 (64 to 71) | 1.71 (1.61 to 1.80) | 138 (122 to 154) | 1.66 (1.48 to 1.85) | 0.11 (−0.26 to 0.48) |
| Nicaragua | 1 (0 to 1) | 0.04 (0.02 to 0.05) | 5 (3 to 7) | 0.10 (0.06 to 0.14) | 3.59 (3.28 to 3.90) |
| Niger | 0 (0 to 0) | 0 (0 to 0) | 0 (0 to 0) | 0 (0 to 0) | −0.78 (−1.14 to −0.42) |
| Nigeria | 1 (1 to 1) | 0 (0 to 0) | 4 (3 to 7) | 0 (0 to 0) | 2.72 (2.29 to 3.15) |
| North Korea | 15 (7 to 26) | 0.08 (0.04 to 0.13) | 21 (11 to 44) | 0.07 (0.03 to 0.14) | −0.35 (−0.59 to −0.11) |
| Northern Mariana Islands | 0 (0 to 0) | 0 (0 to 0.01) | 0 (0 to 0) | 0 (0 to 0.01) | −2.16 (−3.55 to −0.76) |
| Norway | 94 (90 to 97) | 1.45 (1.39 to 1.49) | 164 (150 to 178) | 1.69 (1.56 to 1.82) | 0.45 (0.27 to 0.64) |
| Oman | 0 (0 to 0) | 0 (0 to 0.01) | 1 (1 to 1) | 0.04 (0.03 to 0.06) | 9.93 (9.37 to 10.49) |
| Pakistan | 3 (1 to 5) | 0 (0 to 0.01) | 33 (15 to 53) | 0.02 (0.01 to 0.04) | 5.88 (5.55 to 6.20) |
| Palestine | 0 (0 to 1) | 0.02 (0.01 to 0.04) | 2 (2 to 3) | 0.06 (0.05 to 0.08) | 3.92 (3.61 to 4.23) |
| Panama | 2 (2 to 2) | 0.14 (0.13 to 0.14) | 19 (14 to 23) | 0.43 (0.33 to 0.52) | 3.82 (3.60 to 4.03) |
| Papua New Guinea | 0 (0 to 0) | 0 (0 to 0) | 0 (0 to 0) | 0 (0 to 0) | −2.58 (−3.15 to −2.01) |
| Paraguay | 0 (0 to 1) | 0.02 (0.01 to 0.02) | 3 (2 to 4) | 0.05 (0.03 to 0.07) | 4.15 (4.07 to 4.23) |
| Peru | 1 (0 to 1) | 0.01 (0 to 0.01) | 64 (37 to 91) | 0.19 (0.11 to 0.27) | 11.77 (10.10 to 13.47) |
| Philippines | 7 (3 to 10) | 0.02 (0.01 to 0.03) | 19 (13 to 24) | 0.02 (0.02 to 0.03) | 0.20 (−0.39 to 0.79) |
| Poland | 87 (85 to 90) | 0.22 (0.21 to 0.22) | 616 (557 to 683) | 0.95 (0.86 to 1.04) | 4.78 (4.05 to 5.51) |
| Portugal | 90 (85 to 95) | 0.70 (0.67 to 0.74) | 253 (225 to 286) | 1.11 (0.99 to 1.25) | 1.63 (1.49 to 1.76) |
| Principality of Monaco | 0 (0 to 0) | 0.21 (0.14 to 0.28) | 0 (0 to 0) | 0.39 (0.28 to 0.54) | 2.19 (1.82 to 2.56) |
| Puerto Rico | 1 (1 to 1) | 0.02 (0.02 to 0.02) | 37 (30 to 45) | 0.62 (0.51 to 0.74) | 9.96 (6.42 to 13.62) |
| Qatar | 0 (0 to 0) | 0 (0 to 0) | 0 (0 to 0) | 0 (0 to 0) | 2.35 (1.62 to 3.08) |
| Republic of Nauru | 0 (0 to 0) | 0.01 (0 to 0.01) | 0 (0 to 0) | 0 (0 to 0.01) | −2.75 (−3.38 to −2.12) |
| Republic of Niue | 0 (0 to 0) | 0.01 (0 to 0.01) | 0 (0 to 0) | 0.01 (0.01 to 0.01) | −1.02 (−1.51 to −0.51) |
| Republic of Palau | 0 (0 to 0) | 0 (0 to 0) | 0 (0 to 0) | 0 (0 to 0) | 1.02 (0.90 to 1.13) |
| Republic of San Marino | 0 (0 to 0) | 0.21 (0.12 to 0.28) | 0 (0 to 0) | 0.16 (0.09 to 0.26) | 0.11 (−0.26 to 0.48) |
| Romania | 49 (47 to 51) | 0.22 (0.21 to 0.23) | 96 (83 to 111) | 0.32 (0.28 to 0.36) | 1.77 (1.44 to 2.10) |
| Russian Federation | 108 (81 to 167) | 0.06 (0.05 to 0.10) | 1798 (1644 to 1952) | 0.77 (0.70 to 0.83) | 7.88 (7.09 to 8.67) |
| Rwanda | 0 (0 to 0) | 0 (0 to 0) | 0 (0 to 0) | 0 (0 to 0) | −4.94 (−5.94 to −3.94) |
| Saint Kitts and Nevis | 0 (0 to 0) | 0.02 (0.01 to 0.02) | 1 (0 to 1) | 0.72 (0.56 to 0.87) | 11.73 (8.36 to 15.20) |
| Saint Lucia | 0 (0 to 0) | 0.02 (0.02 to 0.02) | 2 (1 to 2) | 0.80 (0.63 to 1.01) | 10.85 (7.52 to 14.29) |
| Saint Vincent and the Grenadines | 0 (0 to 0) | 0.02 (0.02 to 0.02) | 1 (1 to 1) | 0.47 (0.40 to 0.54) | 8.47 (6.24 to 10.76) |
| Samoa | 0 (0 to 0) | 0 (0 to 0.01) | 0 (0 to 0) | 0 (0 to 0.01) | −1.73 (−2.06 to −1.41) |
| Sao Tome and Principe | 0 (0 to 0) | 0 (0 to 0) | 0 (0 to 0) | 0 (0 to 0.01) | 3.11 (2.99 to 3.23) |
| Saudi Arabia | 1 (1 to 2) | 0.01 (0 to 0.02) | 15 (8 to 23) | 0.05 (0.03 to 0.08) | 5.36 (4.72 to 5.99) |
| Senegal | 0 (0 to 0) | 0 (0 to 0) | 0 (0 to 0) | 0 (0 to 0) | 1.20 (0.94 to 1.47) |
| Serbia | 3 (1 to 4) | 0.03 (0.01 to 0.04) | 7 (5 to 10) | 0.05 (0.03 to 0.06) | 1.89 (1.74 to 2.05) |
| Seychelles | 0 (0 to 0) | 0 (0 to 0) | 0 (0 to 0) | 0.01 (0.01 to 0.01) | 2.86 (2.40 to 3.32) |
| Sierra Leone | 0 (0 to 0) | 0 (0 to 0) | 0 (0 to 0) | 0 (0 to 0) | 1.08 (0.64 to 1.52) |
| Singapore | 10 (10 to 11) | 0.46 (0.43 to 0.48) | 35 (31 to 39) | 0.41 (0.36 to 0.46) | −0.20 (−0.47 to 0.07) |
| Slovakia | 5 (3 to 6) | 0.09 (0.05 to 0.11) | 14 (10 to 17) | 0.17 (0.11 to 0.21) | 2.41 (2.15 to 2.68) |
| Slovenia | 4 (4 to 4) | 0.17 (0.16 to 0.19) | 9 (8 to 11) | 0.24 (0.20 to 0.28) | 1.37 (1.05 to 1.69) |
| Solomon Islands | 0 (0 to 0) | 0 (0 to 0) | 0 (0 to 0) | 0 (0 to 0) | −1.77 (−2.47 to −1.06) |
| Somalia | 0 (0 to 0) | 0 (0 to 0) | 0 (0 to 0) | 0 (0 to 0) | −5.69 (−6.43 to −4.94) |
| South Africa | 4 (1 to 8) | 0.01 (0 to 0.02) | 3 (1 to 7) | 0.01 (0 to 0.01) | −3.05 (−3.21 to −2.89) |
| South Korea | 76 (39 to 88) | 0.25 (0.13 to 0.29) | 246 (156 to 303) | 0.26 (0.17 to 0.33) | 0.32 (−0.36 to 1.00) |
| South Sudan | 0 (0 to 0) | 0 (0 to 0) | 0 (0 to 0) | 0 (0 to 0) | −5.02 (−5.96 to −4.08) |
| Spain | 462 (435 to 486) | 0.91 (0.86 to 0.95) | 1154 (979 to 1360) | 1.28 (1.08 to 1.50) | 1.10 (0.91 to 1.30) |
| Sri Lanka | 1 (1 to 3) | 0.01 (0.01 to 0.03) | 21 (11 to 32) | 0.08 (0.04 to 0.12) | 8.16 (7.26 to 9.07) |
| Sudan | 0 (0 to 0) | 0 (0 to 0) | 0 (0 to 1) | 0 (0 to 0) | 8.66 (7.96 to 9.37) |
| Suriname | 0 (0 to 0) | 0.01 (0.01 to 0.03) | 2 (1 to 3) | 0.31 (0.21 to 0.42) | 10.76 (8.13 to 13.46) |
| Swaziland | 0 (0 to 0) | 0.01 (0 to 0.02) | 0 (0 to 0) | 0.01 (0 to 0.02) | −0.97 (−1.17 to −0.77) |
| Sweden | 188 (176 to 199) | 1.30 (1.23 to 1.37) | 334 (291 to 374) | 1.57 (1.38 to 1.77) | 0.80 (0.54 to 1.06) |
| Switzerland | 146 (135 to 157) | 1.48 (1.36 to 1.58) | 233 (206 to 258) | 1.35 (1.20 to 1.48) | 0.15 (−0.07 to 0.37) |
| Syria | 0 (0 to 0) | 0 (0 to 0) | 0 (0 to 1) | 0 (0 to 0) | 5.97 (5.48 to 6.46) |
| Taiwan (Province of China) | 45 (44 to 47) | 0.25 (0.24 to 0.26) | 160 (130 to 193) | 0.42 (0.35 to 0.50) | 1.11 (0.62 to 1.60) |
| Tajikistan | 0 (0 to 0) | 0 (0 to 0) | 0 (0 to 1) | 0 (0 to 0.01) | 5.54 (4.95 to 6.14) |
| Tanzania | 0 (0 to 0) | 0 (0 to 0) | 0 (0 to 0) | 0 (0 to 0) | −3.74 (−4.55 to −2.93) |
| Thailand | 2 (1 to 4) | 0.01 (0 to 0.01) | 23 (12 to 34) | 0.02 (0.01 to 0.03) | 3.94 (3.62 to 4.26) |
| The Bahamas | 0 (0 to 0) | 0.01 (0 to 0.01) | 2 (2 to 3) | 0.59 (0.45 to 0.76) | 12.24 (8.57 to 16.03) |
| The Gambia | 0 (0 to 0) | 0 (0 to 0) | 0 (0 to 0) | 0 (0 to 0) | 1.31 (1.00 to 1.61) |
| Timor-Leste | 0 (0 to 0) | 0 (0 to 0) | 0 (0 to 0) | 0 (0 to 0.01) | 2.78 (2.56 to 3.00) |
| Togo | 0 (0 to 0) | 0 (0 to 0) | 0 (0 to 0) | 0 (0 to 0) | 0.44 (0.16 to 0.73) |
| Tokelau | 0 (0 to 0) | 0.01 (0 to 0.01) | 0 (0 to 0) | 0.01 (0 to 0.01) | −0.83 (−1.39 to −0.28) |
| Tonga | 0 (0 to 0) | 0 (0 to 0.01) | 0 (0 to 0) | 0 (0 to 0.01) | −1.29 (−1.70 to −0.89) |
| Trinidad and Tobago | 0 (0 to 0) | 0.01 (0.01 to 0.01) | 11 (8 to 15) | 0.60 (0.43 to 0.81) | 11.16 (7.60 to 14.83) |
| Tunisia | 0 (0 to 0) | 0 (0 to 0) | 0 (0 to 1) | 0 (0 to 0.01) | 6.04 (5.67 to 6.41) |
| Turkey | 163 (93 to 283) | 0.32 (0.18 to 0.55) | 616 (412 to 892) | 0.70 (0.48 to 0.99) | 3.01 (2.74 to 3.27) |
| Turkmenistan | 0 (0 to 0) | 0 (0 to 0) | 0 (0 to 0) | 0 (0 to 0) | −0.53 (−2.83 to 1.81) |
| Tuvalu | 0 (0 to 0) | 0 (0 to 0.01) | 0 (0 to 0) | 0 (0 to 0.01) | −0.58 (−0.84 to −0.31) |
| Uganda | 0 (0 to 0) | 0 (0 to 0) | 0 (0 to 0) | 0 (0 to 0) | −1.75 (−2.38 to −1.11) |
| Ukraine | 61 (35 to 95) | 0.10 (0.06 to 0.15) | 133 (95 to 173) | 0.21 (0.15 to 0.27) | 1.72 (1.13 to 2.31) |
| United Arab Emirates | 1 (0 to 2) | 0.11 (0.06 to 0.19) | 9 (6 to 12) | 0.24 (0.17 to 0.34) | 4.01 (3.28 to 4.75) |
| United Kingdom | 1316 (1271 to 1340) | 1.52 (1.47 to 1.54) | 2455 (2242 to 2638) | 1.93 (1.78 to 2.06) | 1.21 (0.87 to 1.55) |
| United States | 3890 (3687 to 4008) | 1.26 (1.20 to 1.29) | 8466 (7800 to 8899) | 1.49 (1.38 to 1.56) | 0.66 (0.33 to 0.99) |
| Uruguay | 3 (3 to 3) | 0.08 (0.08 to 0.09) | 65 (58 to 72) | 1.28 (1.13 to 1.41) | 7.22 (5.39 to 9.08) |
| Uzbekistan | 0 (0 to 0) | 0 (0 to 0) | 0 (0 to 0) | 0 (0 to 0) | 8.71 (7.84 to 9.58) |
| Vanuatu | 0 (0 to 0) | 0 (0 to 0) | 0 (0 to 0) | 0 (0 to 0) | −1.55 (−2.10 to −1.00) |
| Venezuela | 13 (13 to 14) | 0.12 (0.11 to 0.12) | 112 (74 to 155) | 0.37 (0.24 to 0.50) | 3.77 (3.22 to 4.32) |
| Vietnam | 3 (1 to 6) | 0.01 (0 to 0.01) | 15 (7 to 28) | 0.01 (0.01 to 0.03) | 2.71 (2.58 to 2.84) |
| Virgin Islands, U.S. | 0 (0 to 0) | 0.18 (0.14 to 0.25) | 1 (1 to 1) | 0.58 (0.42 to 0.78) | 3.81 (2.82 to 4.81) |
| Yemen | 0 (0 to 0) | 0 (0 to 0) | 0 (0 to 0) | 0 (0 to 0) | 10.11 (9.37 to 10.86) |
| Zambia | 0 (0 to 0) | 0 (0 to 0) | 0 (0 to 0) | 0 (0 to 0) | −3.52 (−4.46 to −2.57) |
| Zimbabwe | 1 (0 to 2) | 0.02 (0.01 to 0.03) | 2 (1 to 3) | 0.02 (0.01 to 0.04) | −0.12 (−0.57 to 0.32) |

Abbreviations: ASR, age-standardized rate; MND, motor neuron disease; EAPC, estimated annual percentage change; SDI, sociodemographic index; UI, uncertainty interval; CI, confidence interval.

**Table S7.** The case number and ASR of DALYs of MND in 1990 and 2021, with EAPC from 1990 to 2021.

|  | **1990** | | **2021** | | **EAPC (95% CI)** |
| --- | --- | --- | --- | --- | --- |
|  | **Number (95% UI)** | **ASR (95% UI)** | **Number (95% UI)** | **ASR (95% UI)** |  |
| **Sex** |  |  |  |  |  |
| Female | 230898 (219091 to 243419) | 9.86 (9.38 to 10.36) | 448517 (413228 to 496721) | 10.08 (9.33 to 11.14) | 0.10 (0.05 to 0.16) |
| Male | 275249 (230289 to 305186) | 12.73 (11 to 13.82) | 592049 (529600 to 642811) | 14.45 (12.93 to 15.76) | 0.45 (0.36 to 0.54) |
| **Age** |  |  |  |  |  |
| <5 years | 95798 (77178 to 115093) | 15.45 (12.45 to 18.57) | 40610 (32291 to 50714) | 6.17 (4.91 to 7.71) | −2.99 (−3.15 to −2.83) |
| 5-9 years | 6546 (5282 to 7769) | 1.12 (0.91 to 1.33) | 6303 (5273 to 7607) | 0.92 (0.77 to 1.11) | −0.71 (−0.83 to −0.59) |
| 10-14 years | 6712 (5264 to 8224) | 1.25 (0.98 to 1.54) | 7550 (6207 to 9350) | 1.13 (0.93 to 1.40) | −0.56 (−0.72 to −0.41) |
| 15-19 years | 11615 (7417 to 14748) | 2.24 (1.43 to 2.84) | 11088 (8529 to 13568) | 1.78 (1.37 to 2.17) | −1.04 (−1.19 to −0.90) |
| 20-24 years | 9116 (6330 to 11514) | 1.85 (1.29 to 2.34) | 10702 (8598 to 13043) | 1.79 (1.44 to 2.18) | −0.33 (−0.47 to −0.19) |
| 25-29 years | 7420 (6096 to 9182) | 1.68 (1.38 to 2.07) | 10649 (8887 to 12611) | 1.81 (1.51 to 2.14) | 0.19 (0.08 to 0.29) |
| 30-34 years | 9189 (8009 to 10689) | 2.38 (2.08 to 2.77) | 15800 (13540 to 18366) | 2.61 (2.24 to 3.04) | 0.11 (0.01 to 0.22) |
| 35-39 years | 13010 (11554 to 14614) | 3.69 (3.28 to 4.15) | 23415 (20888 to 25899) | 4.17 (3.72 to 4.62) | 0.04 (−0.11 to 0.19) |
| 40-44 years | 18692 (17038 to 20404) | 6.52 (5.95 to 7.12) | 36108 (31790 to 39730) | 7.22 (6.35 to 7.94) | 0.03 (−0.15 to 0.21) |
| 45-49 years | 24361 (22547 to 26341) | 10.49 (9.71 to 11.34) | 56247 (50620 to 61625) | 11.88 (10.69 to 13.01) | 0.25 (0.07 to 0.44) |
| 50-54 years | 35798 (33247 to 38425) | 16.84 (15.64 to 18.08) | 88692 (80809 to 96788) | 19.93 (18.16 to 21.75) | 0.49 (0.30 to 0.69) |
| 55-59 years | 47872 (44720 to 51013) | 25.85 (24.15 to 27.55) | 122758 (113911 to 132895) | 31.02 (28.79 to 33.58) | 0.74 (0.59 to 0.90) |
| 60-64 years | 58695 (55850 to 61774) | 36.55 (34.77 to 38.46) | 146696 (136876 to 157470) | 45.84 (42.77 to 49.20) | 0.90 (0.80 to 1.01) |
| 65-69 years | 62759 (60181 to 65469) | 50.77 (48.69 to 52.96) | 156374 (145377 to 169081) | 56.69 (52.70 to 61.30) | 0.71 (0.60 to 0.83) |
| 70-74 years | 44003 (42076 to 45701) | 51.98 (49.70 to 53.98) | 140469 (129189 to 152885) | 68.24 (62.76 to 74.27) | 0.78 (0.71 to 0.85) |
| 75-79 years | 33034 (31228 to 34356) | 53.67 (50.73 to 55.81) | 91537 (81843 to 100417) | 69.41 (62.06 to 76.14) | 0.87 (0.67 to 1.07) |
| 80-84 years | 14576 (13038 to 15425) | 41.20 (36.85 to 43.60) | 48323 (40379 to 53679) | 55.17 (46.10 to 61.29) | 1.09 (0.77 to 1.40) |
| 85-89 years | 5654 (4868 to 6168) | 37.42 (32.22 to 40.81) | 19512 (15932 to 22002) | 42.68 (34.84 to 48.12) | 1.06 (0.75 to 1.36) |
| 90-94 years | 1096 (916 to 1203) | 25.57 (21.39 to 28.06) | 6043 (4767 to 6907) | 33.78 (26.65 to 38.61) | 1.42 (1.14 to 1.70) |
| 95+ years | 202 (161 to 229) | 19.88 (15.83 to 22.45) | 1689 (1271 to 1959) | 30.98 (23.31 to 35.94) | 1.69 (1.48 to 1.89) |
| **Country** |  |  |  |  |  |
| Afghanistan | 38 (26 to 54) | 0.41 (0.28 to 0.58) | 123 (82 to 177) | 0.41 (0.28 to 0.57) | 0.07 (0 to 0.15) |
| Albania | 167 (112 to 238) | 4.73 (3.26 to 6.61) | 119 (77 to 185) | 4.73 (3.01 to 8.13) | 0.21 (0.07 to 0.36) |
| Algeria | 133 (90 to 191) | 0.55 (0.37 to 0.77) | 255 (175 to 359) | 0.58 (0.40 to 0.82) | 0.29 (0.25 to 0.34) |
| American Samoa | 0 (0 to 0) | 0.74 (0.54 to 0.98) | 0 (0 to 0) | 0.44 (0.31 to 0.64) | −2.29 (−2.67 to −1.91) |
| Andorra | 22 (12 to 33) | 41.38 (23 to 63.48) | 54 (33 to 79) | 39.45 (24.53 to 57.53) | 0.44 (0.23 to 0.65) |
| Angola | 31 (23 to 44) | 0.34 (0.25 to 0.47) | 89 (60 to 129) | 0.31 (0.22 to 0.44) | −0.32 (−0.42 to −0.22) |
| Antigua and Barbuda | 1 (0 to 1) | 0.94 (0.75 to 1.17) | 15 (13 to 17) | 14.57 (13.01 to 16.11) | 8.47 (6.07 to 10.93) |
| Argentina | 791 (703 to 890) | 2.41 (2.14 to 2.71) | 10139 (9242 to 11118) | 19.71 (17.95 to 21.57) | 6.18 (4.52 to 7.87) |
| Armenia | 106 (77 to 168) | 3.09 (2.24 to 4.88) | 172 (140 to 209) | 5.09 (4.08 to 6.15) | 1.56 (0.94 to 2.18) |
| Australia | 8500 (8135 to 8880) | 46.84 (44.81 to 48.92) | 19400 (17505 to 21306) | 50.51 (46.04 to 55.21) | 0.52 (0.23 to 0.80) |
| Austria | 2558 (2440 to 2681) | 27.62 (26.40 to 28.77) | 4689 (4360 to 5010) | 31.85 (29.85 to 34.14) | 0.89 (0.70 to 1.08) |
| Azerbaijan | 46 (33 to 63) | 0.63 (0.45 to 0.86) | 94 (64 to 133) | 0.90 (0.61 to 1.30) | 1.68 (1.45 to 1.91) |
| Bahrain | 4 (3 to 5) | 0.76 (0.56 to 0.99) | 12 (8 to 16) | 0.77 (0.56 to 1.04) | 0.23 (0.13 to 0.33) |
| Bangladesh | 405 (291 to 551) | 0.40 (0.28 to 0.55) | 1280 (747 to 1940) | 0.82 (0.47 to 1.26) | 2.33 (2.14 to 2.52) |
| Barbados | 3 (3 to 4) | 1.33 (1.14 to 1.58) | 154 (107 to 220) | 35.64 (24.96 to 51.14) | 10.50 (7.60 to 13.48) |
| Belarus | 701 (489 to 1331) | 6.29 (4.56 to 11.41) | 3023 (2448 to 3691) | 21.39 (17.32 to 25.84) | 3.73 (3.25 to 4.22) |
| Belgium | 8365 (7895 to 8838) | 61.59 (58.67 to 64.52) | 9536 (8533 to 10636) | 52.12 (46.96 to 57.89) | 0.60 (0.05 to 1.16) |
| Belize | 1 (1 to 2) | 0.69 (0.54 to 0.89) | 35 (29 to 41) | 9.52 (7.86 to 11.15) | 8.04 (5.65 to 10.48) |
| Benin | 14 (10 to 19) | 0.31 (0.22 to 0.42) | 39 (27 to 54) | 0.31 (0.23 to 0.43) | 0.02 (−0.04 to 0.08) |
| Bermuda | 1 (1 to 2) | 2.14 (1.61 to 3.00) | 15 (12 to 19) | 14.84 (11.52 to 19.06) | 5.86 (4.16 to 7.58) |
| Bhutan | 2 (2 to 3) | 0.41 (0.29 to 0.56) | 6 (3 to 8) | 0.77 (0.44 to 1.19) | 2.31 (2.19 to 2.42) |
| Bolivia | 30 (22 to 41) | 0.58 (0.41 to 0.79) | 440 (256 to 606) | 4.30 (2.52 to 6.00) | 7.51 (6.96 to 8.06) |
| Bosnia and Herzegovina | 324 (180 to 430) | 7.33 (4.10 to 9.91) | 505 (306 to 689) | 11.20 (6.87 to 16.20) | 1.70 (1.53 to 1.87) |
| Botswana | 10 (7 to 14) | 0.83 (0.52 to 1.20) | 16 (9 to 27) | 0.63 (0.40 to 1.02) | −1.08 (−1.21 to −0.96) |
| Brazil | 13138 (12654 to 13791) | 11.13 (10.76 to 11.58) | 56678 (53150 to 60580) | 22.76 (21.34 to 24.31) | 2.60 (2.47 to 2.73) |
| Brunei | 11 (4 to 19) | 6.82 (2.58 to 12.36) | 45 (22 to 62) | 11.33 (5.45 to 15.10) | 1.98 (1.86 to 2.09) |
| Bulgaria | 371 (336 to 407) | 4.35 (3.95 to 4.78) | 626 (522 to 748) | 7.00 (5.88 to 8.32) | 1.81 (1.56 to 2.05) |
| Burkina Faso | 24 (17 to 34) | 0.28 (0.20 to 0.39) | 59 (41 to 83) | 0.29 (0.20 to 0.40) | 0.01 (−0.03 to 0.06) |
| Burundi | 13 (9 to 18) | 0.27 (0.19 to 0.37) | 27 (18 to 40) | 0.23 (0.16 to 0.32) | −0.57 (−0.66 to −0.48) |
| Cambodia | 36 (25 to 50) | 0.38 (0.27 to 0.56) | 79 (53 to 115) | 0.49 (0.33 to 0.72) | 0.81 (0.73 to 0.88) |
| Cameroon | 33 (25 to 44) | 0.34 (0.26 to 0.45) | 109 (80 to 148) | 0.37 (0.28 to 0.50) | 0.22 (0.13 to 0.32) |
| Canada | 12214 (11646 to 12705) | 40.94 (39.03 to 42.53) | 26641 (24704 to 28502) | 43.56 (40.44 to 46.53) | 0.22 (0.02 to 0.43) |
| Cape Verde | 1 (1 to 2) | 0.40 (0.28 to 0.55) | 3 (2 to 3) | 0.44 (0.32 to 0.60) | 0.33 (0.31 to 0.35) |
| Central African Republic | 7 (5 to 9) | 0.27 (0.20 to 0.37) | 11 (7 to 16) | 0.23 (0.15 to 0.31) | −0.69 (−0.77 to −0.60) |
| Chad | 16 (11 to 21) | 0.29 (0.20 to 0.40) | 47 (32 to 66) | 0.30 (0.21 to 0.42) | 0.15 (0.11 to 0.19) |
| Chile | 632 (592 to 681) | 5.55 (5.21 to 5.95) | 6284 (5687 to 6944) | 26.35 (23.88 to 29.04) | 3.59 (2.67 to 4.52) |
| China | 87565 (50251 to 111668) | 7.99 (4.67 to 10.15) | 122662 (81014 to 167333) | 7.67 (4.88 to 10.06) | −1.51 (−2.11 to −0.91) |
| Colombia | 2046 (1943 to 2147) | 8.28 (7.91 to 8.63) | 9255 (7582 to 11024) | 17.30 (14.14 to 20.63) | 2.75 (2.52 to 2.99) |
| Comoros | 1 (1 to 2) | 0.35 (0.25 to 0.48) | 2 (2 to 3) | 0.31 (0.21 to 0.44) | −0.49 (−0.57 to −0.41) |
| Congo | 8 (5 to 11) | 0.36 (0.26 to 0.48) | 14 (10 to 20) | 0.29 (0.20 to 0.40) | −0.97 (−1.18 to −0.76) |
| Cook Islands | 0 (0 to 0) | 0.71 (0.51 to 0.99) | 0 (0 to 0) | 0.63 (0.45 to 0.86) | −0.73 (−0.86 to −0.61) |
| Costa Rica | 227 (215 to 238) | 10.43 (9.90 to 10.91) | 1734 (1509 to 1977) | 32.29 (28.06 to 36.78) | 3.74 (3.38 to 4.10) |
| Cote d'Ivoire | 36 (26 to 49) | 0.32 (0.24 to 0.43) | 86 (62 to 119) | 0.33 (0.24 to 0.44) | −0.01 (−0.08 to 0.06) |
| Croatia | 919 (859 to 989) | 17.75 (16.67 to 18.94) | 1620 (1362 to 1971) | 26.04 (22.10 to 31.28) | 1.88 (1.61 to 2.16) |
| Cuba | 108 (86 to 135) | 1.00 (0.81 to 1.25) | 3456 (2889 to 4066) | 20.92 (17.62 to 24.58) | 9.19 (6.56 to 11.88) |
| Cyprus | 155 (71 to 254) | 19.77 (9.12 to 32.05) | 446 (293 to 611) | 23.42 (15.50 to 32.03) | 0.41 (0.11 to 0.70) |
| Czech Republic | 1846 (1703 to 2010) | 16.80 (15.61 to 18.14) | 3343 (2880 to 3878) | 21.65 (18.70 to 25.10) | 1.52 (1.16 to 1.89) |
| Democratic Republic of the Congo | 103 (74 to 144) | 0.30 (0.21 to 0.40) | 192 (128 to 277) | 0.24 (0.16 to 0.33) | −0.76 (−0.96 to −0.56) |
| Denmark | 2661 (2446 to 2883) | 40.88 (37.67 to 44.33) | 4400 (3976 to 4855) | 43.41 (39.34 to 47.61) | 0.25 (0.10 to 0.39) |
| Djibouti | 1 (1 to 2) | 0.37 (0.26 to 0.50) | 4 (3 to 6) | 0.32 (0.22 to 0.45) | −0.54 (−0.63 to −0.44) |
| Dominica | 1 (0 to 1) | 0.91 (0.68 to 1.53) | 16 (11 to 21) | 22.44 (15.7 to 30.34) | 10.58 (8.63 to 12.56) |
| Dominican Republic | 40 (28 to 57) | 0.57 (0.40 to 0.83) | 74 (51 to 117) | 0.68 (0.47 to 1.08) | 0.50 (0.09 to 0.92) |
| Ecuador | 31 (21 to 45) | 0.34 (0.23 to 0.47) | 2489 (1926 to 3155) | 14.67 (11.35 to 18.58) | 13.69 (10.69 to 16.78) |
| Egypt | 550 (415 to 740) | 0.89 (0.68 to 1.17) | 488 (322 to 699) | 0.47 (0.31 to 0.66) | −2.99 (−3.54 to −2.44) |
| El Salvador | 80 (55 to 95) | 1.80 (1.21 to 2.10) | 239 (165 to 308) | 3.92 (2.70 to 5.08) | 2.76 (2.67 to 2.86) |
| Equatorial Guinea | 1 (1 to 2) | 0.29 (0.21 to 0.39) | 4 (3 to 6) | 0.32 (0.23 to 0.46) | 0.37 (0.33 to 0.41) |
| Eritrea | 10 (7 to 14) | 0.32 (0.23 to 0.45) | 19 (12 to 26) | 0.30 (0.21 to 0.42) | −0.34 (−0.39 to −0.28) |
| Estonia | 237 (201 to 297) | 13.11 (11.21 to 16.24) | 510 (441 to 585) | 24.68 (21.36 to 28.29) | 2.96 (0.70 to 5.27) |
| Ethiopia | 131 (88 to 187) | 0.29 (0.20 to 0.40) | 272 (171 to 400) | 0.27 (0.18 to 0.38) | −0.28 (−0.39 to −0.16) |
| Federated States of Micronesia | 0 (0 to 1) | 0.41 (0.29 to 0.56) | 0 (0 to 0) | 0.35 (0.25 to 0.48) | −0.70 (−0.78 to −0.62) |
| Fiji | 3 (2 to 4) | 0.41 (0.29 to 0.56) | 4 (3 to 6) | 0.48 (0.35 to 0.64) | 0.63 (0.45 to 0.81) |
| Finland | 3270 (3128 to 3439) | 51.66 (49.47 to 54.10) | 5875 (4861 to 7071) | 58.07 (48.93 to 68.42) | 0.58 (0.45 to 0.71) |
| France | 30745 (29363 to 32020) | 45.83 (44.19 to 47.64) | 50543 (43537 to 58163) | 47.40 (41.46 to 53.99) | 0.34 (0.26 to 0.41) |
| Gabon | 4 (3 to 5) | 0.39 (0.29 to 0.52) | 5 (4 to 8) | 0.31 (0.22 to 0.44) | −0.97 (−1.14 to −0.81) |
| Georgia | 39 (29 to 53) | 0.71 (0.52 to 0.96) | 352 (297 to 418) | 8.12 (6.89 to 9.61) | 10.87 (9.18 to 12.58) |
| Germany | 35385 (33960 to 36793) | 35.08 (33.71 to 36.49) | 56597 (52429 to 60390) | 37.44 (35.13 to 39.79) | 0.60 (0.41 to 0.78) |
| Ghana | 51 (39 to 68) | 0.37 (0.28 to 0.49) | 142 (104 to 190) | 0.44 (0.33 to 0.58) | 0.53 (0.42 to 0.64) |
| Greece | 1644 (1560 to 1726) | 13.35 (12.73 to 13.92) | 5123 (4587 to 5729) | 28.84 (25.91 to 32.15) | 2.73 (2.61 to 2.84) |
| Greenland | 5 (2 to 6) | 11.32 (4.67 to 14.29) | 8 (3 to 10) | 10.65 (4.63 to 13.95) | 0.16 (0.05 to 0.27) |
| Grenada | 1 (1 to 1) | 0.88 (0.73 to 1.06) | 17 (14 to 20) | 14.98 (12.52 to 17.02) | 9.35 (6.95 to 11.80) |
| Guam | 4 (2 to 5) | 3.47 (2.22 to 4.47) | 1 (1 to 1) | 0.47 (0.33 to 0.69) | −7.38 (−8.31 to −6.44) |
| Guatemala | 157 (145 to 171) | 2.21 (2.06 to 2.39) | 581 (495 to 670) | 4.49 (3.81 to 5.21) | 2.52 (2.31 to 2.74) |
| Guinea | 17 (12 to 23) | 0.30 (0.21 to 0.41) | 38 (27 to 52) | 0.31 (0.22 to 0.42) | 0.07 (0 to 0.14) |
| Guinea-Bissau | 3 (2 to 4) | 0.31 (0.23 to 0.42) | 6 (4 to 8) | 0.32 (0.24 to 0.43) | 0.02 (−0.03 to 0.08) |
| Guyana | 5 (4 to 6) | 0.67 (0.56 to 0.83) | 124 (90 to 161) | 16.85 (12.30 to 21.90) | 9.98 (7.39 to 12.63) |
| Haiti | 47 (32 to 70) | 0.74 (0.49 to 1.17) | 408 (220 to 688) | 3.58 (1.82 to 6.31) | 5.75 (5.25 to 6.26) |
| Honduras | 111 (88 to 138) | 2.98 (2.36 to 3.74) | 619 (413 to 886) | 8.18 (5.26 to 11.95) | 3.48 (3.40 to 3.56) |
| Hungary | 2188 (2039 to 2357) | 20.03 (18.89 to 21.43) | 4011 (3456 to 4631) | 28.79 (25.02 to 32.87) | 1.74 (1.42 to 2.06) |
| Iceland | 87 (82 to 92) | 32.79 (30.73 to 34.81) | 178 (160 to 198) | 35.96 (32.44 to 39.83) | 0.53 (0.36 to 0.70) |
| India | 3809 (2729 to 5225) | 0.48 (0.34 to 0.69) | 14693 (9550 to 19885) | 1.10 (0.70 to 1.49) | 2.75 (2.55 to 2.94) |
| Indonesia | 904 (667 to 1194) | 0.54 (0.40 to 0.72) | 2008 (1394 to 2879) | 0.72 (0.50 to 1.03) | 0.68 (0.56 to 0.80) |
| Iran | 536 (403 to 688) | 1.05 (0.81 to 1.36) | 1509 (1194 to 1898) | 1.79 (1.42 to 2.24) | 2.20 (2.02 to 2.38) |
| Iraq | 98 (66 to 140) | 0.55 (0.37 to 0.78) | 227 (151 to 323) | 0.55 (0.36 to 0.77) | 0.06 (0.01 to 0.12) |
| Ireland | 1895 (1797 to 1993) | 50.05 (47.64 to 52.55) | 3976 (3289 to 4648) | 56.39 (46.55 to 65.89) | 0.68 (0.55 to 0.81) |
| Israel | 1267 (1209 to 1326) | 25.93 (24.78 to 27.07) | 2836 (2563 to 3114) | 25.29 (22.86 to 27.72) | 0.17 (0.03 to 0.31) |
| Italy | 21338 (20621 to 21961) | 33.15 (32.19 to 34.06) | 39784 (35726 to 44493) | 37.51 (34.03 to 41.73) | 0.48 (0.25 to 0.71) |
| Jamaica | 19 (15 to 24) | 0.86 (0.68 to 1.07) | 463 (333 to 623) | 15.86 (11.44 to 21.29) | 9.12 (6.48 to 11.84) |
| Japan | 28490 (27548 to 29383) | 17.67 (17.12 to 18.23) | 60949 (54462 to 66334) | 22.05 (20.37 to 23.71) | 0.76 (0.61 to 0.90) |
| Jordan | 52 (37 to 69) | 1.36 (0.97 to 1.84) | 278 (195 to 362) | 2.56 (1.79 to 3.38) | 2.89 (2.50 to 3.28) |
| Kazakhstan | 103 (71 to 144) | 0.63 (0.43 to 0.88) | 121 (83 to 166) | 0.65 (0.44 to 0.89) | 0.13 (0.02 to 0.24) |
| Kenya | 67 (46 to 97) | 0.32 (0.23 to 0.45) | 135 (88 to 195) | 0.28 (0.19 to 0.39) | −0.56 (−0.67 to −0.44) |
| Kiribati | 2 (1 to 2) | 3.40 (2.51 to 4.69) | 6 (4 to 8) | 6.01 (4.28 to 8.44) | 1.96 (1.79 to 2.13) |
| Kuwait | 221 (190 to 249) | 15.55 (13.44 to 17.45) | 370 (284 to 462) | 9.94 (7.55 to 12.54) | 0.16 (−1.63 to 1.98) |
| Kyrgyzstan | 27 (19 to 36) | 0.60 (0.42 to 0.81) | 357 (301 to 419) | 5.44 (4.58 to 6.42) | 9.56 (8.08 to 11.07) |
| Laos | 15 (10 to 20) | 0.38 (0.27 to 0.53) | 36 (24 to 57) | 0.52 (0.35 to 0.84) | 1.06 (0.98 to 1.15) |
| Latvia | 99 (82 to 130) | 3.29 (2.76 to 4.30) | 768 (659 to 882) | 25.43 (21.93 to 29.00) | 7.54 (6.20 to 8.89) |
| Lebanon | 72 (42 to 126) | 2.38 (1.38 to 4.13) | 264 (175 to 342) | 4.97 (3.29 to 6.61) | 3.08 (2.88 to 3.28) |
| Lesotho | 8 (6 to 11) | 0.53 (0.38 to 0.74) | 9 (6 to 13) | 0.49 (0.33 to 0.71) | −0.25 (−0.32 to −0.18) |
| Liberia | 7 (5 to 9) | 0.30 (0.22 to 0.40) | 15 (10 to 20) | 0.29 (0.21 to 0.38) | −0.04 (−0.17 to 0.10) |
| Libya | 22 (15 to 31) | 0.55 (0.37 to 0.76) | 41 (29 to 57) | 0.60 (0.43 to 0.82) | 0.43 (0.36 to 0.49) |
| Lithuania | 620 (527 to 767) | 15.3 (13.18 to 18.8) | 2011 (1726 to 2261) | 45.68 (39.45 to 51.29) | 3.69 (2.31 to 5.09) |
| Luxembourg | 159 (150 to 168) | 34.18 (32.34 to 36.2) | 282 (250 to 322) | 30.53 (27.01 to 34.88) | −0.01 (−0.18 to 0.16) |
| Macedonia | 170 (107 to 227) | 9.37 (5.81 to 12.77) | 201 (128 to 283) | 7.49 (4.63 to 10.88) | −0.07 (−0.27 to 0.13) |
| Madagascar | 40 (28 to 55) | 0.38 (0.27 to 0.51) | 84 (55 to 121) | 0.33 (0.22 to 0.46) | −0.48 (−0.57 to −0.38) |
| Malawi | 28 (20 to 39) | 0.32 (0.23 to 0.44) | 50 (33 to 73) | 0.29 (0.19 to 0.40) | −0.39 (−0.46 to −0.31) |
| Malaysia | 118 (74 to 171) | 0.80 (0.48 to 1.17) | 303 (204 to 447) | 0.96 (0.65 to 1.42) | 0.37 (0.24 to 0.51) |
| Maldives | 3 (2 to 6) | 1.83 (1.02 to 3.29) | 30 (17 to 47) | 7.05 (4.11 to 10.39) | 4.63 (4.25 to 5.01) |
| Mali | 31 (23 to 42) | 0.45 (0.32 to 0.60) | 87 (61 to 118) | 0.48 (0.34 to 0.63) | 0.16 (0.12 to 0.21) |
| Malta | 116 (108 to 123) | 28.83 (26.89 to 30.7) | 266 (231 to 309) | 36.35 (31.36 to 42.14) | 0.89 (0.73 to 1.06) |
| Marshall Islands | 0 (0 to 0) | 0.39 (0.28 to 0.53) | 0 (0 to 0) | 0.37 (0.27 to 0.49) | −0.41 (−0.49 to −0.34) |
| Mauritania | 8 (6 to 11) | 0.42 (0.30 to 0.56) | 17 (12 to 24) | 0.43 (0.32 to 0.58) | 0.01 (−0.06 to 0.09) |
| Mauritius | 23 (21 to 26) | 2.47 (2.30 to 2.68) | 365 (328 to 403) | 21.75 (19.39 to 23.86) | 7.66 (5.48 to 9.87) |
| Mexico | 5131 (4892 to 5387) | 7.73 (7.48 to 8.00) | 19104 (16634 to 21760) | 14.35 (12.51 to 16.33) | 1.95 (1.79 to 2.12) |
| Moldova | 536 (501 to 577) | 11.88 (11.11 to 12.8) | 674 (589 to 762) | 13.09 (11.34 to 14.82) | 2.59 (−0.03 to 5.28) |
| Mongolia | 98 (37 to 200) | 4.14 (1.60 to 8.01) | 121 (82 to 189) | 3.73 (2.52 to 5.73) | −0.97 (−1.27 to −0.67) |
| Montenegro | 7 (6 to 9) | 1.19 (0.95 to 1.52) | 8 (7 to 10) | 1.20 (0.96 to 1.51) | 0.30 (0.23 to 0.37) |
| Morocco | 129 (87 to 187) | 0.52 (0.35 to 0.74) | 207 (141 to 292) | 0.55 (0.38 to 0.78) | 0.25 (0.20 to 0.29) |
| Mozambique | 39 (28 to 55) | 0.32 (0.23 to 0.45) | 85 (55 to 123) | 0.31 (0.21 to 0.44) | −0.14 (−0.20 to −0.07) |
| Myanmar | 166 (116 to 233) | 0.44 (0.31 to 0.63) | 349 (246 to 489) | 0.63 (0.45 to 0.89) | 1.26 (1.21 to 1.31) |
| Namibia | 10 (7 to 14) | 0.76 (0.51 to 1.04) | 15 (9 to 26) | 0.62 (0.39 to 1.06) | −0.78 (−0.95 to −0.61) |
| Nepal | 71 (49 to 99) | 0.38 (0.27 to 0.53) | 196 (116 to 291) | 0.67 (0.39 to 1.02) | 1.92 (1.74 to 2.10) |
| Netherlands | 8028 (7669 to 8348) | 46.99 (45.06 to 48.82) | 14812 (13466 to 16268) | 50.98 (46.68 to 55.84) | 0.50 (0.33 to 0.66) |
| New Zealand | 1781 (1691 to 1877) | 47.33 (45.06 to 49.83) | 3358 (3002 to 3724) | 44.63 (39.97 to 49.49) | 0.02 (−0.34 to 0.38) |
| Nicaragua | 60 (40 to 77) | 1.82 (1.24 to 2.34) | 206 (133 to 269) | 3.61 (2.28 to 4.76) | 2.59 (2.39 to 2.79) |
| Niger | 21 (14 to 29) | 0.30 (0.20 to 0.41) | 61 (40 to 86) | 0.28 (0.19 to 0.39) | −0.15 (−0.20 to −0.11) |
| Nigeria | 292 (210 to 402) | 0.34 (0.25 to 0.47) | 813 (588 to 1103) | 0.38 (0.28 to 0.52) | 0.47 (0.34 to 0.60) |
| North Korea | 859 (478 to 1457) | 3.98 (2.23 to 6.69) | 913 (503 to 1808) | 3.16 (1.70 to 6.16) | −0.65 (−0.87 to −0.43) |
| Northern Mariana Islands | 0 (0 to 0) | 0.57 (0.41 to 0.78) | 0 (0 to 0) | 0.49 (0.34 to 0.70) | −0.67 (−0.92 to −0.43) |
| Norway | 2425 (2340 to 2502) | 43.54 (42.13 to 44.89) | 3919 (3660 to 4190) | 45.15 (42.26 to 48.13) | 0.14 (−0.03 to 0.30) |
| Oman | 13 (9 to 17) | 0.66 (0.48 to 0.89) | 78 (62 to 98) | 2.15 (1.70 to 2.68) | 5.00 (4.50 to 5.51) |
| Pakistan | 513 (359 to 705) | 0.48 (0.33 to 0.68) | 2275 (1439 to 3129) | 1.11 (0.66 to 1.58) | 2.88 (2.83 to 2.93) |
| Palestine | 39 (27 to 55) | 1.59 (1.18 to 2.29) | 135 (103 to 180) | 2.96 (2.31 to 3.74) | 2.59 (2.34 to 2.84) |
| Panama | 91 (85 to 98) | 4.83 (4.52 to 5.15) | 602 (471 to 733) | 13.91 (10.89 to 16.95) | 3.54 (3.36 to 3.73) |
| Papua New Guinea | 12 (8 to 17) | 0.30 (0.21 to 0.42) | 29 (19 to 40) | 0.28 (0.19 to 0.39) | −0.31 (−0.37 to −0.25) |
| Paraguay | 38 (30 to 49) | 1.09 (0.85 to 1.37) | 138 (99 to 173) | 2.12 (1.50 to 2.67) | 2.36 (2.31 to 2.41) |
| Peru | 100 (69 to 136) | 0.54 (0.37 to 0.74) | 2034 (1206 to 2817) | 5.84 (3.48 to 8.11) | 8.45 (7.51 to 9.40) |
| Philippines | 493 (321 to 634) | 0.98 (0.60 to 1.27) | 1010 (762 to 1239) | 0.98 (0.74 to 1.19) | −0.05 (−0.47 to 0.36) |
| Poland | 3913 (3764 to 4070) | 10.77 (10.35 to 11.22) | 17210 (15596 to 18881) | 31.51 (28.67 to 34.48) | 3.52 (2.93 to 4.12) |
| Portugal | 2789 (2668 to 2938) | 25.68 (24.58 to 26.89) | 5999 (5375 to 6709) | 31.76 (28.50 to 35.37) | 0.75 (0.65 to 0.84) |
| Principality of Monaco | 4 (3 to 5) | 7.81 (5.90 to 10.25) | 9 (6 to 11) | 12.98 (9.54 to 17.90) | 1.52 (1.25 to 1.79) |
| Puerto Rico | 45 (37 to 55) | 1.24 (1.03 to 1.52) | 1003 (834 to 1205) | 20.57 (17.27 to 24.51) | 8.10 (5.39 to 10.88) |
| Qatar | 3 (2 to 4) | 0.65 (0.45 to 0.90) | 21 (14 to 30) | 0.65 (0.45 to 0.92) | 0.16 (0.12 to 0.20) |
| Republic of Nauru | 0 (0 to 0) | 0.52 (0.37 to 0.70) | 0 (0 to 0) | 0.38 (0.28 to 0.50) | −1.24 (−1.50 to −0.98) |
| Republic of Niue | 0 (0 to 0) | 0.69 (0.51 to 0.94) | 0 (0 to 0) | 0.89 (0.66 to 1.24) | −0.30 (−0.69 to 0.09) |
| Republic of Palau | 0 (0 to 0) | 0.40 (0.28 to 0.55) | 0 (0 to 0) | 0.39 (0.28 to 0.53) | −0.16 (−0.19 to −0.12) |
| Republic of San Marino | 2 (1 to 3) | 7.85 (4.98 to 10.23) | 3 (2 to 5) | 5.89 (3.64 to 8.77) | −0.24 (−0.52 to 0.03) |
| Romania | 2690 (2523 to 2856) | 13.63 (12.65 to 14.60) | 3047 (2654 to 3455) | 12.88 (11.35 to 14.47) | 0.47 (0.11 to 0.83) |
| Russian Federation | 4915 (3861 to 7012) | 3.06 (2.45 to 4.32) | 52302 (48059 to 56523) | 24.41 (22.54 to 26.27) | 6.44 (5.76 to 7.13) |
| Rwanda | 18 (12 to 25) | 0.27 (0.19 to 0.37) | 30 (20 to 43) | 0.24 (0.16 to 0.34) | −0.43 (−0.56 to −0.30) |
| Saint Kitts and Nevis | 0 (0 to 0) | 1.04 (0.88 to 1.24) | 16 (13 to 20) | 22.76 (17.80 to 27.95) | 9.41 (6.89 to 11.98) |
| Saint Lucia | 1 (1 to 2) | 1.09 (0.93 to 1.29) | 58 (45 to 73) | 26.78 (20.66 to 34.09) | 9.34 (6.66 to 12.08) |
| Saint Vincent and the Grenadines | 1 (1 to 1) | 1.17 (1.02 to 1.35) | 21 (18 to 25) | 15.92 (13.41 to 18.73) | 7.33 (5.44 to 9.25) |
| Samoa | 1 (1 to 1) | 0.48 (0.34 to 0.66) | 1 (1 to 1) | 0.42 (0.29 to 0.57) | −0.57 (−0.65 to −0.49) |
| Sao Tome and Principe | 0 (0 to 1) | 0.33 (0.24 to 0.44) | 1 (1 to 1) | 0.46 (0.34 to 0.59) | 1.20 (1.12 to 1.28) |
| Saudi Arabia | 156 (106 to 238) | 0.98 (0.65 to 1.52) | 837 (531 to 1159) | 2.40 (1.53 to 3.37) | 3.12 (2.74 to 3.51) |
| Senegal | 25 (17 to 34) | 0.35 (0.25 to 0.48) | 55 (39 to 78) | 0.38 (0.27 to 0.52) | 0.14 (0.08 to 0.21) |
| Serbia | 193 (133 to 249) | 2.03 (1.42 to 2.67) | 264 (193 to 336) | 2.30 (1.66 to 2.88) | 0.59 (0.46 to 0.73) |
| Seychelles | 0 (0 to 0) | 0.52 (0.39 to 0.68) | 1 (1 to 1) | 0.71 (0.54 to 0.90) | 1.10 (0.99 to 1.22) |
| Sierra Leone | 12 (8 to 16) | 0.29 (0.21 to 0.39) | 24 (17 to 34) | 0.29 (0.21 to 0.40) | 0.02 (−0.09 to 0.12) |
| Singapore | 329 (314 to 345) | 13.59 (12.99 to 14.25) | 897 (798 to 1007) | 11.18 (10.05 to 12.49) | −0.45 (−0.72 to −0.19) |
| Slovakia | 238 (154 to 286) | 4.55 (2.97 to 5.51) | 464 (329 to 582) | 6.87 (4.84 to 8.70) | 1.83 (1.61 to 2.06) |
| Slovenia | 156 (145 to 166) | 7.66 (7.14 to 8.20) | 262 (223 to 305) | 8.44 (7.25 to 9.69) | 0.59 (0.32 to 0.87) |
| Solomon Islands | 1 (1 to 1) | 0.31 (0.21 to 0.43) | 2 (1 to 3) | 0.29 (0.20 to 0.41) | −0.31 (−0.38 to −0.24) |
| Somalia | 16 (10 to 23) | 0.23 (0.16 to 0.32) | 39 (25 to 56) | 0.20 (0.14 to 0.29) | −0.38 (−0.44 to −0.32) |
| South Africa | 319 (181 to 565) | 0.91 (0.52 to 1.58) | 377 (212 to 572) | 0.62 (0.36 to 0.92) | −1.34 (−1.47 to −1.21) |
| South Korea | 3106 (1730 to 3631) | 8.93 (4.97 to 10.35) | 6404 (4309 to 7846) | 7.70 (5.22 to 9.49) | −0.31 (−0.93 to 0.30) |
| South Sudan | 16 (11 to 23) | 0.31 (0.22 to 0.43) | 22 (15 to 33) | 0.26 (0.18 to 0.37) | −0.54 (−0.58 to −0.50) |
| Spain | 13920 (13275 to 14586) | 31.85 (30.44 to 33.36) | 27442 (23642 to 32065) | 36.01 (31.43 to 41.58) | 0.41 (0.26 to 0.55) |
| Sri Lanka | 113 (79 to 177) | 0.74 (0.51 to 1.18) | 713 (407 to 1050) | 2.76 (1.62 to 4.05) | 5.78 (5.13 to 6.44) |
| Sudan | 65 (43 to 94) | 0.34 (0.23 to 0.48) | 159 (106 to 223) | 0.37 (0.25 to 0.52) | 0.33 (0.28 to 0.37) |
| Suriname | 2 (2 to 4) | 0.67 (0.50 to 1.22) | 71 (47 to 93) | 11.79 (7.85 to 15.39) | 8.83 (6.84 to 10.86) |
| Swaziland | 6 (4 to 9) | 0.83 (0.54 to 1.25) | 9 (5 to 19) | 0.79 (0.47 to 1.58) | −0.23 (−0.32 to −0.14) |
| Sweden | 4723 (4465 to 4971) | 37.64 (35.81 to 39.51) | 7404 (6526 to 8313) | 40.74 (36.45 to 45.69) | 0.53 (0.28 to 0.79) |
| Switzerland | 3863 (3550 to 4125) | 45.91 (41.82 to 49.21) | 5416 (4895 to 5933) | 37.26 (33.93 to 40.66) | −0.23 (−0.44 to −0.02) |
| Syria | 71 (48 to 101) | 0.58 (0.39 to 0.82) | 82 (56 to 117) | 0.59 (0.41 to 0.83) | 0.16 (0.13 to 0.20) |
| Taiwan (Province of China) | 1994 (1906 to 2080) | 10.55 (10.09 to 11.00) | 5114 (4234 to 6113) | 15.71 (13.27 to 18.49) | 0.71 (0.22 to 1.19) |
| Tajikistan | 28 (19 to 40) | 0.54 (0.38 to 0.75) | 69 (46 to 109) | 0.68 (0.45 to 1.05) | 0.90 (0.71 to 1.10) |
| Tanzania | 75 (55 to 106) | 0.32 (0.23 to 0.43) | 150 (98 to 219) | 0.29 (0.19 to 0.41) | −0.40 (−0.50 to −0.29) |
| Thailand | 310 (217 to 413) | 0.58 (0.41 to 0.79) | 970 (637 to 1277) | 1.15 (0.78 to 1.49) | 2.00 (1.78 to 2.22) |
| The Bahamas | 2 (1 to 3) | 0.83 (0.62 to 1.07) | 86 (66 to 110) | 20.44 (15.73 to 26.48) | 9.24 (6.78 to 11.77) |
| The Gambia | 3 (2 to 4) | 0.34 (0.24 to 0.47) | 8 (6 to 11) | 0.35 (0.26 to 0.49) | 0.08 (0.00 to 0.16) |
| Timor-Leste | 2 (2 to 3) | 0.34 (0.24 to 0.48) | 5 (4 to 8) | 0.42 (0.29 to 0.63) | 0.69 (0.63 to 0.75) |
| Togo | 10 (7 to 14) | 0.31 (0.22 to 0.42) | 25 (18 to 33) | 0.31 (0.23 to 0.42) | −0.03 (−0.11 to 0.05) |
| Tokelau | 0 (0 to 0) | 0.53 (0.39 to 0.75) | 0 (0 to 0) | 0.71 (0.48 to 1.13) | −0.23 (−0.67 to 0.21) |
| Tonga | 0 (0 to 1) | 0.51 (0.36 to 0.71) | 0 (0 to 1) | 0.48 (0.35 to 0.65) | −0.43 (−0.54 to −0.31) |
| Trinidad and Tobago | 10 (8 to 12) | 0.90 (0.74 to 1.09) | 356 (255 to 477) | 20.67 (14.97 to 27.72) | 9.27 (6.49 to 12.13) |
| Tunisia | 50 (34 to 71) | 0.61 (0.41 to 0.85) | 78 (54 to 110) | 0.65 (0.45 to 0.92) | 0.26 (0.22 to 0.29) |
| Turkey | 10745 (6199 to 19508) | 17.97 (10.65 to 31.35) | 23198 (16444 to 32617) | 29.07 (20.47 to 40.42) | 2.07 (1.80 to 2.35) |
| Turkmenistan | 19 (13 to 27) | 0.52 (0.36 to 0.72) | 26 (18 to 37) | 0.51 (0.34 to 0.72) | 0.12 (0.01 to 0.24) |
| Tuvalu | 0 (0 to 0) | 0.36 (0.26 to 0.50) | 0 (0 to 0) | 0.34 (0.24 to 0.47) | −0.40 (−0.49 to −0.31) |
| Uganda | 39 (27 to 56) | 0.26 (0.18 to 0.36) | 95 (62 to 139) | 0.25 (0.17 to 0.36) | −0.15 (−0.21 to −0.09) |
| Ukraine | 2930 (1877 to 4329) | 5.23 (3.42 to 7.63) | 4988 (3653 to 6403) | 8.94 (6.67 to 11.26) | 1.16 (0.68 to 1.63) |
| United Arab Emirates | 63 (36 to 103) | 4.67 (2.78 to 7.55) | 413 (316 to 560) | 7.56 (5.77 to 10.30) | 2.70 (2.08 to 3.32) |
| United Kingdom | 34186 (33323 to 34910) | 45.60 (44.60 to 46.57) | 55542 (51910 to 59019) | 51.21 (48.38 to 54.20) | 0.80 (0.46 to 1.14) |
| United States | 111652 (107616 to 114636) | 39.71 (38.48 to 40.72) | 210559 (199250 to 218997) | 41.36 (39.47 to 42.94) | 0.31 (0.04 to 0.58) |
| Uruguay | 120 (111 to 130) | 3.46 (3.19 to 3.78) | 1730 (1545 to 1905) | 37.87 (33.92 to 41.67) | 6.38 (4.75 to 8.02) |
| Uzbekistan | 105 (71 to 149) | 0.51 (0.34 to 0.72) | 178 (120 to 252) | 0.52 (0.36 to 0.73) | 0.13 (0.05 to 0.20) |
| Vanuatu | 1 (0 to 1) | 0.37 (0.26 to 0.50) | 1 (1 to 1) | 0.34 (0.23 to 0.47) | −0.29 (−0.35 to −0.24) |
| Venezuela | 601 (569 to 640) | 4.29 (4.08 to 4.52) | 3656 (2426 to 4965) | 12.10 (8.04 to 16.36) | 3.55 (3.09 to 4.02) |
| Vietnam | 356 (240 to 522) | 0.57 (0.38 to 0.87) | 861 (551 to 1330) | 0.83 (0.54 to 1.27) | 1.23 (1.19 to 1.26) |
| Virgin Islands, U.S. | 6 (5 to 8) | 6.39 (5.02 to 8.35) | 26 (19 to 35) | 19.79 (14.51 to 27.31) | 3.93 (3.02 to 4.86) |
| Yemen | 44 (29 to 63) | 0.35 (0.23 to 0.49) | 116 (77 to 164) | 0.35 (0.24 to 0.49) | 0.12 (0.09 to 0.15) |
| Zambia | 26 (19 to 37) | 0.36 (0.26 to 0.49) | 56 (37 to 81) | 0.32 (0.22 to 0.44) | −0.46 (−0.60 to −0.32) |
| Zimbabwe | 70 (46 to 93) | 0.90 (0.51 to 1.22) | 130 (93 to 192) | 1.02 (0.70 to 1.50) | 0.12 (−0.16 to 0.40) |

Abbreviations: ASR, age-standardized rate; MND, motor neuron disease; EAPC, estimated annual percentage change; SDI, sociodemographic index; UI, uncertainty interval; CI, confidence interval.

**Table S8.** Changes in incidence of MND according to aging, population and epidemiological change from 1990 to 2021 at global level by SDI regions.

|  | **Location** | **Overall difference** | **Aging**  **(aging percent)** | **Population (population percent)** | **Epidemiological change (epidemiological percent)** |
| --- | --- | --- | --- | --- | --- |
| **Incidence** | Global | 25510.56 | 9857.766  (38.642) | 16184.487  (63.442) | -531.695  (-2.084) |
|  | Low SDI | 1354.38 | -0.184  (-0.014) | 1561.52  (115.294) | -206.953  (-15.280) |
|  | Low-middle SDI | 2273.2 | 561.51  (24.701) | 1998.476  (87.915) | -286.785  (-12.616) |
|  | Middle SDI | 4188.66 | 1912.416  (45.657) | 2674.84  (63.859) | -398.597  (-9.516) |
|  | High-middle SDI | 4818.48 | 2232.501  (46.332) | 2095.161  (43.482) | 490.821  (10.186) |
|  | High SDI | 12736.63 | 5289.793  (41.532) | 4229.445  (33.207) | 3217.393  (25.261) |
| **Prevalence** | Global | 97692.89 | 22706.919  (23.243) | 78650.382  (80.508) | -3664.41  (-3.751) |
|  | Low SDI | 6802.2 | 97.678  (1.436) | 6948.223  (102.147) | -243.697  (-3.583) |
|  | Low-middle SDI | 10878.76 | 237.711  (2.185) | 10248.782  (94.209) | 392.268  (3.606) |
|  | Middle SDI | 16229.94 | 127.401  (0.785) | 14501.181  (89.348) | 1601.353  (9.867) |
|  | High-middle SDI | 18730.31 | 3711.045  (19.813) | 11271.982  (60.180) | 3747.284  (20.007) |
|  | High SDI | 46075.49 | 14987.688  (32.529) | 18683.554  (40.550) | 12404.247  (26.922) |
| **Deaths** | Global | 21521.01 | 7526.84  (34.974) | 9125.245  (42.402) | 4868.923  (22.624) |
|  | Low SDI | 27.12 | -0.276  (-1.018) | 10.147  (37.415) | 17.253  (63.617) |
|  | Low-middle SDI | 624.97 | 93.311  (14.930) | 158.032  (25.286) | 373.625  (59.783) |
|  | Middle SDI | 2938.33 | 831.558  (28.300) | 732.135  (24.917) | 1374.633  (46.783) |
|  | High-middle SDI | 6528.18 | 1760.761  (26.972) | 1277.371  (19.567) | 3490.052  (53.461) |
|  | High SDI | 11506.62 | 4831.746  (41.991) | 3447.159  (29.958) | 3227.711  (28.051) |
| **DALYs** | Global | 522380.34 | 166002.643  (31.778) | 248423.947  (47.556) | 107953.751  (20.666) |
|  | Low SDI | 2348.4 | 12.676  (0.540) | 1826.097  (77.759) | 509.624  (21.701) |
|  | Low-middle SDI | 21215.65 | 2360.816  (11.128) | 7219.106  (34.027) | 11635.731  (54.845) |
|  | Middle SDI | 88497.93 | 21428.493  (24.214) | 27062.132  (30.579) | 40007.3  (45.207) |
|  | High-middle SDI | 169325.85 | 40381.821  (23.849) | 38331.01  (22.637) | 90613.016  (53.514) |
|  | High SDI | 248597.54 | 98136.766  (39.476) | 84797.112  (34.110) | 65663.666  (26.414) |

Abbreviations: SDI, sociodemographic index; MND, motor neuron disease; DALYs, disability-adjusted life-years.

**Table S9.** The predicted case number and ASR of incidence, prevalence, deaths, and DALYs of MND from 2022 to 2035.

| **Year** | **Sex** | **Incidence** | | **Prevalence** | | **Deaths** | | **DALYs** | |
| --- | --- | --- | --- | --- | --- | --- | --- | --- | --- |
|  |  | **ASR** | **Number of cases** | **ASR** | **Number of cases** | **ASR** | **Number of cases** | **ASR** | **Number of cases** |
| 2022 | Female | 0.691436973 | 36012.43664 | 3.052118921 | 146100.4806 | 0.378266858 | 21888.10962 | 10.37692707 | 600904.2637 |
|  | Male | 0.907811784 | 27269.04477 | 3.682942624 | 120370.1433 | 0.551761716 | 14918.17229 | 15.14776623 | 409247.5524 |
| 2023 | Female | 0.695090786 | 36523.35547 | 3.052119736 | 147578.5135 | 0.37692208 | 22041.1153 | 10.36728663 | 607948.7997 |
|  | Male | 0.911802813 | 27685.32 | 3.68428645 | 121565.2879 | 0.55025478 | 15012.72727 | 15.1773959 | 412926.8488 |
| 2024 | Female | 0.698765901 | 37029.07351 | 3.05319303 | 149072.1727 | 0.375590714 | 22183.76849 | 10.35555511 | 614676.2367 |
|  | Male | 0.915749667 | 28101.07 | 3.686638081 | 122785.0285 | 0.548616983 | 15104.48769 | 15.2012866 | 416451.6022 |
| 2025 | Female | 0.702499235 | 37531.60777 | 3.055538422 | 150592.5193 | 0.37432022 | 22318.53825 | 10.34197707 | 621117.1079 |
|  | Male | 0.919723701 | 28517.15494 | 3.690316439 | 124036.096 | 0.546922709 | 15195.10227 | 15.22066757 | 419820.7593 |
| 2026 | Female | 0.706283582 | 38031.57111 | 3.059445089 | 152171.7851 | 0.3730425 | 22441.77297 | 10.32711402 | 627279.145 |
|  | Male | 0.923762305 | 28932.63384 | 3.696154403 | 125328.9852 | 0.545096175 | 15281.54178 | 15.2362054 | 423046.2331 |
| 2027 | Female | 0.710164163 | 38531.51015 | 3.065486574 | 153845.8914 | 0.37148363 | 22549.90565 | 10.31024796 | 633127.174 |
|  | Male | 0.927937313 | 29349.05093 | 3.705002542 | 126687.7804 | 0.543059402 | 15352.35448 | 15.24732164 | 426093.0191 |
| 2028 | Female | 0.71407028 | 39025.94806 | 3.073452779 | 155587.1638 | 0.369743342 | 22639.24875 | 10.29132731 | 638537.9337 |
|  | Male | 0.932115595 | 29763.45474 | 3.716122963 | 128105.8396 | 0.540727333 | 15411.42311 | 15.25116481 | 428957.0134 |
| 2029 | Female | 0.717993998 | 39514.09385 | 3.083135026 | 157382.3377 | 0.36803326 | 22713.36143 | 10.27108739 | 643531.8594 |
|  | Male | 0.936275716 | 30175.5115 | 3.729131723 | 129576.5378 | 0.538186927 | 15467.52743 | 15.24831253 | 431668.3933 |
| 2030 | Female | 0.721976857 | 39998.78448 | 3.094743727 | 159250.2795 | 0.366363098 | 22775.83341 | 10.24963281 | 648169.0302 |
|  | Male | 0.940491117 | 30586.75322 | 3.744450619 | 131109.691 | 0.535527999 | 15521.07599 | 15.24039351 | 434228.5852 |
| 2031 | Female | 0.726029544 | 40481.61439 | 3.108450656 | 161220.7519 | 0.364676555 | 22825.79623 | 10.22728897 | 652485.3171 |
|  | Male | 0.944801843 | 30997.4859 | 3.762736883 | 132713.8215 | 0.532732073 | 15569.692 | 15.22837811 | 436649.2358 |
| 2032 | Female | 0.730191303 | 40964.62278 | 3.124679752 | 163321.737 | 0.362736702 | 22860.44747 | 10.20353366 | 656468.6136 |
|  | Male | 0.94925581 | 31409.28109 | 3.784585265 | 134408.5368 | 0.529735443 | 15603.17 | 15.21206844 | 438906.4283 |
| 2033 | Female | 0.734437385 | 41445.21633 | 3.143439202 | 165533.2021 | 0.36068295 | 22876.62021 | 10.17872187 | 660026.9345 |
|  | Male | 0.953796471 | 31820.78927 | 3.809486305 | 136195.0228 | 0.526469435 | 15627.22213 | 15.18948185 | 441010.9973 |
| 2034 | Female | 0.738778179 | 41924.38677 | 3.164631322 | 167850.9592 | 0.358693595 | 22876.8623 | 10.15359584 | 663187.5046 |
|  | Male | 0.958440853 | 32232.43564 | 3.837270594 | 138070.9099 | 0.522992013 | 15649.57998 | 15.16124735 | 442995.1151 |
| 2035 | Female | 0.743253073 | 42405.6435 | 3.188462914 | 170299.0638 | 0.356743174 | 22864.61096 | 10.1279708 | 666016.3 |
|  | Male | 0.963263715 | 32645.97884 | 3.868421639 | 140047.1746 | 0.519380165 | 15669.26602 | 15.12886688 | 444851.8714 |
| 2036 | Female | 0.747867225 | 42890.44404 | 3.215038705 | 172906.7681 | 0.354782786 | 22840.81203 | 10.10177744 | 668536.7986 |
|  | Male | 0.968296098 | 33061.60893 | 3.903548977 | 142129.978 | 0.515654936 | 15684.18742 | 15.09290911 | 446577.9536 |
| 2037 | Female | 0.752645946 | 43379.87992 | 3.244659931 | 175693.7242 | 0.35263454 | 22803.29014 | 10.07456931 | 670736.9417 |
|  | Male | 0.973560958 | 33480.39058 | 3.943038816 | 144334.1087 | 0.511767046 | 15686.44887 | 15.05313755 | 448152.9692 |
| 2038 | Female | 0.757595996 | 43873.83485 | 3.277537451 | 178651.5147 | 0.350439684 | 22749.83838 | 10.04704397 | 672557.667 |
|  | Male | 0.97905356 | 33902.55312 | 3.986644936 | 146670.3733 | 0.507667277 | 15682.23704 | 15.00826132 | 449606.9721 |
| 2039 | Female | 0.762724115 | 44373.05524 | 3.313613589 | 181775.2755 | 0.348323741 | 22681.12251 | 10.01980038 | 674011.6936 |
|  | Male | 0.984789783 | 34328.31705 | 4.034214752 | 149137.5133 | 0.503371643 | 15677.186 | 14.95862355 | 450966.3161 |
| 2040 | Female | 0.768043045 | 44879.21281 | 3.353019963 | 185082.5665 | 0.346235972 | 22598.99988 | 9.992292902 | 675136.6944 |
|  | Male | 0.990807534 | 34758.13464 | 4.086105569 | 151742.4318 | 0.498922729 | 15669.0652 | 14.9051305 | 452205.7253 |
| 2041 | Female | 0.773527932 | 45391.80596 | 3.395789157 | 188598.1454 | 0.34412549 | 22505.19424 | 9.963917402 | 675924.3434 |
|  | Male | 0.99709676 | 35190.7746 | 4.142831414 | 154487.5703 | 0.494359186 | 15655.59824 | 14.84765716 | 453297.1037 |
| 2042 | Female | 0.779195967 | 45911.2666 | 3.442178897 | 192338.7895 | 0.341874113 | 22398.59645 | 9.934174897 | 676363.2454 |
|  | Male | 1.003666768 | 35626.95845 | 4.204720661 | 157385.7794 | 0.489655994 | 15631.41409 | 14.78598528 | 454217.4899 |
| 2043 | Female | 0.785096614 | 46440.32599 | 3.492649474 | 196314.3884 | 0.33960566 | 22278.707 | 9.904044294 | 676436.5059 |
|  | Male | 1.010578759 | 36068.80169 | 4.271958619 | 160458.8264 | 0.484802541 | 15602.11697 | 14.71980116 | 455010.2539 |
| 2044 | Female | 0.791250401 | 46980.14927 | 3.547190271 | 200518.8134 | 0.337399017 | 22145.19388 | 9.873967026 | 676152.707 |
|  | Male | 1.017862996 | 36517.00969 | 4.344402546 | 163706.434 | 0.479793567 | 15571.30733 | 14.6493962 | 455693.6076 |
| 2045 | Female | 0.797657973 | 47531.28548 | 3.605845257 | 204959.7569 | 0.335193708 | 21998.49904 | 9.843074196 | 675519.9577 |
|  | Male | 1.025537891 | 36971.39844 | 4.422224112 | 167130.7078 | 0.474640946 | 15536.20793 | 14.57505947 | 456225.889 |
| 2046 | Female | 0.804263716 | 48091.1917 | 3.668477391 | 209649.6571 | 0.332922947 | 21839.66424 | 9.810264875 | 674497.1121 |
|  | Male | 1.033554462 | 37429.20798 | 4.505697011 | 170725.3485 | 0.469368332 | 15493.72672 | 14.49599138 | 456554.7803 |

Abbreviations: ASR, age-standardized rate; DALYs, disability-adjusted life-years; MND, motor neuron disease.

**Table S10.** Frontier analysis according to the ASR of incidence, prevalence, deaths, and DALYs and SDI in 2021.

|  | Top 15 countries with the largest effective difference | 5 examples of countries with low SDI and low effective difference | 5 examples of countries high SDI and relatively high effective difference |
| --- | --- | --- | --- |
| **Incidence** | Andorra, Austria, Australia, Belgium, Canada, Denmark, Spain, Finland, Malta, Italy, Germany, Iceland, Ireland, New Zealand, and Sweden | Somalia, Burkina Faso, Benin, Guinea, and Togo | Belgium, Andorra, Sweden, Canada, and Ireland |
| **Prevalence** | Spain, Austria, Italy, Luxembourg, New Zealand, Belgium, Switzerland, France, Denmark, United Kingdom, Finland, Netherlands, Canada, Sweden, and Andorra | Central African Republic, Democratic Republic of Congo, Guinea-Bissau, Uganda, and Rwanda | United Kingdom, Finland, Netherlands, Sweden, and Canada |
| **Deaths** | Uruguay, Italy, Switzerland, Andorra, Lithuania, Canada, Sweden, New Zealand, Denmark, Australia, Belgium, Netherlands, United Kingdom, Finland, and Ireland | Central African Republic, Niger, Malawi, Madagascar, and Mozambique | Belgium, Netherlands, Ireland, United Kingdom, and Finland |
| **DALYs** | Luxembourg, Poland, Portugal, Costa Rica, Spain, Austria, Iceland, Malta, Sweden, Switzerland, France, United Kingdom, Denmark, Netherlands, and Belgium | Somalia, Central African Republic, Uganda, Democratic Republic of Congo, and Rwanda | Sweden, Denmark, Netherlands, United Kingdom, and Belgium |

Abbreviations: ASR, age-standardized rate; DALYs, disability-adjusted life-years; SDI, sociodemographic index.
